# Development of a fluorescent probe library enabling efficient screening of tumour-imaging probes based on discovery of biomarker enzymatic activities†

**DOI:** 10.1039/d1sc06889j

**Published:** 2022-03-21

**Authors:** Yugo Kuriki, Takafusa Yoshioka, Mako Kamiya, Toru Komatsu, Hiroyuki Takamaru, Kyohhei Fujita, Hirohisa Iwaki, Aika Nanjo, Yuki Akagi, Kohei Takeshita, Haruaki Hino, Rumi Hino, Ryosuke Kojima, Tasuku Ueno, Kenjiro Hanaoka, Seiichiro Abe, Yutaka Saito, Jun Nakajima, Yasuteru Urano

**Affiliations:** Graduate School of Pharmaceutical Sciences, The University of Tokyo 7-3-1, Hongo Bunkyo-ku Tokyo Japan; Graduate School of Medicine, The University of Tokyo 7-3-1, Hongo Bunkyo-ku Tokyo Japan mkamiya@m.u-tokyo.ac.jp uranokun@m.u-tokyo.ac.jp; Department of Thoracic Surgery, Graduate School of Medicine, The University of Tokyo 7-3-1, Hongo Bunkyo-ku Tokyo Japan; Endoscopy Division, National Cancer Center Hospital 5-1-1, Tsukiji Chuo-ku Tokyo Japan; Institute of Engineering, Tokyo University of Agriculture and Technology 2-24-16 Naka-cho Koganei-shi Tokyo Japan; Daito Bunka University, Department of Sports and Health Science 560, Iwadono Higashimatsuyama Saitama Japan; PRESTO, Science and Technology Agency (JST) 4-1-8 Honcho Kawaguchi-shi Saitama Japan; CREST, Agency for Medical Research and Development (AMED) 1-7-1 Otemachi Chiyoda-ku Tokyo Japan

## Abstract

Fluorescent probes that can selectively detect tumour lesions have great potential for fluorescence imaging-guided surgery. Here, we established a library-based approach for efficient screening of probes for tumour-selective imaging based on discovery of biomarker enzymes. We constructed a combinatorial fluorescent probe library for aminopeptidases and proteases, which is composed of 380 probes with various substrate moieties. Using this probe library, we performed lysate-based *in vitro* screening and/or direct imaging-based *ex vivo* screening of freshly resected clinical specimens from lung or gastric cancer patients, and found promising probes for tumour-selective visualization. Further, we identified two target enzymes as novel biomarker enzymes for discriminating between tumour and non-tumour tissues. This library-based approach is expected to be an efficient tool to develop tumour-imaging probes and to discover new biomarker enzyme activities for various tumours and other diseases.

## Abstract 2

Efficient methodology to develop tumor-imaging fluorescent probes based on screening with our newly constructed probe library for aminopeptidase/protease (380 probes) and clinical samples has been established.

## Graphical abstract

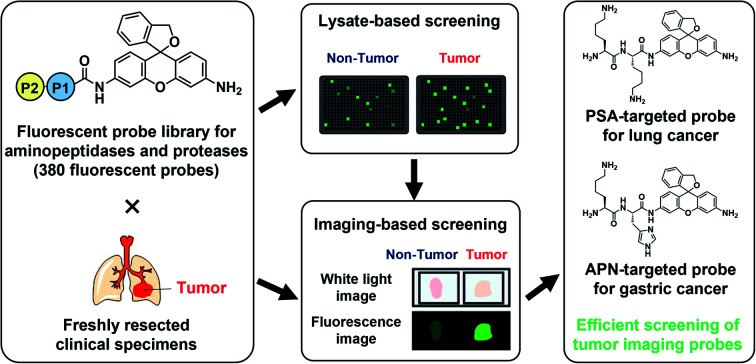

## Introduction

Complete surgical resection of tumour lesions is critical for the patients' prognosis. However, the ability to detect tiny tumour foci and to accurately delineate the border between tumour and non-tumour tissues with the unaided human eye is quite limited, and thus recurrence due to tumour cells left behind after surgery is a serious problem. In order to ensure complete resection of tumour lesions, intraoperative fluorescence imaging has attracted considerable attention, because of its high sensitivity, high spatiotemporal resolution, low cost and real-time capability.^1,2^ So far, various types of fluorescent imaging probes have been developed by targeting well-validated tumour biomarkers, and there are several successful examples of tumour visualization in mouse models and/or patients' specimens.^3–6^ In particular, activatable fluorescent probes (fluorogenic substrates) targeting enzymes characteristic of tumours are promising,^7,8^ since they have a high tumour-to-normal intensity ratio (T/N). We and other groups have focused on cancer-associated proteases as imaging targets,^9–13^ since they exhibit altered expression levels in the pathological context, and some of them are known to be related to malignant tumour phenotypes such as reconstruction of extracellular matrixes,^14^ generation of peptide messengers,^15^ and utilization of amino acids from degradation of proteins.^16^ For example, fluorescent probes have been developed targeting γ-glutamyl transferase (GGT),^17,18^ which has been shown to visualize breast,^19,20^ hepatic^21^ and oral cancers.^22^ However, available biomarker enzymes are still limited, and finding new biomarker enzymes is important to broaden the applicability of intraoperative tumour imaging.

To uncover new biomarker enzymes, it is important to evaluate altered enzyme activities between tumour and non-tumour tissues, rather than just altered protein expression levels, since the activities are required for fluorescence activation. Considering that the enzyme activities in living systems are dynamically modified by many factors, including protein–protein interactions and posttranslational modifications,^23,24^ we thought that an efficient strategy would be to prepare a combinatorial library of fluorogenic probes for proteases that would be suitable not only for *in vitro* screening, but also for *ex vivo* screening of patients' specimens to directly evaluate altered enzyme activities under pathophysiological conditions. So far, aminocoumarin-based fluorogenic substrate libraries for proteases have been developed for *in vitro* screening of specific substrates or optimal inhibitors.^25–29^ However, their use is mainly limited to *in vitro* biochemical assay due to the relatively short wavelengths of the fluorescence emission, and especially they cannot be applied for imaging-based screening on living samples.

In this study, we aimed at developing new fluorogenic substrate libraries for proteases making use of our previously reported scaffold fluorophore, HMRG (hydroxymethyl rhodamine green) (Fig. S1†). HMRG is an excellent scaffold fluorophore for developing fluorogenic substrates for proteases because high activation ratios can be achieved upon one-step enzymatic reaction, and the hydrolysis product, HMRG, emits bright fluorescence in the visible wavelength region.^30,31^ Thus, it is suitable for both *in vitro* screening and *ex vivo* screening using patients' specimens. Further, the selected probes can be directly used to provide intraoperative fluorescence guidance for tumour-selective imaging. Here, we used solid-phase synthesis to construct a fluorescent probe library composed of 380 HMRG-based probes for aminopeptidases and proteases, and we performed *in vitro*/*ex vivo* screening with resected specimens from lung or gastric cancer patients. We found promising probes for tumour-selective visualization of lung and gastric cancers, and identified two main target enzymes as novel biomarker enzymes for discriminating between tumour and non-tumour tissues in the local tumour environment.

## Results and discussion

### Construction of a fluorescent probe library for aminopeptidases and proteases

**Fig. 1:**
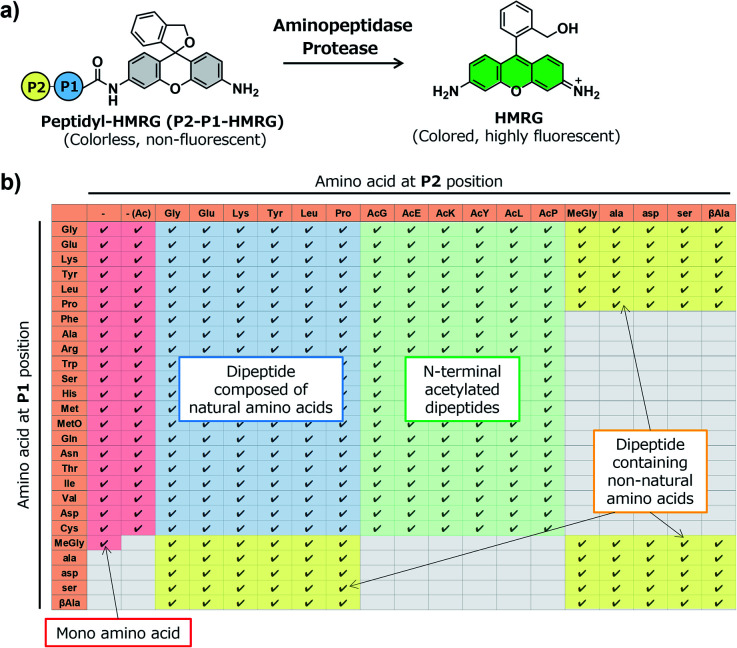
Construction of a fluorescent probe library for aminopeptidases and proteases based on the HMRG scaffold. (a) Reaction scheme of peptidyl-HMRG with aminopeptidases or proteases. (b) List of the fluorescent probes in the library. Columns and rows represent amino acids at the P1 and P2 positions, respectively. Abbreviations: MetO; methionine sulfoxide.

**Scheme 1:**
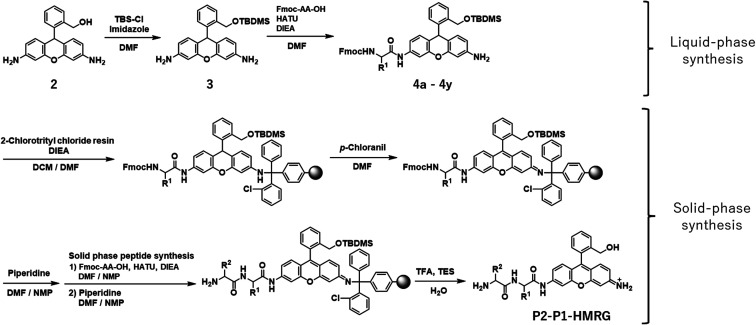
Synthesis of P2–P1-HMRG.

In order to construct the fluorescent probe library based on the HMRG scaffold, we set out to install various amino acids or dipeptides onto HMRG by using solid-phase peptide synthesis (SPPS) (Fig. 1 and Scheme 1). We focused on aminopeptidases and dipeptidylpeptidases as target enzymes because they generally have high reaction rates, and we selected dipeptide structures as candidate substrates because they provide sufficient substrate diversity for a screening library. We first planned to synthesize compound 3 with a reduced xanthene ring in order to improve the nucleophilicity of the amino group^32^ and included TBDMS protection on the hydroxyl group to reduce side reactions. This compound was linked to 2-chlorotrityl chloride resin, and coupled with Fmoc-amino acid (Fmoc-AA-OH). However, the condensation reaction between compound 3 on the resin and Fmoc-AA-OH did not proceed efficiently under any of the conditions we tried, probably due to insufficient reactivity of the amino group on the resin (data not shown). Thus, we next tried the approach illustrated in Scheme 1, in which we first incorporated a variety of Fmoc-AA-OH onto compound 3 in the liquid phase to prepare compounds 4a–4y, which were linked to the resin as starting materials for SPPS. After oxidation with *p*-chloranil to form a xanthene fluorophore form, second amino acids were incorporated, followed by cleavage from the resin with TFA/TES/H_2_O and simple purification by ether precipitation (if the purities determined from the absorbance at 254 and 490 nm in LC-MS analysis were lower than 80%, we further purified the compounds by HPLC). After optimizing each step, we synthesized 380 peptidyl-HMRGs with various substrate moieties as a probe library for aminopeptidases and proteases (Fig. 1b and Table S1†). The average amount of the synthesized probes was approximately 5 μmol and the average HPLC purities determined by monitoring at 254 and 490 nm were 93% and 95%, respectively. The prepared dipeptidyl-HMRGs were designated as P2–P1-HMRG, where P1 and P2 represent the amino acid at the corresponding position. To ensure the diversity of the library, we selected 20 proteogenic amino acids and 6 unnatural amino acids (d-alanine, d-aspartate, d-serine, β-alanine (βAla), *N*-methylglycine (MeGly) and methionine sulfoxide (MetO)) as P1 amino acids. As P2 amino acids, we selected six representative natural amino acids with different properties (glycine (no side chain), glutamate (acidic), lysine (basic), tyrosine (aromatic), leucine (aliphatic/hydrophobic), and proline (cyclic)), together with the above-mentioned unnatural amino acids except methionine sulfoxide. In addition, we synthesized N-terminally acetylated derivatives of a part of the probe library to target endopeptidases (Fig. 1 and Table S1†). The prepared fluorescence probe libraries for aminopeptidases and proteases were dispensed into each well of 384 microplates to prepare stock plates for high-throughput screening.

### Screening of imaging probes for lung cancer with the library and clinical specimens

**Fig. 2:**
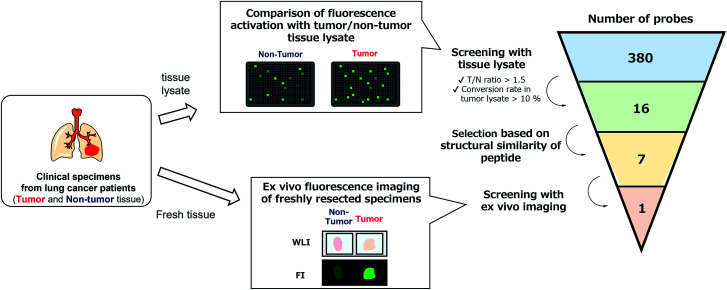
Flowchart of the screening of fluorescent probes for lung adenocarcinoma and squamous cell carcinoma. By performing lysate-based and imaging-based screenings, one probe (KK-HMRG) was identified as a fluorescent probe for lung adenocarcinoma and squamous cell carcinoma.

With the constructed libraries in hand, we next performed screening of probes suitable for detecting lung cancer (Fig. 2), which has been the leading cause of cancer-related death in the world. Although we previously examined the ability of our developed fluorogenic probe for GGT, gGlu-HMRG, to visualize lung cancer, its sensitivity and specificity were not sufficient.^33^ Therefore, fluorescent probes targeting other biomarker enzymes are needed.

As a first screening, we performed high-throughput screening of probes using tissue lysate of resected specimens from lung cancer patients. All specimens, including lung tumour and normal lung tissues, were obtained from the Department of Thoracic Surgery, Graduate School of Medicine, University of Tokyo. Before the study, all the patients provided written informed consent for this *ex vivo* lung cancer fluorescence imaging study. The Research Review Board at our institution examined and approved the research protocol, which was in accordance with the Declaration of Helsinki. We prepared five pairs of lysates of tumour and adjacent non-tumour tissues from five patients with lung adenocarcinoma (the most common subtype of lung cancer) and examined their reactivities with the probe libraries by measuring the time-dependent fluorescence increase after application to the 384-well plates. In order to perform tumour-selective imaging, we thought it important that the reactivity of probes in tumour lysate should be sufficiently strong and higher than that in non-tumour lysate. Thus, we set two criteria for selecting candidate probes: (1) more than 10% of the probe was converted to the hydrolysis product HMRG after one-hour incubation (the conversion rate was calculated by dividing the fluorescence increase of each probe by the fluorescence intensity of HMRG at same concentration); (2) the tumour-to-normal ratio of the conversion rate was more than 1.5. Considering tumour heterogeneity, we picked up 16 probes that met the criteria in at least three pairs of lysates out of the five. Among these 16 candidate probes, we further selected 7 probes for the second screening from the viewpoints of similarity of substrate peptide sequences: KK-, GP-, EP-, PP-, KR-, KA- and KM-HMRG (Fig. 2, S2 and S3†). (Important note: X-MeGly-HMRGs (X = amino acids except βAla) were found to be chemically unstable in aqueous buffer at physiological pH, probably due to the intramolecular nucleophilic attack of the N-terminal amino group to release HMRG).

**Fig. 3:**
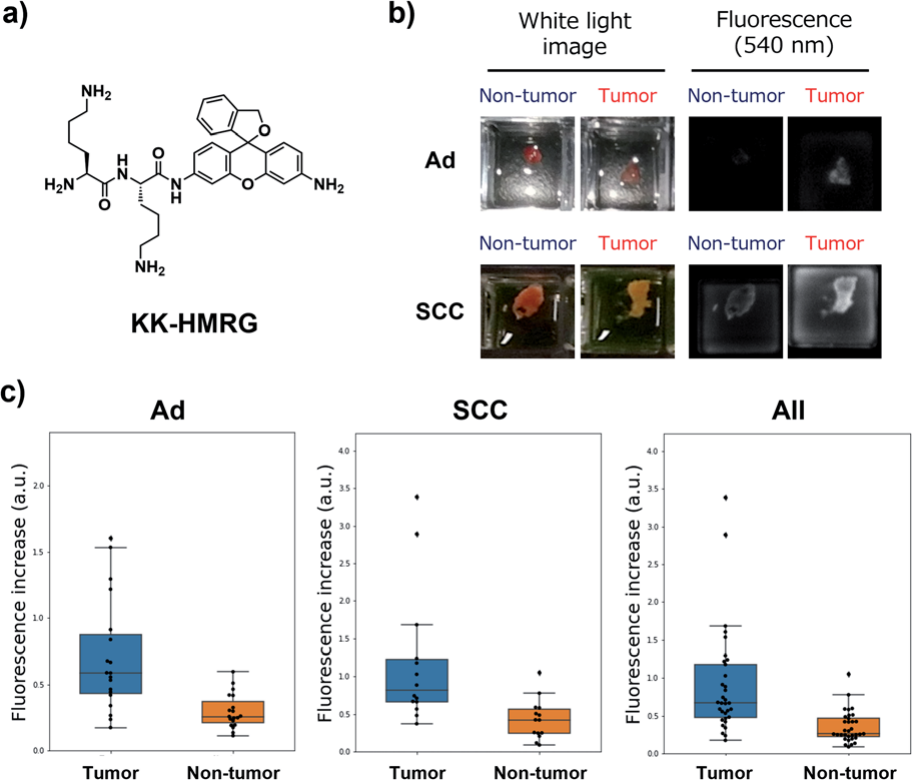
Fluorescent imaging of lung cancer with KK-HMRG. (a) Chemical structure of KK-HMRG. (b) Representative images of fluorescence imaging with KK-HMRG in lung adenocarcinoma (Ad) and squamous cell carcinoma (SCC). 50 μM KK-HMRG in phosphate-buffered saline was applied to the tissues, and the fluorescence increase was monitored with a Maestro Imaging system. (c) Fluorescence increase of KK-HMRG after incubation with the tissues for 30 min and ROC curve calculated from the results of KK-HMRG and all tissues. The dot plots represent the results for adenocarcinoma, squamous cell carcinoma and all samples (*n* = 19, 13 and 32, respectively).

Next, we performed *ex vivo* imaging-based screening with the 7 candidate probes. We applied the probes to small pieces of lung tumour (adenocarcinoma) and non-tumour tissues, and monitored the fluorescence increase *ex vivo*. After testing at least 7 specimens from different patients, we found 5 probes (KK-, GP-, PP-, KR-, KA-) having the ability to distinguish tumour tissues with relatively high accuracy (Fig. 3, Table S2 and Fig. S4†). We also examined the reactivities of these probes with squamous cell carcinoma (the second-most common subtype of lung cancer), aiming at finding a probe applicable for detecting a wide range of lung tumours (Fig. 3, Table S2 and Fig. S4†). As a result, KK-HMRG showed the best ability to distinguish tumour and non-tumour tissue both in adenocarcinoma and squamous cell carcinoma; the sensitivity, specificity and AUC were calculated to be 0.727, 0.848 and 0.842, respectively (Fig. 3 and Table S2†). (Important note: the fluorescence intensities at reddish non-tumour tissues tend to be undervalued, meaning that the high accuracy of KK-HMRG results not only from the difference in enzyme activities, but also from the difference in colour between tumour and non-tumour tissues.) In order to fully characterize KK-HMRG, we carried out a liquid-phase synthesis and measured the ^1^H and ^13^C NMR spectra and HRMS data (Fig. S5†). KK-HMRG synthesized by solid-phase synthesis and KK-HMRG synthesized by liquid-phase synthesis were identical by LC-MS analysis (Fig. S6†).

## Target identification of KK-HMRG

**Fig. 4:**
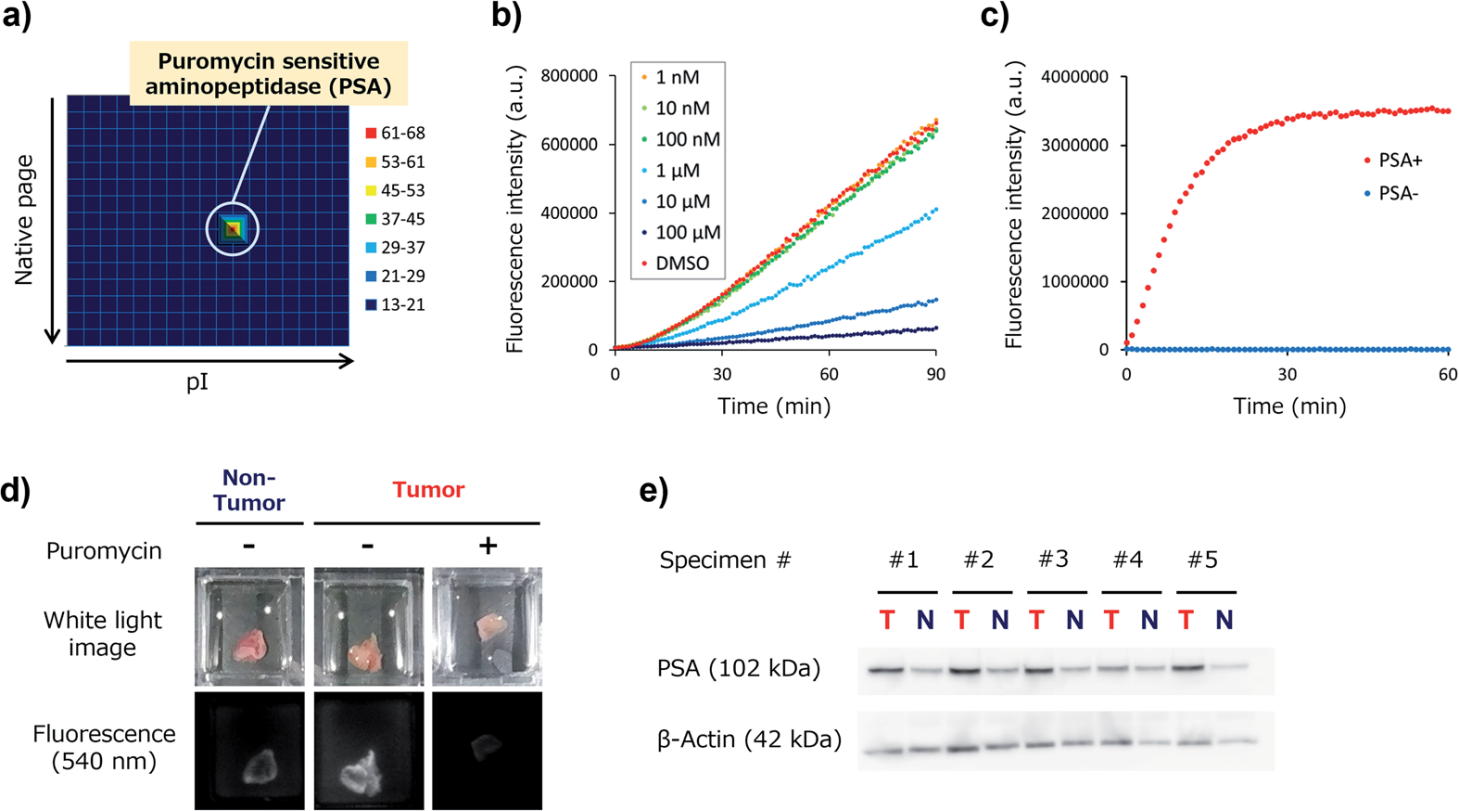
Target identification of KK-HMRG. (a) Result of the DEG assay of lung adenocarcinoma lysate (total protein = 26 μg) with KK-HMRG (1 μM in phosphate-buffered saline). The protein in the well with the strongest activity was analyzed by LC-MS/MS after trypsinization and identified as PSA. (b) Fluorescence increase of 1 μM KK-HMRG and 500 ng lung adenocarcinoma lysate in the presence or absence of puromycin at various concentrations. Puromycin, a PSA inhibitor, suppressed the fluorescence increase of KK-HMRG with lung adenocarcinoma lysate in a concentration-dependent manner. (c) Fluorescence increase of 1 μM KK-HMRG with or without 2.5 ng PSA. (d) Fluorescence imaging of lung adenocarcinoma and non-tumour tissue with 50 μM KK-HMRG in the presence or absence of 500 μM puromycin. (e) Expression level of PSA in the samples from lung adenocarcinoma tissues and non-tumour tissues from five patients. KK-HMRG in the presence or absence of 500 μM puromycin. (e) Expression level of PSA in the samples from lung adenocarcinoma tissues and non-tumour tissues from five patients.

We next set out to identify the target enzyme of KK-HMRG in lung tumour specimens. For this purpose, we used our previously developed zymography method termed diced electrophoresis gel (DEG) assay^34^ (Fig. S7†). In the assay, we obtained only one fluorescent spot for lung adenocarcinoma lysate (Fig. 4a) and for squamous cell carcinoma lysate (Fig. S8†), and these seemed to be located at the same position. Peptide mass fingerprinting analysis of the fluorescent spot yielded a list of candidate proteins. Among them, we looked for proteins with peptidase activities in protein/enzyme databases and focused on four candidate enzymes based on the reliability of the MS/MS analysis and biochemical knowledge of substrate specificities: they were puromycin-sensitive aminopeptidase (PSA), calpain-2, dipeptidylpeptidase-3 (DPP-3) and leukotriene A4 hydrolase (Table S3†). To identify the main contributor to the hydrolysis of KK-HMRG, we next examined the reactivity of KK-HMRG with lung tumour lysate in the presence of appropriate inhibitors (puromycin for PSA,^35^ SNJ-1945 for calpain-2,^36^ 3,4-dichloroisocoumarin for DPP-3 ^37^ or SC-57461A for leukotriene A4 hydrolase^38^). Puromycin strongly inhibited the fluorescence increase of KK-HMRG with tumour lysate in a concentration-dependent manner (Fig. 4b), while the other inhibitors had little or no inhibitory effect on the fluorescence increase (Fig. S9†), suggesting that PSA is likely the responsible enzyme. We also confirmed that KK-HMRG was hydrolyzed to HMRG by recombinant PSA protein by means of fluorescence measurement and LC-MS analysis (Fig. 4c and S10†), and that the two lysine residues of KK-HMRG were hydrolyzed in two steps upon reaction with PSA and with tissue lysate of lung adenocarcinoma (Fig. S10 and S11†). We also confirmed that puromycin inhibited the *ex vivo* fluorescence increase of resected lung adenocarcinoma tissue (Fig. 4d), and that PSA expression was upregulated in tumour tissues compared to non-tumour tissues in 4 out of the 5 adenocarcinoma lysates (Fig. 4e). These results strongly suggest that main target enzyme of KK-HMRG in lung cancer is PSA. Thus, the screening with our probe libraries led to the discovery of PSA activity as a biomarker in lung cancer. Our results suggest that KK-HMRG can be used as a lung tumour-selective imaging probe.

### Screening of imaging probes on ESD samples of gastric cancer

**Fig. 5:**
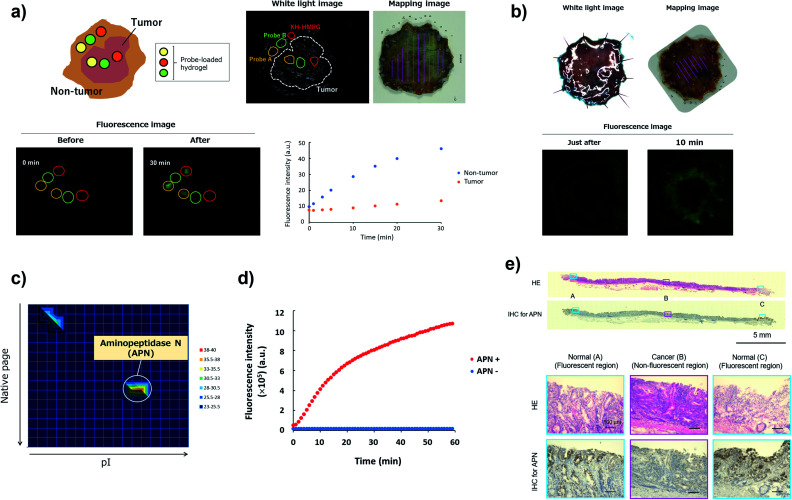
Screening of fluorescent probe for gastric cancer and target identification of KH-HMRG. (a) Scheme and a representative case of live imaging-based screening with Tetra-PEG gel. Pieces of Tetra-PEG gel loaded with three different probes were put on both tumour and non-tumour regions of a gastric cancer sample, and the fluorescence increase was monitored. KH-HMRG showed a selective fluorescence increase in non-tumour regions (in the red circles), compared to the non-selective fluorescence increase of probe A (LL-HMRG, yellow circles) or of probe B (sK-HMRG, green circles). In the mapping image, pink lines indicate the tumour region. (b) Fluorescence imaging of an ESD sample of gastric cancer with 50 μM KH-HMRG. The tumour region was visualized by negative staining. (c) Result of the DEG assay of dHL60 lysate (total protein = 15 μg) with KH-HMRG (1 μM in phosphate-buffered saline). The protein in the well with the strongest activity was analyzed by LC-MS/MS after trypsinization and identified as APN. (d) Fluorescence increase of 1 μM KK-HMRG with or without 250 ng APN. (e) Immunohistochemical staining of APN in the sample in Fig. 5b. Inflammation was confirmed in Normal (A and C) and intestinal metaplasia was confirmed in Normal (A and C).

To confirm the versatility of this approach, we next applied our probe libraries to endoscopic submucosal dissection (ESD) specimens from gastric cancer patients. In contrast to the case of lung cancer specimens, it was impossible to prepare tissue lysate of ESD specimens due to their small size. Therefore, we selected several probes as a focused library, and applied them to ESD specimens to perform *ex vivo* imaging-based screening. To evaluate the reactivities of a few probes simultaneously on one specimen, we used Tetra-PEG gels^39^ or medical gauzes that were pre-soaked with probe solutions, and measured the fluorescence increase at tumour regions and non-tumour regions (Fig. 5a and S12†). As a result, we found that KH-HMRG showed a selective fluorescence increase at non-tumour regions in 3 of 4 specimens (Fig. 5a, S13 and S14†). Since a fluorescent probe enabling negative staining of tumour region could also be a useful diagnostic tool, we next examined whether negative staining of tumour regions could be achieved by spraying KH-HMRG onto ESD specimens. In 12 specimens out of the tested 25 specimens, tumour regions were clearly visualized (Fig. 5b and S15†). In 11 cases out of the 13 failed cases, no fluorescent signal activation was observed in non-tumour regions, meaning that there is heterogeneity of enzyme activity in non-tumour regions (Fig. S16†). To identify the target enzyme of KH-HMRG, we performed DEG assay using lysate of differentiated HL60 cells,^40^ which were found to have enzyme activity to hydrolyze KH-HMRG (Fig. S17†). (Note: as mentioned above, lysates of clinical ESD specimens were not available.) We observed one strongly fluorescent spot and one weakly fluorescent spot, and PMF analysis of the strongly fluorescent spot identified aminopeptidase N (APN) and Xaa-Pro aminopeptidase as candidates (Fig. 5c and Table S4†). Judging from their substrate specificity, we focused on APN, which is a membrane-bound alanylaminopeptidase with broad substrate specificity,^41^ whereas Xaa-Pro aminopeptidase cleaves N-terminal amino acids when the amino acid at P1′ is proline.^42^ We confirmed that recombinant APN protein hydrolyzes KH-HMRG to HMRG (Fig. 5d). Further, the fluorescence increase of KH-HMRG in dHL60 lysate was inhibited by the APN inhibitor bestatin^41^ in a concentration-dependent manner (Fig. S18†). LC-MS analysis of the reaction solution of KH-HMRG with APN or with dHL60 cell lysate revealed that KH-HMRG was hydrolyzed in two steps (Fig. S19†). Additionally, to examine the selectivity of KH-HMRG for APN, we performed live-cell imaging of APN activity in HT1080 (APN-positive) and HEK293 (APN-negative) cells (Fig. S20†). As expected, a fluorescence signal was detected only from HT1080 cells when KH-HMRG was applied (Fig. S21†). Interestingly, when we applied A-HMRG (alanyl-HMRG, alanine is a standard sequence for fluorogenic substrates of APN^12^), fluorescence activation was observed with both HT1080 and HEK293 cells (Fig. S22†). This result demonstrated that Lys–His is a highly selective sequence for APN when using HMRG as a fluorophore. Finally, we confirmed that the expression level of APN was higher in non-tumour regions, especially on the apical membrane, by immunohistochemistry (Fig. 5e). In addition, the fluorescence increase of KH-HMRG on ESD samples of gastric non-tumour tissue was inhibited by bestatin (Fig. S23†). These data strongly suggest that the responsible enzyme for the fluorescence increase in non-tumour regions was APN. It should be noted that inflammation and intestinal metaplasia were observed in non-tumour regions (Fig. 5e), which might be related to this partial upregulation of APN activity in these regions. Further studies are underway. The present results demonstrate that our library-based approach is a versatile tool for discovering novel fluorescent probe/biomarker enzyme activity pairs for tumour-selective imaging.

## Conclusions

We have established a library-based approach for efficient screening of fluorescent probes suitable for tumour imaging and novel biomarker enzyme activities. To construct the fluorescent probe library based on the HMRG scaffold, we devised a simple synthetic scheme using SPPS to prepare 380 peptidyl-HMRGs with various substrate moieties. With the library in hand, we performed screening of probes for lung and gastric cancer detection. For lung cancer, we found several probes with good ability to distinguish tumour and non-tumour tissues, and among them, KK-HMRG targeting PSA exhibited particularly high accuracy. For gastric cancer, we identified KH-HMRG targeting APN as a negative-staining probe due to its selective fluorescence increase in non-tumour regions. The reactivities of these probes were similar or superior to those of the aminomethyl coumarin-based substrates (Fig. S24, Tables S4 and S5†). The greatest advantage of this strategy is the ability to find new biomarker enzyme activities for tumour imaging directly from clinical specimens. Furthermore, the selected probes can be directly used for intraoperative fluorescence imaging guidance. Unfortunately, the sensitivity/specificity of the selected probes in this study are not high enough for clinical use, probably due to the heterogeneity of both tumour and non-tumour tissues. However, we expect that we would be able to overcome the heterogeneity by targeting multiple enzyme activities^11,31,43,44^ that are altered at tumour lesions, and these could also be determined by the devised library-based screening. In addition, this methodology should be applicable not only for finding imaging probes for other diseases,^45^ but also finding probes to detect a specific subset of cells.^46^ Therefore, we believe that our approach will accelerate research into characteristic enzyme activities in specific environments, with many potential medical and biological applications.

## Author contributions

Y. K., M. K. and Y. U. co-wrote and reviewed the manuscript. Y. K., A. N. and K. T. synthesized and analysed the compounds. Y. K. and T. Y. performed biochemical experiments. T. Y., H. H., J. N., Y. K., H. T., A. S. and Y. S. performed cancer imaging and analysed the data. Y. A. prepared tetra-PEG gels and supervised the related experiments. K. F. and R. H. performed histological analyses. T. K., R. K., T. U., and K. H. discussed the results and reviewed and commented the manuscript. M. K. and Y. U. supervised the entire project.

## Conflicts of interest

There are no conflicts to declare.

## Supplementary Material

SC-013-D1SC06889J-s001
