# Supplementary material for: Development of a fluorescent probe library enabling efficient screening of tumour-imaging probes based on discovery of biomarker enzymatic activities
Source: Chem Sci. 2022 Mar 21;13(16):4474–81. doi: 10.1039/d1sc06889j (PMC9019911; doi:10.1039/d1sc06889j)
Supplement: SC-013-D1SC06889J-s001 [file SC-013-D1SC06889J-s001.pdf]

Electronic Supplementary Material (ESI) for Chemical Science.
This journal is © The Royal Society of Chemistry 2022

Supporting Information

Development of a Fluorescent Probe Library Enabling Efficient Screening of Tumour-Imaging Probes Based on Discovery of Biomarker Enzymatic Activities

Yugo Kuriki,[a, #] Takafusa Yoshioka,[b,c, #] Mako Kamiya, [*,b] Toru Komatsu,[a] Hiroyuki Takamaru,[d] Kyohhei Fujita,[b] Hirohisa Iwaki,[b] Aika Nanjo,[a] Yuki Akagi,[e] Kohei Takeshita,[b] Haruaki Hino,[b,c] Rumi Hino,[f] Ryosuke Kojima, [b,g] Tasuku Ueno,[a] Kenjiro Hanaoka,[a] Seiichiro Abe,[d] Yutaka Saito,[d] Jun Nakajima,[c] Yasuteru Urano [*,a,b,h]

[a]Graduate School of Pharmaceutical Sciences and [b]Graduate School of Medicine, The University of Tokyo, 7-3-1 Hongo, Bunkyo-ku, Tokyo 113-0033, Japan. [c]Department of Thoracic Surgery, Graduate School of Medicine, The University of Tokyo, 7-3-1 Hongo, Bunkyo-ku, Tokyo 113-8655, Japan. [d]Endoscopy Division, National Cancer Center Hospital. [e]Institute of Engineering, Tokyo University of Agriculture and Technology. [f]Daito Bunka University, Department of Sports and Health Science, 560 Iwadono, Higashimatsuyama, Saitama 355-8501, Japan. [g]PRESTO, Science and Technology Agency (JST), 4-1-8 Honcho Kawaguchi-shi, Saitama 332-0012, Japan. [h]CREST, Agency for Medical Research and Development (AMED), 1-7-1 Otemachi, Chiyoda-ku, Tokyo 100-0004, Japan.

[#] These authors contributed equally to this work.

*Correspondence and requests for materials should be addressed to M.K. and Y.U. (email: mkamiya@m.u-tokyo.ac.jp, uranokun@m.u-tokyo.ac.jp)

Contents

・Supplementary Methods

・Synthesis and characterization of compounds

・Supplementary Figures and Tables

・Supplementary References

Supplementary Methods

Materials and general information

Reagents and solvents were of the best grade available, supplied by Tokyo Chemical Industries, Wako Pure Chemical, Sigma-Aldrich, Dojindo, Kanto Chemical Co., Watanabe Chemical Industries, Merck Millipore and R&D Systems, and were used without further purification. NMR spectra were recorded on a JEOL JNM-LA300 instrument at 300 MHz for ^{1}H NMR and at 75 MHz for ^{13}C NMR, a JEOL JNM-LA400 instrument at 400 MHz for ^{1}H NMR and at 100 MHz for ^{13}C NMR, or a JEOL JNM-ECZ400S instrument at 400 MHz for ^{1}H NMR and at 100 MHz for ^{13}C NMR. All chemical shifts (δ) are reported in ppm relative to internal standard tetramethylsilane (δ = 0.0 ppm), or relative to the signals of residual solvent $CDCl_3$ (7.26 ppm for ^{1}H, 77.16 ppm for ^{13}C), CD_3OD (3.31 ppm for ^{1}H, 49.00 ppm for ^{13}C), CD_2Cl_2 (5.32 ppm for ^{1}H, 49.00 ppm for ^{13}C), acetone-d_6 (2.04 ppm for ^{1}H) or DMSO-d_6 (2.50 ppm for ^{1}H, 39.52 ppm for ^{13}C), and coupling constants are given in Hz. Mass spectra (MS) were measured with a JEOL JMS-T100LC AccuToF (ESI). Preparative HPLC was performed on an Inertsil ODS-3 (10.0 × 250 mm) column (GL Sciences Inc.) using an HPLC system composed of a pump (PU-2080, JASCO) and a detector (MD-2015 or FP-2025, JASCO). Eluent A (H_2O containing 0.1 % TFA), eluent B (80 % acetonitrile and 20 % H_2O containing 0.1 % TFA), eluent E (H_2O containing 100 mM triethylammonium acetate) and eluent F (80 % acetonitrile and 20 % H_2O containing 100 mM triethylammonium acetate) were used for HPLC purification. LC-MS analyses were performed on Inertsil C18 (GL Sciences) fitted on an Agilent Technologies 1200 series/6130 Quadrupole (LC/MS) system, or on Poroshell 120 EC-C18 (Agilent) fitted on LC-MS 2020 (Shimadzu). Eluent C (H_2O containing 0.1 % formic acid) and eluent D (80 % acetonitrile and 20 % H_2O containing 0.1 % formic acid) were used for LC-MS analyses. Gel-permeation chromatography (GPC) purification was performed on a recycle preparative HPLC LC-9110 NEXT (Japan Analytical Industry) equipped with a JAIGEL-2HR column (20 mm × 600 mm, Japan Analytical Industry). Peptides syntheses were performed on an automatic peptide synthesizer (Syro I; Biotage).

Collection of clinical samples of lung tissues

All specimens, including lung tumour and normal lung tissues, were obtained from the Department of Thoracic Surgery, Graduate School of Medicine, University of Tokyo. Before the study, all the patients provided written informed consent for this ex-vivo lung cancer fluorescence imaging study. The Research Review Board at our institution examined and approved the research protocol, which was in accordance with the Declaration of Helsinki. Histologic tumour type was assessed according to the fourth edition of the World Health Organization classification. After the imaging experiment, specimens were preserved in 10 % formalin. Unused specimens were stored at -80 ˚C.

Preparation of lung tissue lysate

To prepare the lysate, freeze-dried tissues were put in a homogenizer and 1 mL of tissue protein extraction reagent was added. The tissues were homogenized and the homogenate was centrifuged in 1.5 mL plastic tubes (15,000

rpm x 10 min at 4 ˚C). The supernatant was collected as tissue lysate, and the protein concentration was determined with a standard BCA assay. The lysate were aliquoted and kept -80 ˚C.

Screening with tissue lysates

Stock solutions of the probes or HMRG (200 μM) were dissolved in phosphate-buffered saline (pH 7.4) containing 100 mg/L $CaCl_2$ and $MgCl_2 \cdot 6H_2O$ to make 1.33 μM probe solutions, and 15 μL aliquots were dispensed into the wells of half-area 384-well plates (Corning 3677). Then, tumour or non-tumour tissue lysates (0.5 mg/mL, 5 μL) were dispensed into the wells (final concentration of probe or HMRG: 1 μM, lysate: 0.1 mg/mL), and the initial fluorescence intensity was measured three times at 1 min intervals (λ_{ex} = 485 nm, λ_{em} = 535 nm) with a microplate reader (EnVision 2103 Multilabel Reader (PerkinElmer). After incubation at 37 °C for 60 min, the fluorescence intensity was again measured three times at 1 min intervals. Fluorescence intensity at 0 min or 60 min was calculated as an average of the three measurements. Conversion rate was calculated as follows;

$$\text{Conversion rate (\%)} = \frac{(\text{F. I. of probe at 60 min} - \text{F. I. of probe at 0 min})}{\text{F. I. of HMRG at 60 min}} \times 100$$

Fluorescence imaging with lung resected specimens

All fluorescence imaging was performed within 1–2 h after lung resection on a Maestro® in-vivo imaging system (Perkin Elmer), before and at 5, 10, and 30 min after applying approximately 200 μL of 50 μM probe solution in PBS (-) to lung tumour and normal lung specimens at room temperature. The excitation and emission wavelengths were 445–490 nm and 515 nm long-pass, respectively. To evaluate fluorescence intensity, regions of interest (ROIs) were drawn for both lung tumour and normal lung and the fluorescence intensity was calculated using Maestro® software. Fluorescence increase was defined by subtracting initial fluorescence intensity from that measured after 30 min of incubation with the probe. As a PSA inhibitor, 500 μM puromycin was added.

Diced electrophoresis gel assay

The assay was performed using a specialized instrument for DEG assay (http://www.sainome.jp/index_e.html). After 2D electrophoresis of lysate under non-denatured conditions[1], the gels were put on the plate, the lid was put on the gel to dice it, and centrifugation was done at 3,000 rpm × 10 min. Assays were performed by adding 80 μL of KK-HMRG or KH-HMRG solution (1 μM) to each well, and the initial fluorescence intensity was measured three times at 1 min intervals with a microplate reader (EnVision 2103 Multilabel Reader (PerkinElmer). Excitation/emission wavelengths were 485 nm/535 nm. After incubation at 37 °C overnight, fluorescence intensity was again measured three times at 1 min intervals. The fluoresecence increase rates were summarized in the form of a heat map.

Peptide mass fingerprinting

LS-MS/MS-based PMF analysis was performed as a contract service by APRO Life Science Institute, Inc. The gel pieces in the wells showing the desired activities were washed three times with H_2O, and kept at -80°C before the

analysis. Peptide samples were prepared by reductive alkylation and trypsin digestion according to standard protocols. Peptides were separated on a Paradigm MS2 (Michrom BioResources, Inc.) equipped with an L-column ODS (0.1 × 50 mm, Chemicals Evaluation and Research Institute) under an acidic solvent condition (0.1 % formic acid) with an increasing gradient of acetonitrile. Detection was done with a Q-Tof2 (Waters Micromass) in the positive mode (capillary voltage: 1.8 kV, collision energy: 20-56 eV).

Analysis of LC-MS/MS data

The acquired LC-MS/MS data was analyzed using MASCOT Server 2.3 (Matrix Science Ltd.) to find hit proteins. The threshold was set at $P < 0.05$. The database to be searched was that for *Homo sapiens*. The list of hit proteins may contain multiple proteins in addition to the true target, so available information on all hit proteins in the literature was scanned in protein and enzyme databases such as UniProt (http://www.uniprot.org/) and BRENDA (http://www.brendaenzymes.org/) in order to identify proteins likely to accept the peptides as substrates.

Enzyme assay with KK-HMRG

1 μM KK-HMRG in phosphate-buffered saline (pH 7.4) with 100 mg/L $CaCl_2$ and $MgCl_2 \cdot 6H_2O$ containing DMSO as a cosolvent was reacted with 500 ng tissue lysate or 2.5 ng PSA (6410-ZN-010, R&D Systems) in the presence or absence of inhibitor (PSA, 3,4-DCI, SNJ1945 or SC-57461A) at 37 °C (n = 4). The total assay volume was 20 μL and the fluorescence increase was measured with a plate reader. Excitation/emission wavelengths were 485 nm/535 nm.

Western blotting

After SDS-PAGE of tissue lysates from 5 lung adenocarcinoma patients (10 μg of protein was loaded), western blotting was performed under the following conditions. The first antibodies were directed to human PSA (Santa Cruz, sc-390184, 1/500 dilution) or human β-actin (Santa Cruz, sc-47778, 1/1000 dilution), and incubation was done at room temperature for 2 h. The second antibody was HRP-linked anti mouse IgG (GE Healthcare NA931V, 1/5000 dilution), and incubation was done at room temperature for 1 h. Chemiluminescence reaction was performed with the use of a WESTAR Supernova (Cyanagen). Detection was done with an ImageQuant CAS 4000 mini (GE Healthcare).

Collection of ESD samples of gastric cancer

Between January 2015 and March 2019, 54 ESDs in the National Cancer Center Hospital were included in this study. Eligibility for ESD was assessed by endoscopic examination. Indication criteria of the ESD were as follows; 1) gastric cancer proved by biopsy and 2) diagnosed clinical depth T1a according to the Japanese gastric cancer treatment guidelines[2, 3]. This study was conducted in accordance with the principles outlined in the Declaration of Helsinki and was approved by the institutional review board of the National Cancer Center Hospital (IRB number: 2014-370). The informed consent of all patients was included as a part of the comprehensive written consents required by our institution. ESD samples used for inhibitor assay were stored at -80 ˚C until used for fluorescence imaging.

Ex vivo screening on ESD samples of gastric cancer with Tetra-PEG gel or medical gauze

Tetra-PEG gel was prepared as reported previously[4]. Small pieces of Tetra-PEG gel were soaked in 50 μM probe solution in RPMI (phenol red (-)) for more than 30 min. Just before the start of imaging, presoaked gels were taken from the solution and placed on both tumour and non-tumour regions of ESD samples. In the case of medical gauze, small pieces of medical gauze were put on both tumour and non-tumour regions of the specimens, and approximately 10 μL of 50 μM probe solution was locally dropped on the gauze. Fluorescence images were captured with a Discovery imaging system (INDEC Inc., Santa Clara, Calif., USA) before and at 1, 3, 5, 10, 15, 20 and 30 min after addition of probes. All fluorescence imaging was performed within 1–2 h after resection. Fluorescence intensity was calculated with ImageJ.

Ex vivo imaging of ESD samples of gastric cancer with KH-HMRG

Fluorescence images were captured with a Discovery imaging system (INDEC Inc., Santa Clara, Calif., USA) before and at 1, 3, 5, 10, 15, 20 and 30 min after spraying of 50 μM KH-HMRG in RPMI (phenol red (-)) onto the samples. All fluorescence imaging was performed within 1–2 h after resection.

Inhibitory effect of bestatin on ex vivo imaging of ESD samples of gastric normal region with KH-HMRG

ESD samples were preincubated in the absence or presence of 100 μM bestatin in DPBS(-) for 10 min before addition of an equal volume of 100 μM KH-HMRG. Fluorescence images were captured with a Maestro® in-vivo imaging system (Perkin Elmer) at 0, 1, 3, 5, 10, 15, 20 and 30 min after addition of KH-HMRG to normal regions of ESD gastric samples. The excitation and emission wavelengths were 445–490 nm and 515 nm long-pass, respectively.

Preparation of cell lysate of human cancer cell lines.

RAW246.7, HEK293, PC12, dPC12, SKOV2, NHBE, HT29, Jurkat, U2OS, H226, NIH3T3, MCF7, HUVEC, OVCAR3, A549, HepG2, HL60 and dHL60 cells were cultured in 10 cm dishes with optimized media. Differentiated HL60 cells were prepared according to the literature[5]. Cells were cultured in RPMI1640 containing 20% FBS and 1.35% DMSO, and cultured for 4-5 days for differentiation. When the cells reached 50-80 % confluency, they were washed with PBS twice, and lysed by addition of 1 mL CelLyticM to the plate, followed by incubation at room temperature for 10 min. The solution was collected and centrifuged (14000 rpm × 5 min at 4˚C). The supernatant was collected, aliquoted, and stored at -80˚C. Protein concentration was determined with the standard Bradford assay.

Enzyme assay with KH-HMRG

1 μM KH-HMRG in phosphate-buffered saline (pH 7.4) containing DMSO as a cosolvent was reacted with culture cell lysate or APN (3815-ZN-010, R&D Systems) in the presence or absence of bestatin at 37 °C (n = 4). The total assay volume was 20 μL and the fluorescence increase was measured with a plate reader. Excitation/emission wavelengths were 485 nm/535 nm.

Immunocytostaining of APN

After fixation of HT1080 or HEK293 cells with 4 % PFA in PBS followed by blocking with 1 % BSA in PBS, immunocytostaining was performed under the following conditions. The first antibody was directed to mouse CD13 (APN) (abcam, 7417, 1/100 dilution), but has similar reactivity with human CD13, and incubation was done at room temperature for 1 h. The second antibody was anti-mouse IgG H&L (Alexa Fluor® 488) (abcam, 150105, 1/500), and incubation was done at room temperature for 1 h. After additon of mouting solution with DAPI, fluorescence imaging was performed with a Leica Application Suite Advanced Fluorescence (LAS-AF) instrument with a TCS SP5.

Fluorescence confocal microscopy

Fluorescence images were captured using a Leica Application Suite Advanced Fluorescence (LAS-AF) instrument with a TCS SP5. Living cells were washed twice with HBSS and incubated at 37 °C with KH-HMRG or A-HMRG (100 nM) for 30 min. After incubation, differential interference contrast and fluorescence images were captured.

Histological analysis

Excised specimens were immediately fixed with 10 % formaldehyde for at least 48 h. Formalin-fixed paraffin-embedded tissues were sectioned at 4 μm thickness and stained with hematoxylin and eosin for histopathological evaluation. Experienced pathologists examined each sample in a blind manner, and dysplasia and neoplasia were diagnosed.

Immunohistochemical analysis of APN expression

Sections were deparaffinized in Histo-Clear, sequentially washed in 100 %, 90 %, 80 % and 70 % ethanol, and then washed in PBS. After heat-induced antigen retrieval (citrate buffer, pH 6) using a microwave device, each slide was pre-incubated in 3 % H_2O_2 for 20 min, reacted with primary antibodies (rabbit polyclonal antibody; Product number: HPA004625, Lot: C118611 and 000018631, Sigma-Aldrich) in 5 % skim milk for 90 min, and secondary antibodies (TaKaRa POD Conjugate Anti Rabbit, For Tissue, Product number: MK205, Lot: AJ92256A, TaKaRa) for 30 min at room temperature. Each slide was visualized with a 3,3'-diaminobenzidine tetrahydrochloride (DAB) detection kit (Product number: MK210, TaKaRa), and counter-stained with hematoxylin. APN antibody was diluted to 1/10.

Comparison of kinetic parameters

DMSO solutions of KK-HMRG, KH-HMRG, KK-AMC and KH-AMC were diluted in 20 μL of PBS (-) containing 500 μM Triton X-100 to obtain various probe concentrations, and the solutions were added to the wells of 384-well plates. Then, PSA (final concentration: 0.01 μM for KK-HMRG and KK-AMC) or APN (final concentration: 0.01 μM for KH-HMRG, 0.05 μM for KH-AMC) was added and the plates were incubated at 37 °C. The fluorescence increase was measured with a plate reader (n = 4). Excitation/emission wavelengths were 485 nm/535 nm for HMRG-based probes and 355 nm/460 nm for AMC-based probes. Initial reaction velocity was plotted against probe concentration, and fitted to the Michaelis-Menten equation.

Synthesis and characterization of compounds

Compounds 2, 3

Compounds 2 and 3 were synthesized according to the literature[6].

General procedure for synthesis of compound 4a – 4y

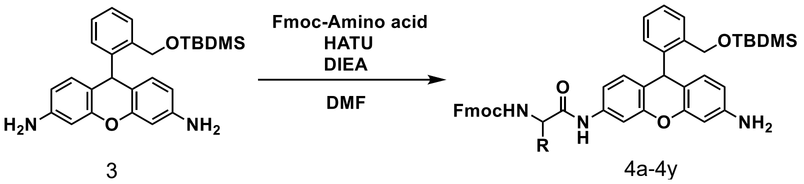

3 4a-4y

To the solution of compound **3** (1.5-2 eq), Fmoc-amino acid (1 eq) and HATU (1 eq) in DMF was added DIEA (2 eq). The mixture was stirred at 50 °C under an Ar atmosphere for 1.5 h, then cooled to room temperature, and AcOEt was added. The organic solution was washed with brine or sat.NH_4Cl aq. many times, dried over with Na_2SO_4 and evaporated to dryness. The residue was purified by column chromatography over silica gel. If any impurity was detected, additional purification was performed with a GPC recycle column using chloroform as the eluent.

Compound 4a (Fmoc-Gly TBDMS leuco HMRG)

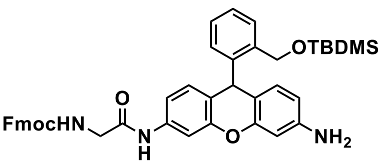

Compound **4a** was synthesized from compound **3** (104 mg, 0,241 mmol), Fmoc-Gly-OH (36 mg, 0.121 mmol), HATU (46 mg, 0.121 mmol) and DIEA (43 μL, 0.243 mmol) following the general procedure. After purification by column chromatography over silica gel using AcOEt/*n*-hexane (1/1) as the eluent, compound **4a** was obtained (48 mg, 56 %) as a slightly orange powder. ^{1}H NMR (300 MHz, CD_2Cl_2): δ 0.03 (s, 6H), 0.89 (s, 9H), 3.73 (s, 2H), 3.99 (d, J = 4.4 Hz, 2H), 4.23 (t, J = 6.6 Hz, 1H), 4.43 (d, J = 7.3 Hz, 2H), 4.69 (d, J = 13.2 Hz, 1H), 4.74 (d, J = 13.2 Hz, 1H), 5.45 (s, 1H), 5.88 (s, 1H), 6.26 (dd, J = 2.2, 8.1 Hz, 1H), 6.35 (d, J = 1.5 Hz, 1H), 6.68 (d, J = 8.1 Hz, 1H), 6.82 (d, J = 8.1 Hz, 1H), 6.90 (d, J = 8.1 Hz, 1H), 7.00-7.11 (m, 1H), 7.11-7.21 (m, 2H), 7.21-7.32 (m, 2H), 7.32-7.50 (m, 4H), 7.60 (d, J = 7.3 Hz, 2H), 7.75 (d, J = 7.3 Hz, 2H), 8.34 (s, 1H); ^{13}C NMR (100 MHz, $CDCl_3$): δ -5.34, 18.3, 25.9, 39.1, 45.3, 47.0, 63.1, 67.4, 102.2, 107.7, 110.9, 113.7, 114.5, 120.0, 120.4, 125.0, 126.6, 127.1, 127.5, 127.6, 127.7, 130.1, 130.3, 130.8, 136.9, 138.4, 141.2, 143.6, 146.2, 150.9, 151.1, 157.0, 167.1**;** HRMS (ESI$^+$): calcd for $[M+H]^+$, 712.32067 ; found, 712.31653 (-4.14 mmu)

Compound 4b (Fmoc-Glu(O*t*Bu) TBDMS leuco HMRG)

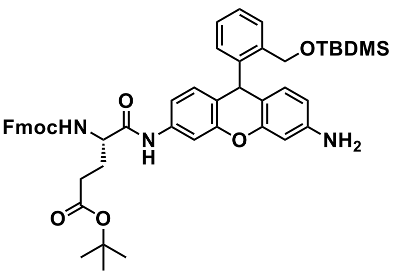

Compound **4b** was synthesized from compound **3** (112 mg, 0.260 mmol), Fmoc-Glu(O*t*Bu)-OH (58 mg, 0.131 mmol), HATU (50 mg, 0.132 mmol) and DIEA (46 μL, 0.260 mmol) following the general procedure. After purification by column chromatography over silica gel using AcOEt/*n*-hexane (1/1) as the eluent and GPC recycle column chromatography using chloroform

as the eluent, compound **4b** was obtained (diastereomer mixture, 50 mg, 45 %,) as a slightly orange powder. ^{1}H NMR (400 MHz, CD_2Cl_2): δ 0.04 (s, 6H), 0.89 (s, 9H), 1.45 (s, 9H), 1.90-2.03 (m, 1H), 2.05-2.19 (m, 1H), 2.29-2.42 (m, 1H), 2.42-2.56 (m, 1H), 4.17-4.31 (m, 2H), 4.37-4.47 (m, 2H), 4.71 (d, *J* = 12.2 Hz, 1H), 4.79 (d, *J* = 12.2 Hz, 1H), 5.49 (s, 1H), 5.81 (s, 1H), 6.30 (d, *J* = 8.3 Hz, 1H), 6.43 (s, 1H), 6.70 (d, *J* = 8.3 Hz, 1H), 6.83-6.98 (m, 2H), 7.06 (s, 1H), 7.14-7.24 (m, 2H), 7.24-7.35 (m, 2H), 7.35-7.49 (m, 4H), 7.61 (d, *J* = 6.3 Hz, 2H), 7.77 (d, *J* = 7.8 Hz, 2H), 8.32 (s, 1H)**;** ^{13}C NMR (100 MHz, $CDCl_3$): δ -5.42, 18.3, 25.9, 28.0, 28.2, 31.8, 39.0, 47.0, 54.8, 63.1, 67.2, 81.3, 102.1, 107.6, 107.7, 110.9, 113.8, 114.5 (containing 2 peaks), 119.9, 120.4, 125.0 (containing 2 peaks), 126.5, 127.1, 127.5, 127.7, 130.0, 130.1, 130.3, 130.8, 136.9, 138.3, 141.2, 143.6, 143.7, 143.8, 146.2, 150.9, 151.2, 156.5, 169.4, 173.1**;** HRMS (ESI$^+$): calcd for $[M+H]^+$, 840.40440 ; found, 840.40694 (2.54 mmu)

Compound 4c (Fmoc-Lys (Boc) TBDMS leuco HMRG)

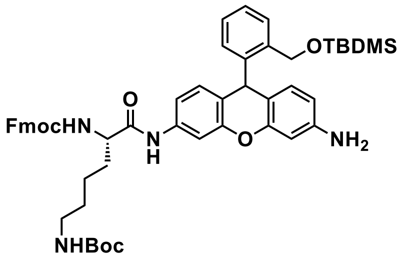

Compound **4c** was synthesized from compound **3** (309 mg, 0.715 mmol), Fmoc-Lys (Boc)-OH (173 mg, 0.370 mmol), HATU (137 mg, 0.360 mmol) and DIEA (128 μL, 0.722 mmol) following the general procedure. After purification by column chromatography over silica gel using AcOEt/*n*-hexane (1/1) as the eluent and GPC recycle column chromatography using chloroform as the eluent, compound **4c** was obtained (diastereomer mixture, 142 mg, 43 %) as a slightly orange powder. ^{1}H NMR (300 MHz, CD_2Cl_2): δ 0.04 (s, 6H), 0.88 (s, 9H), 1.41 (s, 9H), 1.44-1.57 (m, 4H), 1.60-1.78 (m, 1H), 1.86-2.10 (m, 1H), 2.93-3.24 (m, 2H), 3.72 (s, 2H), 4.11-4.28 (m, 2H), 4.35-4.51 (m, 2H), 4.56-4.89 (m, 3H), 5.46 (s, 1H), 5.55 (s, 1H), 6.27 (dd, *J* = 8.1, 2.2 Hz, 1H), 6.40 (d, *J* = 2.2 Hz, 1H), 6.69 (d, *J* = 8.8 Hz, 1H), 6.86 (d, *J* = 8.1 Hz, 1H), 6.93 (dd, *J* = 8.1, 1.5 Hz, 1H), 7.01-7.09 (m, 1H), 7.11-7.23 (m, 2H), 7.23-7.33 (m, 2H), 7.34-7.51 (m, 4H), 7.61 (d, *J* = 7.3 Hz, 2H), 7.77 (d, *J* = 8.1 Hz, 2H), 8.15 (s, 1H)**;** ^{13}C NMR (75 MHz, $CDCl_3$): δ -5.45, 18.3, 22.4, 25.9, 28.4, 29.4, 31.9, 38.8, 39.6, 46.9, 55.4, 63.1, 67.1, 79.1, 102.1, 107.6, 110.8, 113.7, 114.4, 114.5, 119.8, 120.2, 120.3, 124.9, 126.4, 127.0, 127.4, 127.6, 130.0, 130.3, 130.7, 137.1, 138.2, 141.1, 143.5, 143.6, 146.1, 150.8, 151.1, 156.2, 156.5, 170.3**;** HRMS (ESI$^+$): calcd for $[M+Na]^+$, 905.42854 ; found, 905.42506 (-3.48 mmu)

Compound 4d (Fmoc-Tyr (*t*Bu) TBDMS leuco HMRG)

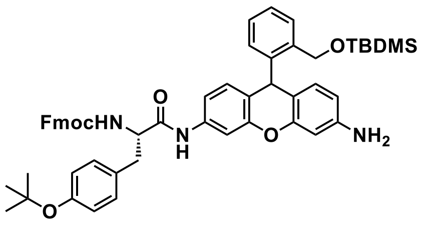

Compound **4d** was synthesized from compound **3** (391 mg, 0.905 mmol), Fmoc-Tyr (*t*Bu)-OH (210 mg, 0.458 mmol), HATU (174 mg, 0.458 mmol) and DIEA (162 μL, 0.914 mmol) following the general procedure. After purification by column chromatography over silica gel using AcOEt/*n*-hexane (1/1) as the eluent and GPC recycle column chromatography using chloroform as the eluent, compound **4d** was obtained (diastereomer mixture, 195 mg, 49 %) as a slightly orange powder. ^{1}H NMR (300 MHz, CD_2Cl_2): δ 0.05 (s, 6H), 0.90 (s, 9H), 1.17-1.35 (m, 9H), 3.08 (d, *J* = 4.4 Hz, 2H), 3.72 (s, 2H), 4.21 (t, *J* = 6.6 Hz, 1H), 4.30-4.47 (m, 2H), 4.49 (s, 1H), 4.63-4.89 (m, 2H),

5.40-5.53 (m, 1H), 5.66 (s, 1H), 6.28 (dd, J = 2.2, 8.1 Hz, 1H), 6.38 (d, J = 2.2 Hz, 1H), 6.69 (d, J = 8.1 Hz, 1H), 6.72-6.85 (m, 2H), 6.89 (d, J = 8.1 Hz, 2H), 7.00-7.49 (m, 11H), 7.51-7.65 (m, 2H), 7.77 (d, J = 7.3 Hz, 2H), 7.82 (s, 1H); ^{13}C NMR (100 MHz, $CDCl_3$): δ -5.42, 18.3, 25.9, 28.8, 38.2, 39.0 (containing 2 peaks), 47.0, 57.1, 63.1 (containing 2 peaks), 67.2, 78.4, 102.1, 107.8, 110.9, 113.7 (containing 2 peaks), 114.6, 114.7, 119.9, 120.4, 120.6, 124.3, 125.0, 126.5, 127.1, 127.4, 127.5, 127.6, 127.7, 129.8, 129.9, 130.0, 130.3, 130.8 (containing 2 peaks), 130.9, 136.5, 138.3, 138.4, 141.2, 143.5, 143.6, 146.2, 150.8 (containing 2 peaks), 151.1, 154.5, 156.2, 169.2**;** HRMS (ESI$^+$): calcd for [M+H]$^+$, 874.42514 ; found, 874.42178 (-3.36 mmu)

Compound 4e (Fmoc-Leu TBDMS leuco HMRG)

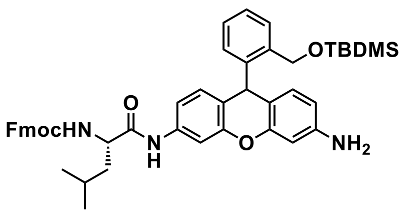

Compound **4e** was synthesized from compound **3** (306 mg, 0.708 mmol), Fmoc-Leu-OH (125 mg, 0.354 mmol), HATU (135 mg, 0.360 mmol) and DIEA (126 μL, 0.711 mmol) following the general procedure. After purification by column chromatography over silica gel using AcOEt/*n*-hexane (1/1) as the eluent, compound **4e** was obtained (diastereomer mixture, 189 mg, 70 %) as a slightly orange powder. ^{1}H NMR (300 MHz, CD_2Cl_2): δ 0.04 (s, 6H), 0.89 (s, 9H), 0.94-0.95 (m, 6H), 1.60-1.65 (m, 3H), 3.81 (br s, 2H), 4.20 (br s, 1H), 4.23 (t, 1H, J = 6.6 Hz), 4.47 (d, 2H, J = 6.6 Hz), 4.70 (d, 1H, J = 12.5 Hz), 4.77 (d, 1H, J = 12.5 Hz), 5.21 (br s, 1H), 5.48 (s, 1H), 6.28 (dd, 1H, J = 2.0, 8.1 Hz), 6.40 (d, 1H, J = 2.0 Hz), 6.69 (d, 1H, J = 8.1 Hz), 6.85-6.88 (m, 2H), 7.04-7.06 (m, 1H), 7.14-7.21 (m, 2H), 7.29-7.31 (m, 2H), 7.36-7.43 (m, 4H), 7.59 (d, 2H, J = 7.3 Hz), 7.77 (d, 2H, J = 7.3 Hz), 7.92 (br s, 1H); ^{13}C NMR (100 MHz, $CDCl_3$): δ -5.43, -5.41, 18.3, 22.8, 24.7, 25.9, 38.9, 39.1, 41.1, 47.1, 54.4, 63.1, 63.2, 67.2, 102.2 (containing 2 peaks), 107.7 (containing 2 peaks), 110.8, 113.7, 113.8, 114.5, 114.6, 119.9, 120.2, 120.4, 124.9 (containing 2 peaks), 126.5, 127.1, 127.4, 127.5, 127.6, 127.7, 130.0 (containing 2 peaks), 130.3, 130.7, 130.8, 137.0, 138.3 (containing 2 peaks), 141.2, 143.5, 143.6, 143.8, 146.2, 150.8, 150.9, 151.2, 156.7, 170.6 (containing 2 peaks); HRMS (ESI$^+$): calcd for [M+Na]$^+$, 790.36522 ; found, 790.36749 (2.27 mmu)

Compound 4f (Fmoc-Pro TBDMS leuco HMRG)

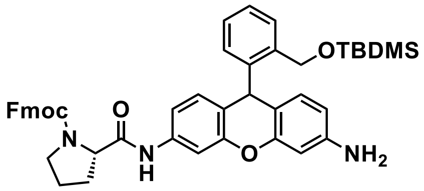

Compound **4f** was synthesized from compound **3** (262 mg, 0.606 mmol), Fmoc-Pro-OH (114 mg, 0.321 mmol), HATU (121 mg, 0.318 mmol) and DIEA (108 μL, 0.609 mmol) following the general procedure. After purification by column chromatography over silica gel using AcOEt/*n*-hexane (1/1) as the eluent and GPC recycle column chromatography using chloroform as the eluent, compound **4f** was obtained (diastereomer mixture, 96 mg, 40 %) as a slightly orange powder. ^{1}H NMR (300 MHz, CD_2Cl_2): δ 0.04 (s, 6H), 0.89 (s, 9H), 1.95 (br s, 4H), 3.51 (br s, 2H), 3.72 (br s, 2H), 4.26 (br s, 1H), 4.41-4.44 (m, 3H), 4.70 (d, 1H, J = 13.2 Hz), 4.78 (d, 1H, J = 12.5 Hz), 5.48 (s, 1H), 6.27 (dd, 1H, J = 2.2, 8.1 Hz), 6.40 (d, 1H, J = 2.2 Hz), 6.69 (d, 1H, J = 8.1 Hz), 6.86-6.89 (m, 2H), 7.06 (br s, 1H), 7.16-7.42 (m, 8H), 7.60 (br s, 2H), 7.76 (br s, 2H), 8.99 (br s, 1H); HRMS (ESI$^+$): calcd for [M+H]$^+$, 752.35197 ;

found, 752.35316 (1.19 mmu)

Compound 4g (Fmoc-D-Ala TBDMS leuco HMRG)

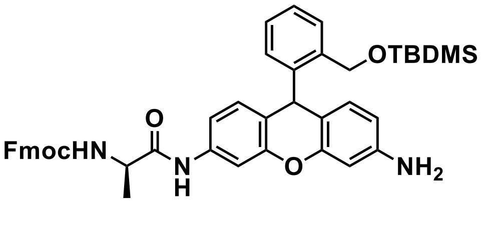

Compound **4g** was synthesized from compound **3** (355 mg, 0.822 mmol), Fmoc-D-Ala-OH (138 mg, 0.422 mmol), HATU (160 mg, 0.421 mmol) and DIEA (146 µL, 0.824 mmol) following the general procedure. After purification by column chromatography over silica gel using AcOEt/*n*-hexane (1/1) as the eluent and GPC recycle column chromatography using chloroform as the eluent, compound **4g** was obtained (diastereomer mixture, 189 mg, 70 %) as a slightly orange powder. ^{1}H NMR (300 MHz, CD_2Cl_2): δ 0.04 (s, 6H), 0.89 (s, 9H), 1.42 (d, 3H, *J* = 6.6 Hz), 3.73 (br s, 2H), 4.23 (t, *J* = 6.6 Hz , 1H), 4.28 (br s, 1H), 4.46 (d, 2H, *J* = 6.6 Hz), 4.70 (d, 1H, *J* = 12.6 Hz), 4.76 (d, 1H, *J* = 12.6 Hz), 5.49 (s, 1H), 6.28 (dd, 1H, *J* = 2.2, 8.1 Hz), 6.41 (d, 1H, *J* = 2.2 Hz), 6.69 (d, 1H, *J* = 8.1 Hz), 6.87-6.90 (m, 2H), 7.05-7.06 (m, 1H), 7.15-7.21 (m, 2H), 7.29-7.31 (m, 2H), 7.37-7.43 (m, 4H), 7.60 (d, 2H, *J* = 7.3 Hz), 7.77 (d, 2H, *J* = 7.3 Hz), 8.00 (br s, 1H); ^{13}C NMR (75 MHz, $CDCl_3$): δ -5.39, 18.3, 18.6, 25.9, 39.0, 47.0, 51.2, 63.1, 67.2, 102.2, 107.7, 110.9, 113.8, 114.5, 119.9, 120.3, 124.9, 125.0, 126.5, 127.1, 127.4, 127.7, 130.0, 130.3, 130.8, 137.0, 138.3, 141.2, 143.4, 143.5, 146.1, 150.8, 151.1, 156.3, 170.5; HRMS (ESI$^+$): calcd for $[M+H]^+$, 726.33632 ; found, 726.33301 (-3.31 mmu)

Compound 4h (Fmoc-D-Asp(*t*Bu) TBDMS leuco HMRG)

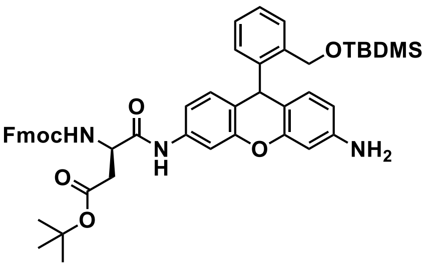

Compound **4h** was synthesized from compound **3** (492 mg, 1.14 mmol), Fmoc-D-Asp(*t*Bu)-OH (238 mg, 0.578 mmol), HATU (226 mg, 0.594 mmol) and DIEA (202 µL, 1.14 mmol) following the general procedure. After purification by column chromatography over silica gel using AcOEt/*n*-hexane (1/1) as the eluent and GPC recycle column chromatography using chloroform as the eluent, compound **4h** was obtained (diastereomer mixture, 277 mg, 58 %) as a slightly orange powder. ^{1}H NMR (300 MHz, CD_2Cl_2): δ 0.04 (s, 6H), 0.89 (s, 9H), 1.40-1.48 (m, 9H), 2.67 (dd, 1H, *J* = 17.0, 5.3 Hz), 2.90 (dd, 1H, *J* = 17.0, 5.3 Hz), 4.08 (br s, 2H), 4.25 (t, 1H, *J* = 6.6 Hz), 4.46 (d, 2H, *J* = 6.6 Hz), 4.59 (m, 1H), 4.69 (d, 1H, *J* = 12.9 Hz), 4.76 (d, 1H, *J* = 12.9 Hz), 5.49 (s, 1H), 5.97 (d, 1H, *J* = 7.3 Hz), 6.30 (dd, 1H, *J* = 2.2, 8.1 Hz), 6.44 (d, 1H, *J* = 2.2 Hz), 6.70 (d, 1H, *J* = 8.1 Hz), 6.87-6.91 (m, 2H), 7.05-7.07 (m, 1H), 7.15-7.21 (m, 2H), 7.29-7.31 (m, 2H), 7.39-7.41 (m, 4H), 7.61 (d, 2H, *J* = 7.3 Hz), 7.78 (d, 2H, *J* = 7.3 Hz), 8.41 (br s, 1H); ^{13}C NMR (100 MHz, $CDCl_3$) δ -5.47, 18.3, 25.9, 27.9, 37.5, 39.0, 47.0, 51.7, 63.1, 67.1, 82.0, 102.0, 107.6, 110.8, 113.6, 114.4, 119.9, 120.3, 124.9, 126.5, 127.0, 127.4, 127.6, 127.7, 130.0 (containing 2 peaks), 130.3, 130,7, 136.7, 138.2, 141.2, 143.5, 143.7, 146.2, 150.8, 151.1, 156.2, 168.4, 171.1; HRMS (ESI$^+$): calcd for $[M+H]^+$, 826.38875 ; found, 826.39082 (2.07 mmu)

Compound 4i (Fmoc-D-Ser(*t*Bu) TBDMS leuco HMRG)

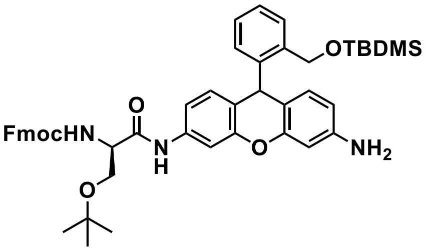

Compound **4i** was synthesized from compound **3** (447 mg, 1.03 mmol), Fmoc-D-Ser(*t*Bu)-OH (205 mg, 0.535 mmol), HATU (201 mg, 0.529 mmol) and DIEA (183 μL, 1.03 mmol) following the general procedure. After purification by column chromatography over silica gel using AcOEt/*n*-hexane (1/1) as the eluent and GPC recycle column chromatography using chloroform as the eluent, compound **4i** was obtained (diastereomer mixture, 277 mg, 58 %) as a slightly orange powder. ^{1}H NMR (300 MHz, CD_2Cl_2): δ 0.04 (s, 6H), 0.89 (s, 9H), 1.23 (s, 9H), 3.43-3.48 (m, 1H), 3.84-3.85 (m, 1H), 4.07 (br s, 2H), 4.25 (t, 1H, *J* = 6.6 Hz), 4.29 (br s, 1H), 4.43 (d, 2H, *J* = 6.6 Hz), 4.69 (d, 1H, *J* = 12.5 Hz), 4.74 (d, 1H, *J* = 13.2 Hz), 5.49 (s, 1H), 5.78 (br s,1H), 6.30 (dd, 1H, *J* = 2.2, 8.1 Hz), 6.44 (d, 1H, *J* = 2.2 Hz), 6.70 (d, 1H, *J* = 8.1 Hz), 6.88-6.92 (m, 2H), 7.06-7.08 (m, 1H), 7.16-7.22 (m, 2H), 7.31-7.33 (m, 2H), 7.40-7.43 (m, 4H), 7.63 (d, 2H, *J* = 6.6 Hz), 7.79 (d, 2H, *J* = 7.3 Hz), 8.59 (br s, 1H); ^{13}C NMR (75 MHz, $CDCl_3$): δ -5.51, 18.3, 25.8, 27.3, 39.1, 47.0, 54.6, 61.8, 63.0, 67.0, 74.5, 102.0, 107.3, 110.8, 113.4, 114.2, 119.8, 120.1, 125.0, 126.5, 126.9, 127.4, 127.5, 127.6, 130.0, 130.1, 130.2, 130.7, 136.8, 138.3, 141.1, 143.5, 143.6, 146.2, 150.8, 151.0, 156.0, 168.2; HRMS (ESI^{+}): calcd for $[M+H]^{+}$, 798.39384 ; found, 798.39356 (-0.28 mmu)

Compound 4j (Fmoc-βAla TBDMS leuco HMRG)

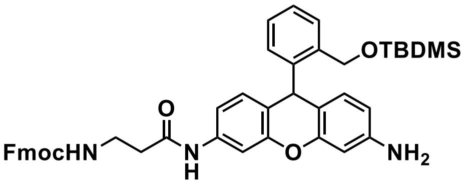

Compound **4j** was synthesized from compound **3** (499 mg, 1.16 mmol), Fmoc-β-Ala-OH (181 mg, 0.582 mmol), HATU (225 mg, 0.592 mmol) and DIEA (206 μL, 1.16 mmol) following the general procedure. After purification by column chromatography over silica gel using AcOEt/*n*-hexane (2/1) as the eluent and GPC recycle column chromatography using chloroform as the eluent, compound **4j** was obtained (222 mg, 53 %) as a slightly orange powder. ^{1}H NMR (300 MHz, CD_2Cl_2): δ 0.04 (s, 6H), 0.89 (s, 9H), 2.45-2.70 (m, 2H), 3.52 (d, *J* = 5.1 Hz, 2H), 3.72 (s, 2H), 4.21 (t, *J* = 7.0 Hz, 1H), 4.36 (d, *J* = 6.6 Hz, 2H), 4.70 (d, *J* = 12.5 Hz, 1H), , 4.76 (d, *J* = 13.2 Hz, 1H), 5.44 (s, 1H), 5.48 (s, 1H), 6.28 (dd, *J* = 2.6, 8.4 Hz, 1H), 6.40 (d, *J* = 2.2 Hz, 1H), 6.69 (d, *J* = 8.8 Hz, 1H), 6.83-6.95 (m, 2H), 7.03-7.10 (m, 1H), 7.14-7.22 (m, 2H), 7.23-7.33 (m, 2H), 7.34-7.48 (m, 5H), 7.59 (d, *J* = 7.3 Hz, 2H), 7.76 (d, *J* = 7.3 Hz, 2H); ^{13}C NMR (75 MHz, $CDCl_3$): δ -5.45, 18.3, 25.9, 36.9, 38.8, 47.0, 63.1, 66.7, 102.1, 107.6, 110.8, 113.6, 114.4, 119.8, 120.2, 124.9, 126.4, 127.0, 127.4, 127.6 (containing 2 peaks), 130.0, 130.3, 130.7, 137.1, 138.2, 141.1, 143.6, 146.2, 150.8, 151.1, 156.7, 169.6; HRMS (ESI^{+}): calcd for $[M+H]^{+}$, 726.33632 ; found, 726.33691 (0.59 mmu)

Compound 4k (Fmoc-MeGly TBDMS leuco HMRG)

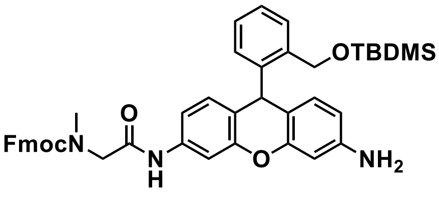

Compound **4k** was synthesized from compound **3** (507 mg, 1.17 mmol), Fmoc-Sar-OH (190 mg, 0.611 mmol), HATU (222 mg, 0.584 mmol) and DIEA (207 μL, 1.17 mmol) following the general procedure. After purification by column chromatography over silica gel using

AcOEt/*n*-hexane (1/1) as the eluent and GPC recycle column chromatography using chloroform as the eluent, compound **4k** was obtained (222 mg, 58 %) as a slightly orange powder. ^{1}H NMR (300 MHz, CD_2Cl_2): δ 0.05 (s, 6H), 0.90 (s, 9H), 3.02 (s, 3H), 3.73 (s, 2H), 3.98 (br, s, 2H), 4.28 (br s, 1H), 4.46 (d, 2H, *J* = 6.6 Hz), 4.71 (d, 1H, *J* = 12.5 Hz), 4.78 (d, 1H, *J* = 12.5 Hz), 5.50 (s, 1H), 6.29 (dd, 1H, *J* = 2.2, 8.1 Hz), 6.42 (d, 1H, *J* = 2.2 Hz), 6.70 (d, 1H, *J* = 8.1 Hz), 6.88 (br s, 2H), 7.08 (br s, 1H), 7.18-7.42 (m, 8H), 7.61 (br s, 2H), 7.77 (br s, 2H), 7.91 (br s, 1H); HRMS (ESI^{+}): calcd for $[M+H]^{+}$, 726.33632 ; found, 726.34078 (4.45 mmu)

Compound 4l (Fmoc-Phe TBDMS leuco HMRG)

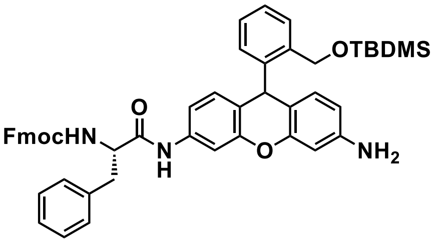

Compound **4l** was synthesized from compound **3** (717 mg, 1.66 mmol), Fmoc-Phe-OH (321 mg, 0.829 mmol), HATU (320 mg, 0.842 mmol) and DIEA (294 μL, 1.66 mmol) following the general procedure. After purification by column chromatography over silica gel using AcOEt/*n*-hexane (1/1) as the eluent and GPC recycle column chromatography using chloroform as the eluent, compound **4l** was obtained (diastereomer mixture, 384 mg, 58 %) as a slightly orange powder. ^{1}H NMR (300 MHz, CD_2Cl_2): δ 0.04 (s, 6H), 0.89 (s, 9H), 3.13 (s, 2H), 3.72 (br s, 2H), 4.20 (t, *J* = 6.6 Hz, 1H), 4.32-4.56 (m, 3H), 4.68 (d, *J* = 12.5 Hz, 1H), 4.75 (d, *J* = 12.5 Hz, 1H), 5.42 (s, 1H), 5.46 (s, 1H), 6.27 (dd, *J* = 8.4, 2.6 Hz, 1H), 6.39 (d, *J* = 2.2 Hz, 1H), 6.63-6.70 (m, 1H), 6.74 (d, *J* = 8.1 Hz, 1H), 6.83 (d, *J* = 8.1 Hz, 1H), 7.01-7.10 (m, 1H), 7.12-7.45 (m, 13H), 7.54 (dd, *J* = 2.9, 7.3 Hz, 2H), 7.61 (s, 1H), 7.77 (d, *J* = 7.3 Hz, 2H); ^{13}C NMR (100 MHz, $CDCl_3$): δ -5.43, 18.3, 25.9, 38.9, 46.8, 57.1, 62.9, 63.0, 67.3, 102.1, 107.9, 110.8, 113.6 (containing 2 peaks), 114.6, 114.7, 119.8, 120.2, 120.4, 124.9, 125.0, 126.5, 127.0, 127.3, 127.4, 127.5, 127.6, 128.6, 129.3, 129.8, 129.9, 130.2, 130.7, 130.8, 136.3, 136.6, 136.7, 138.4, 141.1, 143.3, 143.6, 146.1, 150.7 (containing 2 peaks), 151.1, 156.4, 169.7; HRMS (ESI^{+}): calcd for $[M+H]^{+}$, 802.36762 ; found, 802.36620 (-1.43 mmu)

Compound 4m (Fmoc-Ala TBDMS leuco HMRG)

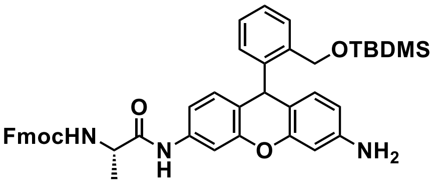

Compound **4m** was synthesized from compound **3** (166 mg, 0.384 mmol), Fmoc-Ala-OH (63 mg, 0.203 mmol), HATU (81 mg, 0.213 mmol) and DIEA (68 μL, 0.384 mmol) following the general procedure. After purification by column chromatography over silica gel using AcOEt/*n*-hexane (1/1) as the eluent and GPC recycle column chromatography using chloroform as the eluent, compound **4m** was obtained (diastereomer mixture, 84 mg, 57 %) as a slightly orange powder. ^{1}H NMR (300 MHz, CD_2Cl_2): δ 0.04 (s, 6H), 0.89 (s, 9H), 1.42 (d, *J* = 6.6 Hz, 3H), 3.72 (s, 2H), 4.17-4.33 (m, 2H), 4.40-4.52 (m, 2H), 4.70 (d, *J* = 12.5 Hz, 1H), 4.77 (d, *J* = 13.2 Hz, 1H), 5.48 (s, 1H), 6.28 (dd, *J* = 8.1, 2.2 Hz, 1H), 6.40 (d, *J* = 2.2 Hz, 1H), 6.69 (d, *J* = 8.8 Hz, 1H), 6.82-6.94 (m, 2H), 7.01-7.11 (m, 1H), 7.12-7.23 (m, 2H), 7.23-7.34 (m, 2H), 7.34-7.47 (m, 4H), 7.60 (d, *J* = 7.3 Hz, 2H), 7.77 (d, *J* = 8.1 Hz, 2H), 7.99 (s, 1H); ^{13}C NMR (100 MHz, $CDCl_3$): δ -5.48, 18.2, 18.7, 18.8, 25.8, 38.9, 46.8, 51.1, 63.0 (containing 2 peaks), 67.2, 102.1, 107.6, 107.7, 110.8, 113.5, 113.6, 114.4, 114.5, 119.8, 120.1, 120.2, 124.8, 124.9, 126.4, 127.0 (containing 2 peaks), 127.3, 127.4, 127.6, 129.9 (containing 2 peaks), 130.2, 130.7 (containing 2 peaks), 137.1, 138.2, 141.1, 143.4, 143.6, 146.1, 150.7, 151.0,

156.3, 170.9; HRMS (ESI$^+$): calcd for $[M+H]^+$, 726.33316 ; found, 726.33362 (-3.16 mmu)

Compound 4n (Fmoc-Arg(Pbf) TBDMS leuco HMRG)

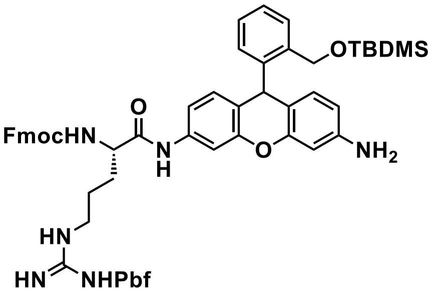

Compound **4n** was synthesized from compound **3** (707 mg, 1.64 mmol), Fmoc-Arg(Pbf)-OH·0.3 IPE·0.1 AcOEt·0.2 Acetone (580 mg, 0.829 mmol), HATU (320 mg, 0.842 mmol) and DIEA (291 μL, 1.64 mmol) following the general procedure. After purification by column chromatography over silica gel using AcOEt/*n*-hexane (3/1) as the eluent and GPC recycle column chromatography using chloroform as the eluent, compound **5n** was obtained (diastereomer mixture, 360 mg, 41 %,) as a slightly orange powder. ^{1}H NMR (300 MHz, CD_2Cl_2): δ 0.03 (s, 6H), 0.88 (s, 9H), 1.38 (s, 6H), 1.51-1.66 (m, 2H), 1.66-1.79 (m, 1H), 1.82-1.97 (m, 1H), 2.03 (s, 3H), 2.43 (s, 3H), 2.54 (s, 3H), 2.88 (s, 2H), 3.25 (s, 2H), 3.69 (s, 2H), 4.13 (t, *J* = 7.0 Hz, 1H), 4.34 (d, *J* = 7.3 Hz, 2H), 4.45 (s, 1H), 4.73 (s, 2H), 5.44 (s, 1H), 6.01 (d, *J* = 8.8 Hz, 1H), 6.14 (s, 2H), 6.25 (dd, *J* = 2.2, 8.1 Hz, 1H), 6.36 (d, *J* = 1.5 Hz, 1H), 6.67 (d, *J* = 8.1 Hz, 1H), 6.79 (d, *J* = 8.1 Hz, 1H), 6.97-7.27 (m, 6H), 7.30-7.43 (m, 3H), 7.49 (d, *J* = 5.1 Hz, 1H), 7.55 (d, *J* = 8.1 Hz, 2H), 7.73 (d, *J* = 7.3 Hz, 2H), 8.91 (s, 1H); ^{13}C NMR (100 MHz, $CDCl_3$): δ -5.42, 12.4, 17.9, 18.3, 19.3, 25.6, 25.9, 28.4, 29.9, 38.7, 40.4, 43.0, 46.9, 55.2, 63.1, 67.1, 86.3, 102.1, 108.0, 110.8, 113.6, 115.0, 117.5, 119.8, 120.3, 124.7, 125.0 (containing 2 peaks), 126.4, 127.0, 127.4, 127.6, 127.7, 129.9, 130.3, 130.7, 132.1, 132.4, 137.1, 138.1, 138.2, 141.0, 143.5, 143.6, 143.9, 146.2, 150.7, 151.1, 156.4, 156.6, 158.8, 170.8; HRMS (ESI$^+$): calcd for $[M+H]^+$, 1063.48233 ; found, 1063.48381 (+1.47 mmu)

Compound 4o (Fmoc-Trp(Boc) TBDMS leuco HMRG)

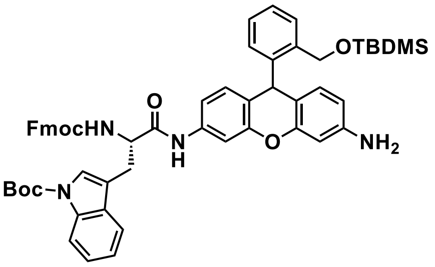

Compound **4o** was synthesized from compound **3** (54 mg, 0.125 mmol), Fmoc-Trp(Boc)-OH (33 mg, 0.0625 mmol), HATU (24 mg, 0.0631 mmol) and DIEA (22 μL, 0.125 mmol) following the general procedure. After purification by column chromatography over silica gel using AcOEt/*n*-hexane (2/3), compound **4o** was obtained (diastereomer mixture, 40 mg, 68 %) as a slightly orange powder. ^{1}H NMR (300 MHz, CD_2Cl_2): δ 0.06 (s, 6H), 0.91 (s, 9H), 1.59 (d, *J* = 3.7 Hz, 9H), 3.25 (d, *J* = 5.9 Hz, 2H), 3.67 (s, 1H), 4.19 (t, *J* = 7.0 Hz, 1H), 4.38 (d, *J* = 6.6 Hz, 2H), 4.63 (s, 1H), 4.72 (d, *J* = 13.2 Hz, 1H), 4.78 (d, *J* = 12.5 Hz, 1H), 5.46 (s, 1H), 5.69 (s, 1H), 6.28 (dd, *J* = 2.2, 8.1 Hz, 1H), 6.38 (s, 1H), 6.70 (d, *J* = 8.1 Hz, 1H), 6.72-6.87 (m, 2H), 7.04 (d, *J* = 8.1 Hz, 1H), 7.12-7.47 (m, 10H), 7.47-7.67 (m, 4H), 7.76 (d, *J* = 7.3 Hz, 2H), 7.93 (s, 1H), 8.13 (d, *J* = 8.8 Hz, 1H); ^{13}C NMR (75 MHz, CD_2Cl_2) : δ -5.26, 18.7, 26.1, 28.2, 28.4, 39.0, 47.5, 56.0, 63.8, 67.6, 84.1, 102.2, 108.2, 111.2, 114.0, 115.1 (containing 2 peaks), 115.6, 115.7, 119.3, 120.3, 121.1, 123.1, 125.0, 125.4, 126.8, 127.5, 128.1, 128.2 ,130.5, 130.8, 131.0, 135.9, 137.1, 138.5, 141.6, 144.1, 145.2, 147.1, 149.8, 151.2, 151.6, 156.6, 169.6; HRMS (ESI$^+$): calcd for $[M+H]^+$, 941.43095 ; found, 941.43159 (0.64 mmu)

Compound 4p (Fmoc-Ser(*t*Bu) TBDMS leuco HMRG)

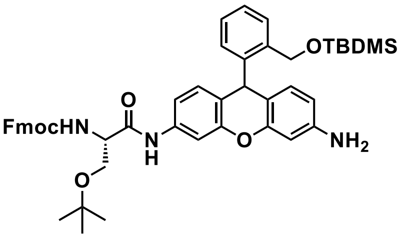

To a solution of compound **3** (697 mg, 1.61 mmol), Fmoc-Ser(*t*Bu)-OH (310 mg, 0.808 mmol) and HATU (310 mg, 0.815 mmol) in DMF (7 mL) was added DIEA (285 μL, 1.61 mmol). The mixture was stirred at 50 °C under Ar atmosphere for 1 h, then cooled to r.t., and AcOEt was added. The organic solution was washed with brine many times, dried over Na_2SO_4 and evaporated to dryness. The residue was dissolved in DMF (7 mL), and to the solution were added Fmoc-Ser(*t*Bu)-OH (309 mg, 0.806 mmol), HATU (304 mg, 0.800 mmol) and DIEA (285 μL, 1.61 mmol). The reaction mixture was stirred at 50 °C under an Ar atmosphere for 1.5 h, then cooled to r.t., and AcOEt was added. The organic solution washed with brine many times, dried over with Na_2SO_4 and evaporated to dryness. The residue was purified by column chromatography over silica gel using CH_2Cl_2/MeOH (19/1) as the eluent to give a crude product, which was subjected to GPC recycle column chromatography to give pure compound **4p** (diastereomer mixture, 314 mg, 49 %) as a slightly orange solid. ^{1}H NMR (300 MHz, CD_2Cl_2): δ 0.04 (s, 6H), 0.89 (s, 9H), 1.24 (s, 9H), 3.46 (t, *J* = 7.7 Hz, 1H), 3.74-3.93 (m, 1H), 4.21-4.34 (m, 2H), 4.44 (d, *J* = 6.6 Hz, 2H), 4.69 (d, *J* = 13.2 Hz, 1H), 4.75 (d, *J* = 12.5 Hz, 1H), 5.49 (s, 1H), 5.78 (s, 1H), 6.28 (dd, *J* = 2.2, 8.1 Hz, 1H), 6.42 (d, *J* = 2.2 Hz, 1H), 6.69 (d, *J* = 8.1 Hz, 1H), 6.81-6.97 (m, 2H), 7.02-7.12 (m, 1H), 7.13-7.24 (m, 2H), 7.25-7.37 (m, 2H), 7.37-7.48 (m, 4H), 7.63 (d, *J* = 7.3 Hz, 2H), 7.79 (d, *J* = 7.3 Hz, 2H), 8.56 (s, 1H); ^{13}C NMR (100 MHz, $CDCl_3$): δ -5.47, 18.3, 25.9, 27.3, 39.2, 47.1, 54.7, 61.8, 63.0, 67.1, 74.6, 102.1, 107.4, 110.9, 113.6, 114.2, 114.3, 119.9, 120.3, 125.0, 126.5, 127.0, 127.4, 127.6 (containing 2 peaks), 130.1 (containing 2 peaks), 130.3, 130.7, 136.9, 138.4, 141.2 (containing 2 peaks), 143.6, 143.7, 146.2, 151.0, 151.1, 156.0, 168.2; HRMS (ESI$^+$): calcd for $[M+H]^+$, 798.39384 ; found, 798.39374 (-0.10 mmu)

Compound 4q (Fmoc-His(Trt) TBDMS leuco HMRG)

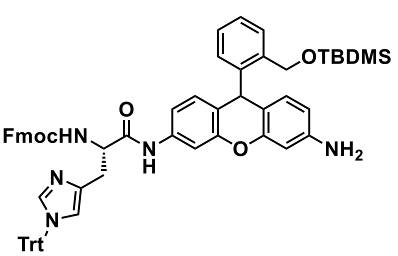

Compound **4q** was synthesized from compound **3** (710 mg, 1.64 mmol), Fmoc-His(Trt)-OH (509 mg, 0.821 mmol), HATU (319 mg, 0.829 mmol) and DIEA (291 μL, 1.64 mmol) following the general procedure. After purification by column chromatography over silica gel using AcOEt/*n*-hexane (1/1) to AcOEt/*n*-hexane (2/1 containing 0.5 % DIEA) as the eluent and GPC recycle column chromatography using chloroform as the eluent, compound **4q** was obtained (diastereomer mixture, 453 mg, 53 %) as a slightly orange powder. ^{1}H NMR (400 MHz, CD_2Cl_2): δ 0.06 (s, 6H), 0.90 (s, 9H), 2.90-2.97 (m, 1H), 3.10-3.14 (m, 1H), 3.73 (br s, 2H), 4.25 (br s, 1H), 4.40 (d, 2H, *J* = 6.3 Hz), 4.53-4.60 (m, 1H), 4.76 (d, 1H, *J* = 12.6 Hz), 4.82 (d, 1H, *J* = 12.6 Hz), 5.51 (s, 1H), 6.29 (dd, 1H, *J* = 2.4, 8.0 Hz), 6.42 (d, 1H, *J* = 2.4 Hz), 6.65 (s, 1H), 6.71 (d, 1H, *J* = 8.0 Hz), 6.80-6.90 (m, 2H), 7.04-7.06 (m, 7H), 7.11-7.25 (m, 13H), 7.40-7.43 (m, 5H), 7.63 (s, 2H), 7.78 (d, 2H, *J* = 6.8 Hz), 9.25 (br s, 1H)**;** ^{13}C NMR (100 MHz, $CDCl_3$): δ -5.44, 18.2, 25.8, 31.1, 31.2, 38.6, 46.9, 55.4, 63.2, 67.0, 75.2, 102.0, 107.4, 110.7, 113.6, 114.3 (containing 2 peaks), 119.7, 120.0, 125.0 (containing 2 peaks), 126.3, 126.9, 127.4, 127.5, 127.6, 127.9, 129.5,

129.9, 130.2, 130.7, 136.3, 137.3, 138.1, 138.4, 141.0, 142.0, 143.6, 143.7, 144.1, 146.2, 150.8, 151.1, 156.0, 169.6**;** HRMS (ESI$^+$): calcd for $[M+H]^+$, 1034.46767 ; found, 1034.46830 (0.63 mmu)

Compound 4r (Fmoc-Met TBDMS leuco HMRG)

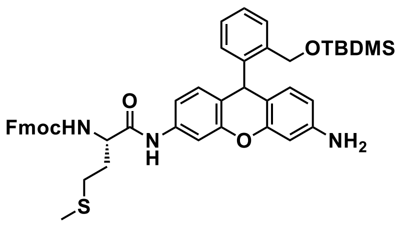

Compound **4r** was synthesized from compound **3** (84 mg, 0.194 mmol), Fmoc-Met-OH (48 mg, 0.129 mmol), HATU (74 mg, 0.195 mmol) and DIEA (100 μL, 0.581 mmol) following the general procedure. After purification by column chromatography over silica gel using AcOEt/*n*-hexane (2/1 to 1/2) as the eluent and GPC recycle column chromatography using chloroform as the eluent, compound **4r** was obtained (diastereomer mixture, 80 mg, 79 %) as a slightly pink powder. ^{1}H NMR (400 MHz, CD_2Cl_2): δ 0.04 (s, 6H), 0.89 (s, 9H), 1.99-2.06 (m, 1H), 2.09 (s, 3H), 2.12-2.19 (m, 1H), 2.53-2.61 (m, 2H), 4.23 (t, *J* = 6.6 Hz, 1H), 4.44-4.46 (m, 3H), 4.70 (d, *J* = 12.8 Hz, 1H), 4.76 (d, *J* = 12.8 Hz, 1H), 5.48 (s, 1H), 5.60 (s, 1H), 6.29 (dd, *J* = 2.3, 8.2 Hz, 1H), 6.41 (d, *J* = 1.8 Hz, 1H), 6.69 (d, *J* = 8.2 Hz, 1H), 6.85-6.95 (m, 2H), 7.05 (d, *J* = 6.9 Hz, 1H), 7.15-7.21 (m, 2H), 7.29-7.32 (m, 2H), 7.36-7.45 (m, 4H), 7.58-7.61 (m, 2H), 7.77 (d, *J* = 7.3 Hz, 2H), 8.18 (s, 1H); ^{13}C NMR (75 MHz, $CDCl_3$): δ -5.40, 15.2, 18.4, 25.9, 30.1, 31.3, 38.9, 47.1, 54.4, 63.2, 67.2, 102.1, 107.6, 107.7, 110.9, 113.8, 114.5 (containing 2 peaks), 120.0, 120.7, 124.9, 126.5, 127.1, 127.5, 127.7, 130.1, 130.2, 130.4, 130.8, 136.7, 138.3, 141.2, 143.5, 143.6, 143.8, 146.2, 150.9, 151.1, 156.4, 169.3; HRMS (ESI$^+$): calcd for $[M+H]^+$, 786.33969 ; found, 786.33663 (-3.06 mmu)

Compound 4s (Fmoc-Gln(Trt) TBDMS leuco HMRG)

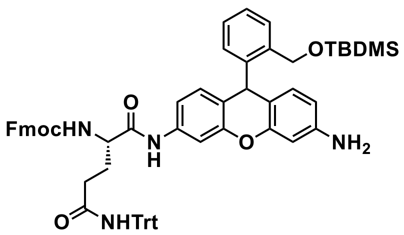

Compound **4s** was synthesized from compound **3** (155 mg, 0.359 mmol), Fmoc-Gln(Trt)-OH (146 mg, 0.239 mmol), HATU (91 mg, 0.239 mmol) and DIEA (83 μL, 0.479 mmol) following the general procedure. After purification by column chromatography over silica gel using AcOEt/*n*-hexane (1/1) as the eluent and GPC recycle column chromatography using chloroform as the eluent, compound **4s** was obtained (diastereomer mixture, 113 mg, 46 %) as a slightly orange powder. ^{1}H NMR (400 MHz, CD_2Cl_2): δ 0.04 (s, 6H), 0.89 (s, 9H), 1.89-2.20 (m, 2H), 2.35-2.53 (m, 1H), 2.55-2.73 (m, 1H), 3.90 (s, 2H), 4.17 (s, 1H), 4.24 (t, *J* = 7.1 Hz, 1H), 4.30-4.51 (m, 2H), 4.70 (d, *J* = 12.8 Hz, 1H), 4.76 (d, *J* = 12.8 Hz, 1H), 5.47 (s, 1H), 6.05 (d, *J* = 5.9 Hz, 1H), 6.28 (dd, *J* = 8.2, 2.3 Hz, 1H), 6.42 (d, *J* = 2.3 Hz, 1H), 6.69 (d, *J* = 8.2 Hz, 1H), 6.74-6.88 (m, 2H), 6.96-7.10 (m, 2H), 7.14-7.20 (m, 2H), 7.20-7.34 (m, 17H), 7.34-7.44 (m, 3H), 7.61 (d, *J* = 6.4 Hz, 2H), 7.78 (d, *J* = 7.3 Hz, 2H), 8.75 (s, 1H), 8.81 (s, 1H); ^{13}C NMR (100 MHz, $CDCl_3$): δ -5.26, 18.5, 26.1, 30.4, 33.9, 39.1, 47.2, 52.4, 63.3, 67.1, 70.1, 102.3, 107.6, 107.7, 111.0, 113.9, 114.7 (containing 2 peaks), 120.1, 120.2, 125.2, 126.6, 127.2 (containing 2 peaks), 127.6, 127.8, 128.1, 128.8, 130.0, 130.5, 130.9, 137.1, 138.4, 141.4, 143.8, 143.9, 144.3, 146.3, 150.9, 151.3, 156.4, 169.4, 172.5; HRMS (ESI$^+$): calcd for $[M+Na]^+$, 1047.44928 ; found, 1047.44661 (-2.67 mmu)

Compound 4t (Fmoc-Asn(Trt) TBDMS leuco HMRG)

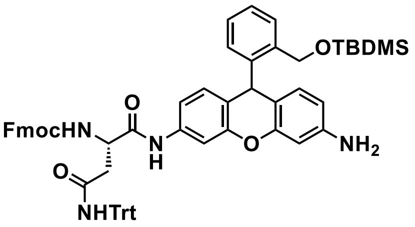

Compound **4t** was synthesized from compound **3** (155 mg, 0.359 mmol), Fmoc-Asn(Trt)-OH (143 mg, 0.239 mmol), HATU (91 mg, 0.239 mmol) and DIEA (83 μL, 0.479 mmol) following the general procedure. After purification by column chromatography over silica gel using AcOEt/*n*-hexane (1/2) as the eluent and GPC recycle column chromatography using chloroform as the eluent, compound **4t** was obtained (diastereomer mixture, 133 mg, 55 %) as a slightly orange powder. ^{1}H NMR (400 MHz, CD_2Cl_2): δ 0.07 (s, 6H), 0.91 (s, 9H), 2.67 (dd, *J* = 6.9, 15.6 Hz, 1H), 3.11 (d, *J* = 14.6 Hz, 1H), 3.94 (s, 2H), 4.23 (t, *J* = 6.9 Hz, 1H), 4.31-4.53 (m, 2H), 4.62 (s, 1H), 4.73 (d, *J* = 12.3 Hz, 1H), 4.82 (dd, *J* = 2.5, 12.6 Hz, 1H), 5.53 (s, 1H), 6.31 (dd, *J* = 2.3, 8.2 Hz, 1H), 6.41 (s, 1H), 6.45 (d, *J* = 1.8 Hz, 1H), 6.72 (d, *J* = 8.2 Hz, 1H), 6.81-6.92 (m, 2H), 7.00 (s, 1H), 7.05-7.12 (m, 1H), 7.14-7.26 (m, 16H), 7.26-7.33 (m, 2H), 7.34-7.47 (m, 4H), 7.60 (d, *J* = 7.3 Hz, 2H), 7.77 (d, *J* = 7.3 Hz, 2H), 8.67 (s, 1H); ^{13}C NMR (100 MHz, $CDCl_3$): δ -5.22, 18.5, 26.1, 38.8, 39.9, 47.2, 52.1, 63.4, 67.3, 71.1, 102.3, 107.7, 107.9, 111.0, 114.0, 114.8 (containing 2 peaks), 120.2, 120.6 (containing 2 peaks), 125.2, 126.7, 127.2, 127.3, 127.7, 127.9, 128.2, 128.7, 130.0, 130.2, 130.6 (containing 2 peaks), 131.0, 136.8, 138.4, 141.4, 143.7, 143.8, 144.1, 146.4, 150.9, 151.0, 151.4, 156.3, 156.4, 168.8, 168.9, 170.8; HRMS (ESI$^+$): calcd for $[M+Na]^+$, 1033.43363 ; found, 1033.43140 (-2.23 mmu)

Compound 4u (Fmoc-Thr(*t*Bu) TBDMS leuco HMRG)

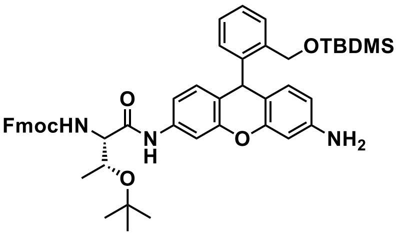

Compound **4u** was synthesized from compound **3** (710 mg, 1.64 mmol), Fmoc-Thr(*t*Bu)-OH (445 mg, 1.12 mmol), HATU (426 mg, 1.12 mmol) and DIEA (395 μL, 2.30 mmol) following the general procedure. After purification by column chromatography over silica gel using AcOEt/*n*-hexane (2/3) as the eluent and GPC recycle column chromatography using chloroform as the eluent, compound **4u** was obtained (diastereomer mixture, 493 mg, 54 %) as a slightly orange powder. ^{1}H NMR (400 MHz, CD_2Cl_2): δ 0.05 (s, 6H), 0.90 (s, 9H), 1.01-1.12 (m, 3H), 1.33 (s, 9H), 3.73 (s, 2H), 4.21 (s, 1H), 4.24-4.34 (m, 2H), 4.34-4.53 (m, 2H), 4.70 (d, *J* = 13.3 Hz, 1H), 4.74 (d, *J* = 13.3 Hz, 1H), 5.49 (s, 1H), 6.00 (s, 1H), 6.29 (dd, *J* = 8.2, 2.3 Hz, 1H), 6.42 (s, 1H), 6.70 (d, *J* = 8.2 Hz, 1H), 6.84-6.98 (m, 2H), 7.03-7.14 (m, 1H), 7.14-7.24 (m, 2H), 7.29-7.48 (m, 6H), 7.65 (d, *J* = 6.9 Hz, 2H), 7.80 (d, *J* = 7.3 Hz, 2H), 9.05 (s, 1H); ^{13}C NMR (100 MHz, $CDCl_3$): δ -5.18, 17.0, 18.6, 26.2, 28.4, 39.5, 47.4, 59.2, 63.3, 67.2, 76.2, 102.3, 107.6, 111.1, 113.7, 114.6, 120.2, 120.4, 125.3, 126.8, 127.3, 127.7, 127.8, 127.9, 130.4 (containing 2 peaks), 130.6, 131.0, 137.1, 138.6, 141.5 (containing 2 peaks), 143.9, 144.1, 146.6, 151.2, 151.4, 156.2, 167.6; HRMS (ESI$^+$): calcd for $[M+Na]^+$, 834.39143 ; found, 834.39078 (-0.66 mmu)

Compound 4v (Fmoc-Ile TBDMS leuco HMRG)

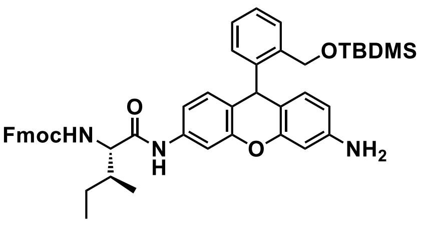

Compound **4v** was synthesized from compound **3** (706 mg, 1.63 mmol), Fmoc-Ile-OH (392 mg, 1.11 mmol), HATU (422 mg, 1.11 mmol) and DIEA (394 μL, 2.29 mmol) following the general procedure. After purification by column chromatography over silica gel using AcOEt/*n*-hexane (1/2) as the eluent and GPC recycle column chromatography using chloroform as the eluent, compound **4v** was obtained (diastereomer mixture, 356 mg, 42 %) as a slightly pink powder. ^{1}H NMR (400 MHz, CD_2Cl_2): δ 0.04 (s, 6H), 0.79-1.01 (m, 15H), 1.17 (s, 1H), 1.47-1.64 (m, 1H), 1.95 (s, 1H), 3.71 (s, 2H), 4.08 (s, 1H), 4.23 (t, J = 6.6 Hz, 1H), 4.34-4.52 (m, 2H), 4.64-4.82 (m, 2H), 5.47 (s, 2H), 6.28 (d, J = 8.2 Hz, 1H), 6.39 (s, 1H), 6.69 (d, J = 8.2 Hz, 1H), 6.80-6.97 (m, 2H), 7.01-7.10 (m, 1H), 7.13-7.23 (m, 2H), 7.23-7.34 (m, 2H), 7.34-7.49 (m, 4H), 7.54-7.64 (m, 2H), 7.77 (d, J = 7.3 Hz, 2H), 7.87 (s, 1H); ^{13}C NMR (100 MHz, $CDCl_3$): δ -5.24, -5.21, 11.3, 15.6, 18.5, 25.2, 26.1, 37.6, 39.1, 39.4, 47.2, 60.7, 63.2, 63.3, 67.4, 102.4, 108.0, 111.0, 113.8, 113.9, 114.8, 115.0, 120.1, 120.3, 120.6, 125.1, 125.2, 126.7, 127.3, 127.5, 127.6, 127.7, 127.9, 130.1, 130.2, 130.5, 130.9, 131.0, 137.1, 138.5, 138.6, 141.4, 143.7, 143.9, 146.4, 151.0 (containing 2 peaks), 151.3, 157.0, 170.6; HRMS (ESI^+): calcd for $[M+H]^+$, 768.38327 ; found, 768.38226 (-1.01 mmu)

Compound 4w (Fmoc-Val TBDMS leuco HMRG)

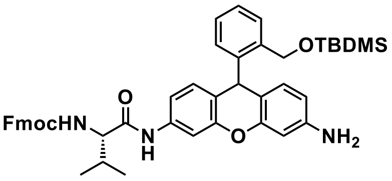

Compound **4w** was synthesized from compound **3** (706 mg, 1.63 mmol), Fmoc-Val-OH (377 mg, 1.11 mmol), HATU (422 mg, 1.11 mmol) and DIEA (394 μL, 2.29 mmol) following the general procedure. After purification by column chromatography over silica gel using AcOEt/ *n*-hexane (1/2) as the eluent and GPC recycle column chromatography using chloroform as the eluent, compound **4r** was obtained (diastereomer mixture, 273 mg, 33 %) as a slightly orange powder. ^{1}H NMR (400 MHz, CD_2Cl_2): δ 0.04 (s, 6H), 0.89 (s, 9H), 0.92-1.14 (m, 6H), 2.20 (s, 1H), 3.73 (s, 2H), 4.03 (s, 1H), 4.24 (t, J = 6.6 Hz, 1H), 4.35-4.55 (m, 2H), 4.70 (d, J = 12.3 Hz, 1H), 4.76 (d, J = 12.3 Hz, 1H), 5.43 (s, 1H), 5.48 (s, 1H), 6.28 (dd, J = 8.2, 1.8 Hz, 1H), 6.40 (d, J = 1.8 Hz, 1H), 6.69 (d, J = 7.8 Hz, 1H), 6.83-6.95 (m, 2H), 7.02-7.11 (m, 1H), 7.13-7.24 (m, 2H), 7.25-7.34 (m, 2H), 7.35-7.49 (m, 4H), 7.60 (d, J = 6.9 Hz, 2H), 7.70-7.85 (m, 3H); ^{13}C NMR (100 MHz, $CDCl_3$): δ -5.23, 18.5, 19.4, 26.1, 31.5, 39.1, 39.3, 47.2, 61.5, 63.2, 63.3, 67.5, 102.3, 108.1, 111.0, 113.8, 113.9, 114.8, 115.0, 120.1, 120.4, 120.6, 125.1, 125.2, 126.7, 127.3, 127.6 (containing 2 peaks), 127.7, 127.9, 130.1, 130.2, 130.5, 130.9, 131.0, 137.1, 138.6, 141.4, 143.6, 143.9, 146.4, 151.0, 151.3, 157.0, 170.3; HRMS (ESI^+): calcd for $[M+Na]^+$, 776.34957 ; found, 776.34943 (-0.14 mmu)

Compound 4x (Fmoc-Asp(*t*Bu) TBDMS leuco HMRG)

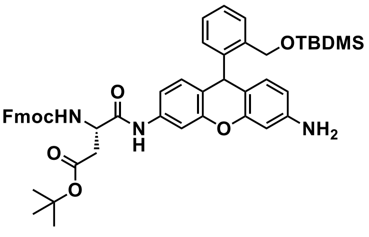

Compound **4x** was synthesized from compound **3** (710 mg, 1.64 mmol), Fmoc-Asp(*t*Bu)-OH (337 mg, 0.82 mmol), HATU (311 mg, 0.82 mmol) and DIEA (291 μL, 1.69 mmol) following the general procedure. After purification by column chromatography over silica gel using AcOEt/*n*-hexane (1/1) as the eluent and GPC recycle column chromatography using chloroform as the eluent,

compound **4x** was obtained (diastereomer mixture, 429 mg, 63 %) as a slightly orange powder. ^{1}H NMR (400 MHz, CD_2Cl_2): δ 0.05 (s, 6H), 0.90 (s, 9H), 1.44 (s, 9H), 2.69 (dd, *J* = 6.4, 16.9 Hz, 1H), 2.90 (dd, *J* = 4.1, 16.9 Hz, 1H), 3.76 (s, 2H), 4.25 (t, *J* = 6.6 Hz, 1H), 4.45 (d, *J* = 6.9 Hz, 2H), 4.59 (s, 1H), 4.70 (d, *J* = 12.8 Hz, 1H), 4.79 (d, *J* = 12.8 Hz, 1H), 5.49 (s, 1H), 5.98 (d, *J* = 5.0 Hz, 1H), 6.29 (dd, *J* = 8.2, 2.3 Hz, 1H), 6.42 (d, *J* = 2.3 Hz, 1H), 6.69 (d, *J* = 8.2 Hz, 1H), 6.83-6.94 (m, 2H), 7.03-7.12 (m, 1H), 7.14-7.23 (m, 2H), 7.26-7.36 (m, 2H), 7.36-7.49 (m, 4H), 7.61 (d, *J* = 7.3 Hz, 2H), 7.78 (d, *J* = 7.3 Hz, 2H), 8.41 (s, 1H); ^{13}C NMR (100 MHz, $CDCl_3$): δ -5.19, 18.6, 26.1, 28.2, 37.8, 39.3, 47.2, 52.0, 63.3, 67.4, 82.3, 102.3, 107.9, 111.1, 113.8, 114.7, 120.2, 120.6, 125.2, 126.7, 127.3, 127.7, 127.9 (containing 2 peaks), 130.2, 130.3, 130.5, 131.0, 137.0, 138.5, 141.4, 143.8, 144.0, 146.5, 151.1, 151.3, 156.5, 168.8, 171.3; HRMS (ESI$^+$): calcd for $[M+Na]^+$, 848.37070 ; found, 848.37105 (0.35 mmu)

Compound 4y (Fmoc-Cys(Trt) TBDMS leuco HMRG)

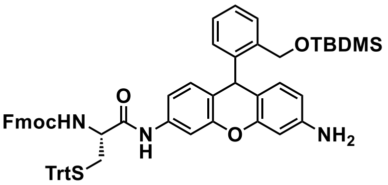

Compound **4y** was synthesized from compound **3** (631 mg, 1.46 mmol), Fmoc-Cys(Trt)-OH (427 mg, 0.73 mmol), HATU (278 mg, 0.732 mmol) and DIEA (259 µL, 1.51 mmol) following the general procedure. After purification by column chromatography over silica gel using AcOEt/*n*-hexane (1/2) as the eluent and GPC recycle column chromatography using chloroform as the eluent, compound **4y** was obtained (diastereomer mixture, 289 mg, 40 %) as a slightly orange powder. ^{1}H NMR (400 MHz, CD_2Cl_2): δ 0.04 (s, 6H), 0.89 (s, 9H), 2.69 (d, *J* = 6.9 Hz, 2H), 3.75-3.89 (m, 1H), 4.21 (t, *J* = 6.4 Hz, 1H), 4.43 (d, *J* = 6.4 Hz, 2H), 4.66 (d, *J* = 12.8 Hz, 1H), 4.79 (d, *J* = 12.8 Hz, 1H), 5.04 (s, 1H), 5.48 (s, 1H), 6.28 (dd, *J* = 2.3, 8.2 Hz, 1H), 6.41 (d, *J* = 1.8 Hz, 1H), 6.69 (d, *J* = 8.2 Hz, 1H), 6.79-6.90 (m, 2H), 7.02-7.08 (m, 1H), 7.14-7.31 (m, 13H), 7.32-7.48 (m, 10H), 7.52-7.67 (m, 3H), 7.70-7.82 (m, 2H); ^{13}C NMR (100 MHz, $CDCl_3$): δ -5.18, 18.6, 26.1, 33.9, 39.2, 47.2, 54.9, 63.4, 67.3, 67.6, 102.3, 107.9 (containing 2 peaks), 111.1, 113.9, 114.7 (containing 2 peaks), 120.1, 120.7 (containing 2 peaks), 125.1, 126.7, 127.1, 127.3, 127.7, 127.9, 128.3, 129.7, 130.2, 130.3, 130.5, 131.0, 136.8, 138.5, 141.4, 143.7 (containing 2 peaks), 144.0, 144.5, 146.4, 151.0, 151.3, 156.5, 168.5; HRMS (ESI$^+$): calcd for $[M+Na]^+$, 1022.39989 ; found, 1022.40256 (2.67 mmu)

General procedure for synthesis of P2-P1-HMRG

This procedure consists of 3 parts.

[1] Introduction of compound 4a-4y onto 2-chlorotrityl chloride resin

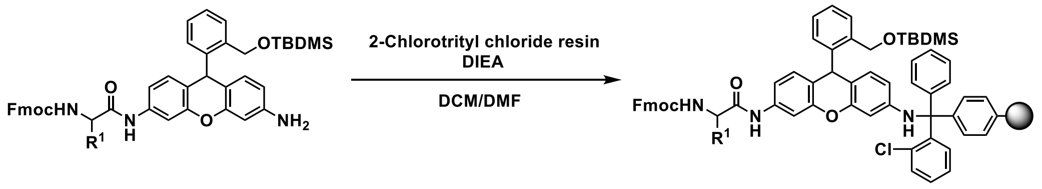

To a solution of compound **4a-4y** (1-1.5 eq) in DMF 1.6 mL and CH_2Cl_2 0.4 mL were added 2-chlorotrityl chloride resin (co(polystyrene-1% DVB), 1.00-1.60 mmol/g) (0.024X mmol, 1 eq) and DIEA (200 μL). The reaction mixture was stirred overnight at r.t. under an Ar atmosphere and protected from light. The resin was collected by filtration, washed with CH_2Cl_2 many times, divided into X aliquots containing 0.024 mmol each, and used for the next reaction in a peptide synthesizer.

[2] Reaction with peptide synthesizer

Peptide synthesizer protocols A-C were as follows.

Peptide synthesizer protocol A (Oxidation with chloranil and Fmoc deprotection)

To the resin (0.024 mmol) was added DMF (1.1 mL). The mixture was shaken for 1 h to swell the resin. DMF was removed by filtration, and chloranil (4 eq) in DMF was added. The mixture was shaken for 1 h, and the reaction solution was removed by filtration. To the resin was added 40 % piperidine in DMF (800 μL). The mixture was shaken for 3 min, and the reaction solution was removed by filtration. Then 40 % piperidine in DMF (400 μL) was added to the resin. The mixture was shaken for 12 min, and the reaction solution was removed by filtration. The resin was washed with DMF (900 μL) with shaking for 1 min, and then collected by filtration. This washing procedure was repeated five more times.

Peptide synthesizer protocol B (Condensation reaction and Fmoc deprotection)

To the resin (0.024 mmol) were added Fmoc-Amino acid (4 eq) in DMF, HATU (4 eq) in DMF and 2 M DIEA in NMP (200 μL), and the mixture was shaken for 2 h. The reaction solution was removed by filtration. The resin was washed with DMF (900 μL) with shaking for 1 min, and then collected by filtration. This washing procedure was repeated two more times. The condensation procedure was repeated once more. To the resin (0.024 mmol) were add Fmoc-amino acid (4 eq) in DMF, HATU (4 eq) in DMF and 2 M DIEA in NMP (200 μL). The mixture was shaken for 1 h, and then filtered. The resin was washed with DMF (900 μL) with shaking for 1 min, and collected by filtration. This washing procedure was repeated two more times.

Peptide synthesizer protocol C (Acetylation of N-terminal amino group)

The resin (0.024 mmol) was swollen in DMF 1.1 mL for 1 h, and then collected by filtration. To the resin were added DMF (400 μL), 2 M DIEA in NMP (400 μL) and acetic anhydride (400 μL). The mixture was shaken for 1.5 h and the resin was collected by filtration. These procedures for acetylation were repeated two more times. The resin was washed with DMF (900 μL) with shaking for 1 min, and collected by filtration. This washing procedure was repeated two more times.

General procedure for synthesis of mono amino acid HMRG.

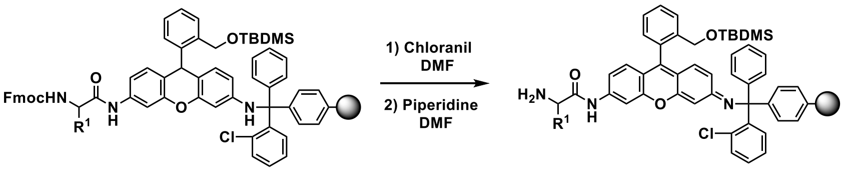

The resin (0.024 mmol) bearing compound **4a-4y** was treated according to protocol A and used for the next cleavage reaction.

General procedure for synthesis of acetyl mono amino acid HMRG

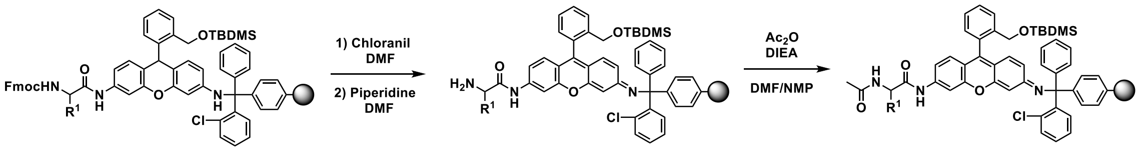

The resin (0.024 mmol) bearing compound **4a-4y** was treated according to protocol A and protocol C in turn and used for the next cleavage reaction.

General procedure for synthesis of dipeptidyl HMRG

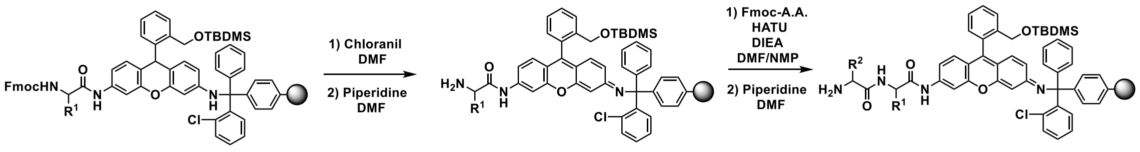

The resin (0.024 mmol) bearing compound **4a-4y** was treated according to protocol A and protocol B in turn and used for the next cleavage reaction.

General procedure for synthesis of acetyl dipeptide HMRG

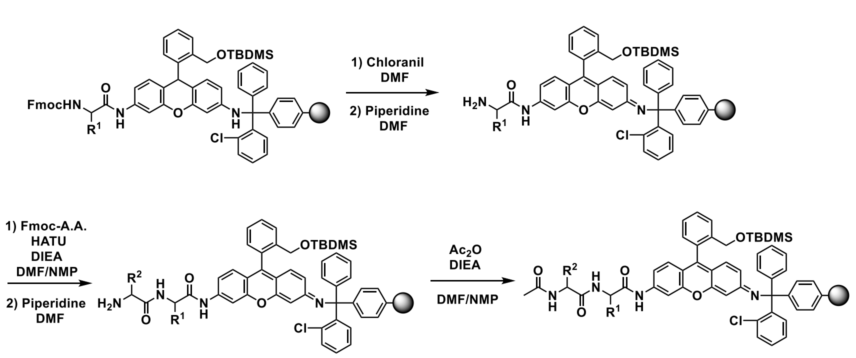

The resin (0.024 mmol) bearing compound **4a**-**4y** was treated according to protocol A, protocol B and protocol C in turn, and used for the next cleavage reaction.

[3] Cleavage from resin

To the resin (0.024 mmol) after the reaction in the peptide synthesizer were added TFA (2 mL), TES (200 μL) and H_2O (200 μL). The mixture was stirred for 2 h, and filtered. The filtrate was evaporated to dryness. The residue was taken up in Et_2O 20 mL (for AcL-HMRG, Et_2O/*n*-hexane (1/1) 20 mL was used) at 4 °C to perform ether precipitation. The mixture was centrifuged (3,000 rpm, 5 min), the supernatant was discarded, and the residue was air-dried to give crude P2-P1-HMRG. The crude compound was dissolved in DMSO and analyzed by means of LC-MS. If the purity of crude P2-P1-HMRG at either 254 nm or 490 nm was less than 80 %, purification was performed with HPLC using eluent A and B to give P2-P1-HMRG with sufficient purity. If necessary, additional purification was performed with HPLC using eluent E and F to give P2-P1-HMRG with sufficient purity.

Synthesis of KK-HMRG

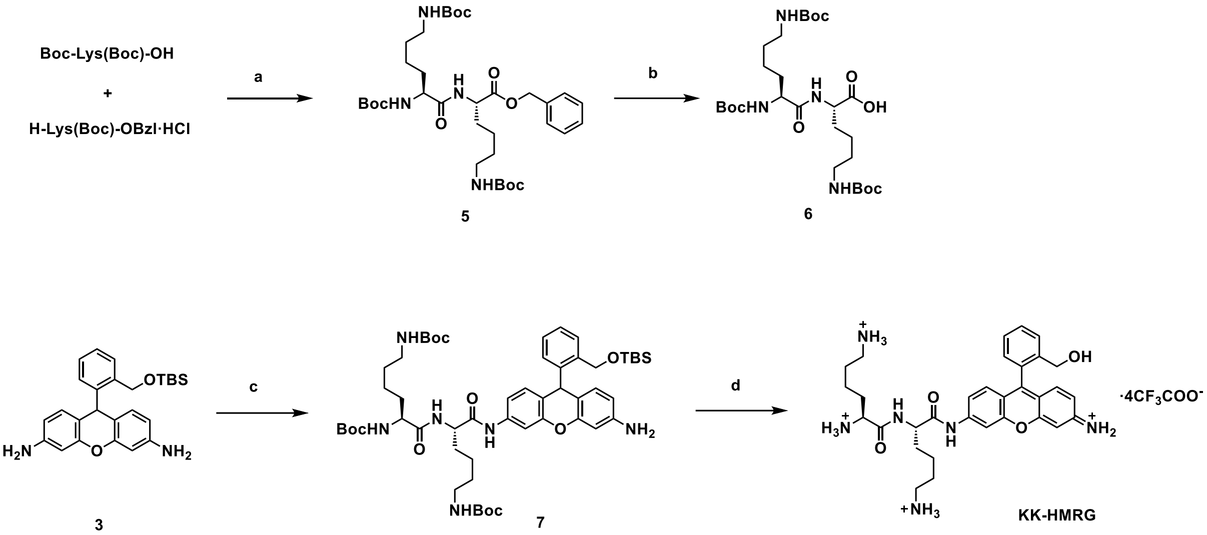

Scheme S2. Synthetic route to KK-HMRG.

(a) HATU, DIEA, DMF, y. 74 %, (b) 10 % H_2, Pd/C, THF crude, (c) **6**, HATU, DIEA, DMF, crude, (d) 1) Chloranil, DCM, MeOH, 2) TFA, MeCN, y. 29 % in 4 steps from **5**.

Compound 5

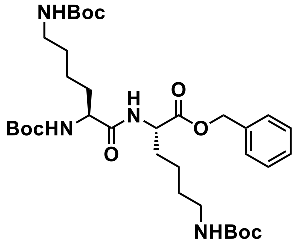

To a solution of Boc-Lys(Boc)-OH (346 mg, 1.00 mmol), H-Lys(Boc)-OBzl·HCl (372 mg, 1.00 mmol) and DIEA (516 µL, 3.00 mmol) was added HATU (418 mg, 1.10 mmol). The mixture was stirred at room temperature for 1 h and then diluted with AcOEt. The organic layer was washed with sat. NH_4Cl aq. three times, dried over Na_2SO_4 and evaporated to dryness. The residue was purified by column chromatography over silica gel using AcOEt/*n*-hexane (1/3 to 1/1) as the eluent to give compound **5** (490 mg, 74 %) as a colorless solid. ^{1}H NMR (400 MHz, CD_3OD): δ 1.33-1.43 (m, 35H), 1.50-1.59 (m, 1H), 1.65-1.76 (m, 2H), 1.81-1.89 (m, 1H), 2.96-3.00 (m, 4H), 4.02 (dd, *J* = 8.0, 5.3 Hz, 1H), 4.43 (dd, *J* = 8.7, 5.0 Hz, 1H), 5.12 (d, *J* = 12.3 Hz, 1H), 5.18 (d, *J* = 12.3 Hz, 1H), 7.29-7.39 (m, 5H); ^{13}C NMR (100 MHz, $CDCl_3$): δ 22.7, 27.4, 27.5, 29.0, 29.2, 30.8, 31.7, 39.7, 39.8, 52.4, 54.4, 66.6, 78.5, 79.2, 128.1 (containing 2 peaks), 128.3, 135.9, 156.5, 157.2, 172.0, 174.0; HRMS (ESI$^+$): calcd for $[M+Na]^+$, 687.39450 ; found, 687.39307 (-1.43 mmu)

Compound 6

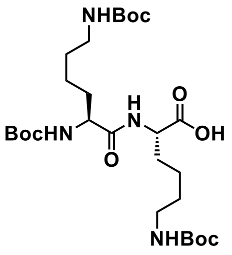

To a solution of compound **5** (67 mg, 0.101 mmol) in THF (10 mL) was added 10 % Pd/C (2.3 mg). The mixture was stirred for 45 min under an H_2 atmosphere. Because the reaction was not complete, 10 % Pd/C (5.0 mg) was added and stirring was continued for 2.5 h under an H_2 atmosphere. The reaction mixture was filtered through a pad of Celite and evaporated to dryness to give crude **6**. This was used for the next reaction without further purification.

KK-HMRG

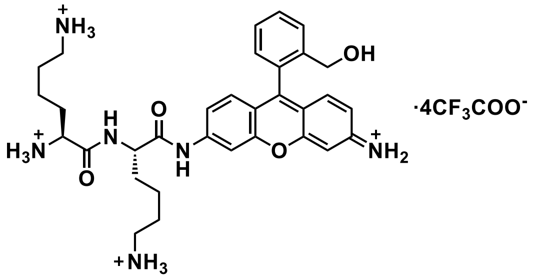

To a solution of crude **6** in DMF (5 mL) was added compound **3** (87 mg, 0.201 mmol), HATU (76 mg, 0.200 mmol) and DIEA (51 µL, 0.300 mmol). The mixture was stirred at 50 °C under an argon atmosphere for 2 h and then cooled to room temperature. Sat. NH_4Cl aq. was added and the whole was extracted with AcOEt three times. The organic layer was combined, dried over Na_2SO_4 and evaporated to dryness. The residue was purified by column chromatography over silica gel using AcOEt/*n*-hexane (gradient from 30/70 to 99/1) as the eluent to give crude **7**. The crude **7** (35 mg) was dissolved in DCM/MeOH (5 mL/5 mL), and chloranil (10 mg, 0.041 mmol) was added. The mixture was stirred at room temperature for 1 h and evaporated to dryness. The residue was dissolved in TFA/MeCN (2 mL/6 mL) and the solution was stirred at room temperature for 45 min. The reaction mixture was evaporated to dryness. The residue was purified by HPLC (eluent; A/B = linear gradient from 90/10 to 10/90 in 60 min) to give **KK-HMRG** (30 mg, 29 % in 4 steps from **5**) as a red powder. ^1H NMR (400 MHz, CD_3OD+NaOD in D_2O): δ 1.36-1.88 (m, 12H), 2.54-2.62 (m, 4H), 3.32-3.36 (m, 1H), 4.40-4.45 (m, 1H), 5.22 (s, 2H), 6.39 (dd, *J* = 8.5, 2.1 Hz, 1H), 6.48 (d, *J* = 1.8 Hz, 1H), 6.62 (d, *J* = 8.7 Hz, 1H), 6.77-6.82 (m, 2H), 7.08 (d, *J* = 8.7 Hz, 1H), 7.25 (t, *J* = 7.1 Hz, 1H), 7.34-7.40 (m, 2H), 7.57 (d, *J* = 1.8 Hz, 1H); ^{13}C NMR (100 MHz, CD_3OD+NaOD in D_2O): δ 23.9, 24.1, 24.3, 24.4, 33.1, 33.4, 33.5, 33.8, 36.1, 36.4, 42.3 (containing 2 peaks), 42.4, 55.4, 55.9, 56.0, 72.8, 85.4, 101.9, 108.4, 108.6, 112.8, 114.6, 116.1, 116.2, 121.9, 124.7, 129.3, 129.4, 130.4, 130.8, 140.2, 140.4, 140.5, 146.1, 150.7, 152.2, 152.8, 172.9, 173.0, 177.8, 178.0; HRMS (ESI$^+$): calcd for $[M]^+$, 573.31893 ; found, 573.31761 (-1.32 mmu)

KH-HMRG (a kind gift from Goryo Chemical, Inc.). ^1H NMR (400 MHz, D_2O+NaOD): δ 7.54 (s, 1H), 7.43-7.37 (m, 2H), 7.27 (t, *J* = 7.8 Hz, 1H), 7.05 (s, 1H), 6.86-6.74 (m, 4H), 6.71 (d, *J* = 8.5 Hz, 1H), 6.59 (d, *J* = 2.2 Hz, 1H), 6.47 (dd, *J* = 8.5, 2.2 Hz, 1H), 5.19 (s, 2H), 4.57-4.53 (m, 1H), 2.39 (t, *J* = 7.1 Hz, 1H), 1.49-1.43 (m, 1H), 1.28-1.24 (m, 1H), 1.10-1.06 (m, 1H). [Note: proton peaks of the α-proton (1H) and imidazolyl methyl proton (2H) of peptide moiety were not observed due to overlap with solvent peaks of water and methanol]; HRMS (ESI$^+$): calcd for $[M]^+$, 582.28233; found, 582.28364 (-1.3 mmu).

Synthesis of KK-AMC

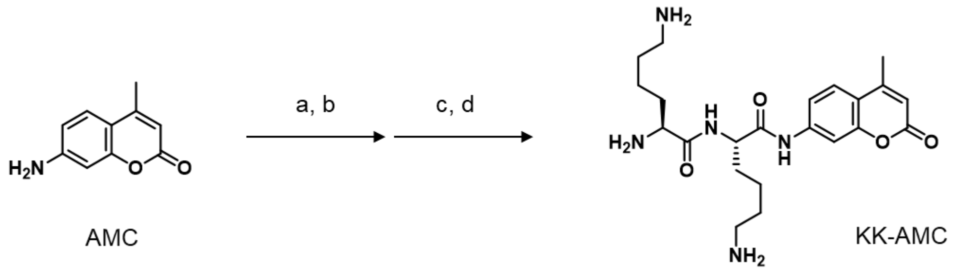

Scheme S3. Synthetic scheme for KK-AMC. a) Fmoc-Lys(Boc)-OH, COMU, DIEA, DMF; b) 20% piperidine in DMF; c) Boc-Lys(Boc)-OH, HATU, DIEA, DMF; d) TFA/H_2O/TIS.

Synthesis of KK-AMC. A solution of 7-amino-4-methylcoumarin (AMC) (200 mg, 1.14 mmol), Fmoc-Lys(Boc)-OH (2.67 g, 5.65 mmol), COMU (2.44 g, 5.70 mmol) and diisopropylethylamine (DIEA) (1.98 mL) in 10 mL DMF was stirred at 65 °C for 12 h. Then, the solvent was evaporated under reduced pressure. The residue was purified by flash column chromatography (eluent; hexane/AcOEt = 10/90 to 0/100) to afford crude Fmoc-Lys(Boc)-AMC. A solution of DMF containing 20% piperidine (1.5 mL) was added, and the mixture was stirred at room temperature for 30 min, followed by purification by HPLC to obtain Fmoc-deprotected intermediate. Then, a solution of Boc-Lys(Boc)-OH (200 mg, 0.379 mmol), HATU (313 mg, 7.58 mmol), and DIEA (330 μL) in 1.5 mL DMF was added, and the reaction mixture was stirred at room temperature for 1 h. The mixture was separated by HPLC to afford Boc-Lys(Boc)-Lys(Boc)-AMC. Then, a solution of TFA containing 2.5% H_2O and 2.5% triisopropylsilane (TIS) (400 μL) was added, and the mixture was stirred at room temperature for 30 min, followed by purification by HPLC to afford **KK-AMC** (5.10 mg, 1 % in 4 steps) as a slightly yellow solid. Gradient elution in HPLC: A/B = 90/10 to 10/90 in 30 min (eluent A: H_2O containing 1 % acetonitrile and 0.1 % TFA, eluent B: acetonitrile containing 1 % H_2O). ^{1}H NMR (400 MHz, CD_3OD): δ 7.82 (d, J = 2.0 Hz, 0.3H), 7.65 (d, J = 8.8 Hz, 1H), 7.30 (dd, J = 2.0 Hz, 8.8 Hz, 1H), 6.17 (d, J = 1.2 Hz, 1H), 4.43 (m, 1H), 3.90 (m, 1H), 3.18-3.11 (m, 1H), 2.85 (m, 4H), 2.37 (s, 3H), 1.84 (m, 4H), 1.60 (m, 4H), 1.44 (m, 4H). HRMS (ESI$^+$): calcd for $[M+Na]^+$, 454.24160; found, 454.24248 (-0.9 mmu).

Synthesis of KH-AMC

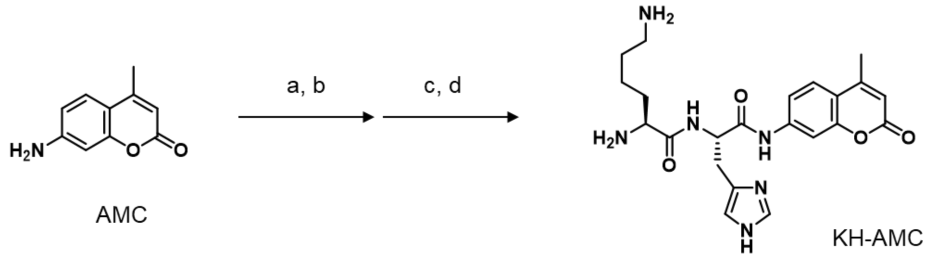

Scheme S4. Synthetic scheme for KH-AMC. a) Fmoc-His(Trt)-OH, COMU, DIEA, DMF; b) 20% piperidine in DMF; c) Boc-Lys(Boc)-OH, HATU, DIEA, DMF; d) TFA/ H_2O/TIS.

Synthesis of KH-AMC. A solution of 7-amino-4-methylcoumarin (AMC) (200 mg, 1.14 mmol), Fmoc-His(Trt)-OH (3.54 g, 5.71 mmol), COMU (2.44 g, 5.70 mmol) and diisopropylethylamine (DIEA) (1.98 mL)

in 10 mL DMF was stirred at 65 °C for 12 h. Then, the solvent was evaporated under reduced pressure. The residue was purified by flash column chromatography (eluent; hexane/AcOEt = 10/90 to 0/100) to afford crude Fmoc-His(Trt)-AMC. A solution of 20% piperidine in DMF (1.5 mL) was added, and the mixture was stirred at room temperature for 30 min, followed by purification by HPLC to obtain the Fmoc-deprotected intermediate. Then, a solution of Boc-Lys(Boc)-OH (158 mg, 0.456 mmol), HATU (188 mg, 0.455 mmol), DIEA (390 μL) in 1.5 mL DMF was added, and the reaction mixture was stirred at room temperature for 1 h. The mixture was separated by HPLC to afford Boc-Lys(Boc)-His(Trt)-AMC. Then, a solution of TFA containing 2.5% H_2O and 2.5% triisopropylsilane (TIS) (400 μL) was added. The mixture was stirred at room temperature for 30 min, followed by purification by HPLC to afford **KH-AMC** (12.6 mg, 0.0286 mmol, 2.5 % in 4 steps) as a slightly yellow solid. Gradient elution in HPLC: A/B = 90/10 to 10/90 in 30 min (eluent A: H_2O containing 1 % acetonitrile and 0.1 % TFA, eluent B: acetonitrile containing 1 % H_2O). ^{1}H NMR (400 MHz, CD_3OD): δ 8.68 (d, *J* = 6.8 Hz, 1H), 7.80 (d, *J* = 6.8 Hz, 0.3H), 7.75 (d, *J* = 6.8 Hz, 0.7H), 7.64 (m, 1H), 7.36 (dd, *J* = 2.0 Hz, 8.4 Hz, 1H), 7.29 (s, 1H), 6.17 (d, *J* = 1.2 Hz, 1H), 3.91-8.83 (m, 2H), 3.32-3.25 (m, 1H), 3.18-3.11 (m, 1H), 2.83 (t, *J* = 7.6 Hz, 2H), 2.36 (d, *J* = 1.2 Hz, 3H), 1.79 (m, 2H), 1.60 (m, 2H), 1.33 (m, 2H). HRMS (ESI$^+$): calcd. for $[M+Na]^+$, 463.20642; found, 463.20612 (0.3 mmu).

Supplementary Figures and Tables

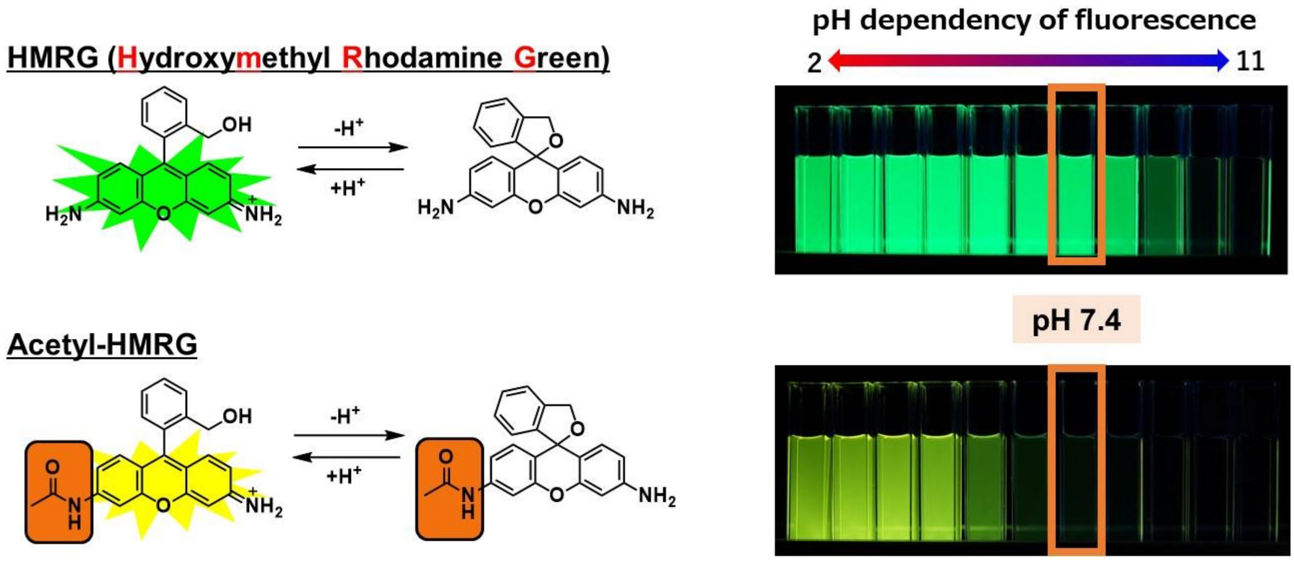

Figure S1. pH dependency of fluorescence of HMRG and acetyl-HMRG[7].

(Left) pH-dependent equilibrium of spirocyclization of HMRG and acetyl-HMRG. These compounds are highly fluorescent only when they exist in the open form. (Right) pH-dependent fluorescence of these compounds. At physiological pH (around pH 7.4), HMRG exists in the open form, whereas acetyl-HMRG is in the closed form.

NO.	Substrate moiety	LRMS [M]$^+$	Purity at 254 nm (%)	Purity at 490 nm (%)	HPLC purification	Analytical method
**YK0001**	GG	431	89	97	✔	A
**YK0002**	EG	503	89	90	✔	A
**YK0003**	KG	502	98	99	✔	A
**YK0004**	YG	537	96	97	✔	A
**YK0005**	LG	487	87	94	✔	A
**YK0006**	PG	471	89	94	✔	A
**YK0007**	GE	503	87	88		A
**YK0008**	EE	575	89	91		A
**YK0009**	KE	574	86	86		A
**YK0010**	YE	609	86	85		A
**YK0011**	LE	559	91	92		A
**YK0012**	PE	543	85	87		A
**YK0013**	GK	502	85	89		A
**YK0014**	EK	574	86	87		A
**YK0015**	KK	573	97	97	✔	B
**YK0016**	YK	608	89	88		A
**YK0017**	LK	559	88	89		A
**YK0018**	PK	542	87	89		A
**YK0019**	GY	537	85	97		A
**YK0020**	EY	609	88	93		A
**YK0021**	KY	608	89	93		A
**YK0022**	YY	643	92	95		A
**YK0023**	LY	593	86	94		A
**YK0024**	PY	577	91	95		A
**YK0025**	GL	487	91	93		B
**YK0026**	EL	559	93	93	✔	A
**YK0027**	MeG	388	99	99	✔	D
**YK0028**	KL	558	97	97	✔	A
**YK0029**	YL	593	86	86		A
**YK0030**	LL	543	91	95		A
**YK0031**	PL	527	89	93		A
**YK0032**	GP	471	96	99	✔	A
**YK0033**	EP	543	97	98	✔	A
**YK0034**	KP	542	97	98	✔	A

**YK0035**	YP	577	97	97	✔	B
**YK0036**	LP	527	99	99	✔	B
**YK0037**	PP	511	98	99	✔	A
**YK0038**	aG	445	98	99	✔	A
**YK0039**	dG	489	86	92		A
**YK0040**	sG	461	99	99	✔	B
**YK0041**	bG	445	86	94		A
**YK0042**	MeGG	445	90	96		A
**YK0043**	aE	517	88	95		A
**YK0044**	dE	561	91	95		A
**YK0045**	sE	533	85	88		A
**YK0046**	bE	517	99	99	✔	B
**YK0047**	MeGE	517	89	94		A
**YK0048**	aK	516	90	91		A
**YK0049**	dK	560	91	93		B
**YK0050**	sK	532	98	98	✔	B
**YK0051**	bK	516	87	91		B
**YK0052**	MeGK	516	89	92		B
**YK0053**	aY	551	92	96		B
**YK0054**	dY	595	93	95		B
**YK0055**	sY	567	87	89		B
**YK0056**	bY	551	93	95		B
**YK0057**	MeGY	551	90	94		B
**YK0058**	aL	501	93	92		B
**YK0059**	dL	545	95	97		B
**YK0060**	sL	517	95	99	✔	B
**YK0061**	bL	501	90	95		B
**YK0062**	MeGL	501	95	97		B
**YK0063**	aP	485	98	99	✔	B
**YK0064**	dP	529	96	97	✔	B
**YK0065**	sP	501	96	97	✔	B
**YK0066**	bP	485	88	99	✔	B
**YK0067**	MeGP	485	93	99	✔	B
**YK0068**	Ga	445	90	95		B
**YK0069**	Ea	517	88	92		B
**YK0070**	Ka	516	87	93		B
**YK0071**	Ya	551	87	93		B

**YK0072**	La	501	89	95		B
**YK0073**	Pa	485	90	94		B
**YK0074**	aa	459	91	97		B
**YK0075**	da	503	93	98		B
**YK0076**	sa	475	96	99	✔	B
**YK0077**	ba	459	91	97		B
**YK0078**	MeGa	459	91	96		B
**YK0079**	Gd	489	93	96		B
**YK0080**	Ed	561	92	96		B
**YK0081**	Kd	560	88	89		B
**YK0082**	Yd	595	90	92		B
**YK0083**	Ld	545	89	92		B
**YK0084**	Pd	529	88	93		B
**YK0085**	ad	503	93	93		B
**YK0086**	dd	547	93	93		B
**YK0087**	sd	519	87	87		B
**YK0088**	bd	503	94	96		B
**YK0089**	MeGd	503	92	95		B
**YK0090**	Gs	461	88	94		B
**YK0091**	Es	533	90	94		B
**YK0092**	Ks	532	89	91		B
**YK0093**	Ys	567	89	92		B
**YK0094**	Ls	517	89	91		B
**YK0095**	Ps	501	92	95		B
**YK0096**	as	475	93	95		B
**YK0097**	ds	519	91	95		B
**YK0098**	ss	491	85	86		B
**YK0099**	bs	475	88	93		B
**YK0100**	MeGs	475	92	96		B
**YK0101**	Gb	445	94	95		B
**YK0102**	Eb	517	91	94		B
**YK0103**	Kb	516	93	95		B
**YK0104**	Yb	551	85	87		B
**YK0105**	Lb	501	96	97		B
**YK0106**	Pb	485	96	98		B
**YK0107**	ab	459	96	97		B
**YK0108**	db	503	94	95		B

**YK0109**	sb	475	85	86		B
**YK0110**	bb	459	95	96		B
**YK0111**	MeGb	459	96	97		B
**YK0112**	GMeG	445	88	90	✔	B
**YK0113**	EMeG	517	99	99	✔	B
**YK0114**	KMeG	516	99	99	✔	B
**YK0115**	YMeG	551	95	95	✔	B
**YK0116**	LMeG	501	100	99	✔	B
**YK0117**	PMeG	485	97	97	✔	B
**YK0118**	aMeG	459	98	99	✔	B
**YK0119**	dMeG	503	97	95	✔	B
**YK0120**	sMeG	475	92	93	✔	B
**YK0121**	bMeG	459	94	96	✔	B
**YK0122**	MeGMeG	459	99	99	✔	B
**YK0123**	AcG	416	99	99	✔	B
**YK0124**	AcGG	473	85	86		C
**YK0125**	AcEG	545	87	89		C
**YK0126**	AcKG	544	89	93		C
**YK0127**	AcYG	579	85	88		C
**YK0128**	AcLG	529	88	89		C
**YK0129**	AcPG	513	89	91		C
**YK0130**	E	446	94	98	✔	B
**YK0131**	AcE	488	97	97	✔	B
**YK0132**	AcGE	545	89	94		C
**YK0133**	AcEE	617	94	96		C
**YK0134**	AcKE	616	93	96		C
**YK0135**	AcYE	651	92	94		C
**YK0136**	AcLE	601	90	93		C
**YK0137**	AcPE	585	93	95		C
**YK0138**	K	445	100	100	✔	C
**YK0139**	AcK	487	99	99	✔	C
**YK0140**	AcGK	544	85	87		B
**YK0141**	AcEK	616	89	90		B
**YK0142**	AcKK	615	89	88		B
**YK0143**	AcYK	650	86	87		B
**YK0144**	AcLK	600	85	87		B
**YK0145**	AcPK	584	89	89		B

**YK0146**	Y	480	99	99	✔	B
**YK0147**	AcY	522	94	93	✔	B
**YK0148**	AcGY	579	94	96		B
**YK0149**	AcEY	651	95	96		B
**YK0150**	AcKY	650	91	92		B
**YK0151**	AcYY	685	89	91		B
**YK0152**	AcLY	635	87	88		B
**YK0153**	AcPY	619	91	92		B
**YK0154**	L	430	99	100	✔	C
**YK0155**	AcL	472	91	97	✔	B
**YK0156**	AcGL	529	100	99	✔	C
**YK0157**	AcEL	601	100	100	✔	C
**YK0158**	AcKL	600	100	100	✔	C
**YK0159**	AcYL	635	99	99	✔	C
**YK0160**	AcLL	585	100	100	✔	C
**YK0161**	AcPL	569	100	100	✔	C
**YK0162**	P	414	99	99	✔	C
**YK0163**	AcP	456	99	98	✔	C
**YK0164**	AcGP	513	98	97	✔	C
**YK0165**	AcEP	585	96	98	✔	C
**YK0166**	AcKP	584	100	100	✔	C
**YK0167**	AcLP	619	100	100	✔	C
**YK0168**	AcYP	569	97	98	✔	C
**YK0169**	AcPP	553	94	96	✔	C
**YK0170**	F	464	93	92		B
**YK0171**	GF	521	91	95		B
**YK0172**	EF	593	93	95		B
**YK0173**	KF	592	93	95		B
**YK0174**	YF	627	91	93		B
**YK0175**	LF	577	94	95		B
**YK0176**	PF	561	95	96		B
**YK0177**	AcF	506	99	99	✔	B
**YK0178**	AcGF	563	91	91		B
**YK0179**	AcEF	635	93	95		B
**YK0180**	AcKF	634	93	95		B
**YK0181**	AcYF	669	92	94		B
**YK0182**	AcLF	619	87	87		B

**YK0183**	AcPF	603	92	94		B
**YK0184**	R	473	99	99	✔	B
**YK0185**	GR	530	98	98	✔	B
**YK0186**	ER	602	96	97	✔	B
**YK0187**	KR	601	97	99	✔	C
**YK0188**	YR	636	98	99	✔	C
**YK0189**	LR	586	99	98	✔	C
**YK0190**	PR	570	98	99	✔	B
**YK0191**	AcR	515	99	99	✔	B
**YK0192**	AcGR	572	90	87	✔	C
**YK0193**	AcER	644	99	99	✔	B
**YK0194**	AcKR	643	98	98	✔	B
**YK0195**	AcYR	678	97	99	✔	B
**YK0196**	AcLR	628	98	99	✔	B
**YK0197**	AcPR	612	99	99	✔	B
**YK0198**	A	388	96	99	✔	C
**YK0199**	GA	445	92	94		B
**YK0200**	EA	517	93	94		B
**YK0201**	KA	516	92	94		B
**YK0202**	YA	551	91	93		B
**YK0203**	LA	501	91	95		B
**YK0204**	PA	485	91	95		B
**YK0205**	AcA	430	100	100	✔	C
**YK0206**	AcGA	487	89	96		B
**YK0207**	AcEA	559	95	97		B
**YK0208**	AcKA	558	93	95		B
**YK0209**	AcYA	593	93	96		B
**YK0210**	AcLA	543	90	93		B
**YK0211**	AcPA	527	94	96		B
**YK0212**	H	454	97	99	✔	C
**YK0213**	GH	511	97	99	✔	C
**YK0214**	EH	583	97	99	✔	C
**YK0215**	KH	582	99	99	✔	C
**YK0216**	YH	617	88	99	✔	C
**YK0217**	LH	567	93	98	✔	C
**YK0218**	PH	551	96	99	✔	C
**YK0219**	AcH	496	93	99	✔	C

**YK0220**	AcGH	553	94	98	✔	C
**YK0221**	AcEH	625	98	99	✔	C
**YK0222**	AcKH	624	98	100	✔	C
**YK0223**	AcYH	659	95	99	✔	C
**YK0224**	AcLH	609	96	100	✔	C
**YK0225**	AcPH	593	95	99	✔	C
**YK0226**	W	503	95	96	✔	C
**YK0227**	GW	560	98	99	✔	C
**YK0228**	EW	632	99	99	✔	C
**YK0229**	KW	631	95	97	✔	C
**YK0230**	YW	666	97	98	✔	C
**YK0231**	LW	616	99	99	✔	C
**YK0232**	PW	600	98	98	✔	B
**YK0233**	AcW	545	99	99	✔	C
**YK0234**	AcGW	602	100	100	✔	C
**YK0235**	AcEW	674	100	100	✔	C
**YK0236**	AcKW	673	97	97	✔	B
**YK0237**	AcYW	708	97	97	✔	C
**YK0238**	AcLW	658	99	99	✔	C
**YK0239**	AcPW	642	98	98	✔	C
**YK0240**	S	404	99	99	✔	B
**YK0241**	GS	461	88	92		C
**YK0242**	ES	533	87	87		C
**YK0243**	KS	532	91	93		C
**YK0244**	YS	567	88	89		C
**YK0245**	LS	517	89	91		C
**YK0246**	PS	501	88	88		C
**YK0247**	AcS	446	94	96	✔	B
**YK0248**	AcGS	503	85	85		C
**YK0249**	AcES	575	85	85		C
**YK0250**	AcKS	574	88	87		C
**YK0251**	AcYS	609	99	98	✔	B
**YK0252**	AcLS	559	99	99	✔	B
**YK0253**	AcPS	543	87	89		C
**YK0254**	G	374	93	93		C
**YK0255**	M	448	97	98	✔	B
**YK0256**	GM	505	97	98	✔	B

**YK0257**	EM	577	96	97	✓	B
**YK0258**	KM	576	96	96	✓	B
**YK0259**	YM	611	98	94	✓	B
**YK0260**	LM	561	97	99	✓	B
**YK0261**	PM	545	97	98	✓	B
**YK0262**	Mo	464	99	99	✓	B
**YK0263**	GMo	521	99	99	✓	B
**YK0264**	EMo	593	97	97	✓	B
**YK0265**	KMo	592	95	94	✓	B
**YK0266**	YMo	627	97	99	✓	B
**YK0267**	LMo	577	97	97	✓	B
**YK0268**	PMo	561	99	99	✓	B
**YK0269**	AcM	490	86	94	✓	B
**YK0270**	AcGM	547	92	93	✓	B
**YK0271**	AcEM	619	93	95	✓	B
**YK0272**	AcKM	618	96	97	✓	B
**YK0273**	AcYM	653	96	98	✓	B
**YK0274**	AcLM	603	97	98	✓	B
**YK0275**	AcPM	587	95	98	✓	B
**YK0276**	AcMo	506	94	97	✓	B
**YK0277**	AcGMo	563	91	96	✓	B
**YK0278**	AcEMo	635	96	99	✓	B
**YK0279**	AcKMo	634	95	97	✓	B
**YK0280**	AcYMo	669	97	99	✓	B
**YK0281**	AcLMo	619	92	92	✓	B
**YK0282**	AcPMo	603	96	99	✓	B
**YK0283**	Q	445	95	97	✓	B
**YK0284**	GQ	502	99	99	✓	D
**YK0285**	EQ	574	85	89		B
**YK0286**	KQ	573	96	97	✓	D
**YK0287**	YQ	608	85	90		B
**YK0288**	LQ	558	94	99	✓	B
**YK0289**	PQ	542	98	99	✓	B
**YK0290**	AcQ	487	94	97	✓	B
**YK0291**	AcGQ	544	96	99	✓	D
**YK0292**	AcEQ	616	85	91		B
**YK0293**	AcKQ	615	86	91		B

**YK0294**	AcYQ	650	85	93		B
**YK0295**	AcLQ	600	85	89		B
**YK0296**	AcPQ	584	87	93		B
**YK0297**	N	431	98	99	✔	B
**YK0298**	GN	488	85	88		B
**YK0299**	EN	560	89	90		B
**YK0300**	KN	559	85	87		B
**YK0301**	YN	594	85	89		B
**YK0302**	LN	544	89	91		B
**YK0303**	PN	528	87	90		B
**YK0304**	AcN	473	97	99	✔	D
**YK0305**	AcGN	530	97	99	✔	D
**YK0306**	AcEN	602	85	93		B
**YK0307**	AcKN	601	98	98	✔	D
**YK0308**	AcYN	636	86	93		B
**YK0309**	AcLN	586	98	97	✔	B
**YK0310**	AcPN	570	86	96		B
**YK0311**	T	418	100	100	✔	D
**YK0312**	GT	475	94	96		D
**YK0313**	ET	547	95	96		D
**YK0314**	KT	546	94	95		D
**YK0315**	YT	581	91	93		D
**YK0316**	LT	531	93	98		D
**YK0317**	PT	515	94	96		D
**YK0318**	AcT	460	88	87		D
**YK0319**	AcGT	517	89	95		D
**YK0320**	AcET	589	95	96		D
**YK0321**	AcKT	588	93	94		D
**YK0322**	AcYT	623	93	94		D
**YK0323**	AcLT	573	100	100	✔	D
**YK0324**	AcPT	557	95	98		D
**YK0325**	I	430	93	92		D
**YK0326**	GI	487	90	90		D
**YK0327**	EI	559	96	97		D
**YK0328**	KI	558	92	91		D
**YK0329**	YI	593	90	95		D
**YK0330**	LI	543	96	97		D

**YK0331**	PI	527	93	91		D
**YK0332**	AcI	472	93	95		D
**YK0333**	AcGI	529	91	95		D
**YK0334**	AcEI	601	87	90		D
**YK0335**	AcKI	600	85	91		D
**YK0336**	AcYI	635	89	97		D
**YK0337**	AcLI	585	95	100		D
**YK0338**	AcPI	569	97	100		D
**YK0339**	V	416	89	85		D
**YK0340**	GV	473	95	93		D
**YK0341**	EV	545	98	98		D
**YK0342**	KV	544	96	96		D
**YK0343**	YV	579	91	96		D
**YK0344**	LV	529	98	98		D
**YK0345**	PV	513	100	100		D
**YK0346**	AcV	458	93	96		D
**YK0347**	AcGV	515	96	97		D
**YK0348**	AcEV	587	97	99		D
**YK0349**	AcKV	586	96	99		D
**YK0350**	AcYV	621	95	96		D
**YK0351**	AcLV	571	94	95		D
**YK0352**	AcPV	555	96	97		D
**YK0353**	D	432	100	100	✔	D
**YK0354**	GD	489	94	94		D
**YK0355**	ED	561	96	97		D
**YK0356**	KD	560	95	94		D
**YK0357**	YD	595	96	95		D
**YK0358**	LD	545	95	97		D
**YK0359**	PD	529	96	96		D
**YK0360**	AcD	474	90	97	✔	D
**YK0361**	AcGD	531	91	94		D
**YK0362**	AcED	603	93	96		D
**YK0363**	AcKD	602	95	97		D
**YK0364**	AcYD	637	91	94		D
**YK0365**	AcLD	587	93	95		D
**YK0366**	AcPD	571	95	99		D
**YK0367**	C	420	92	91		D

**YK0368**	GC	477	94	94		D
**YK0369**	EC	549	98	98		D
**YK0370**	KC	548	99	99		D
**YK0371**	YC	583	99	98		D
**YK0372**	LC	533	100	100		D
**YK0373**	PC	517	96	97		D
**YK0374**	AcC	462	88	93		D
**YK0375**	AcGC	519	90	96		D
**YK0376**	AcEC	591	94	95		D
**YK0377**	AcKC	590	97	99		D
**YK0378**	AcYC	625	87	97		D
**YK0379**	AcLC	575	87	91		D
**YK0380**	AcPC	559	91	96		D

Table S1. List of synthesized probes

Analytical method shows LC-MS condition used to check the purity of probes. Eluent C (0.1 % formic acid in H_2O) and eluent D (0.1 % formic acid in 80 % acetonitrile, 20 % H_2O) were used as follows.

Analytical method A: C/D = 95 / 5 → 5 / 95 in 20 min

Analytical method B: C/D = 95 / 5 → 5 / 95 in 17.5 min

Analytical method C: C/D = 99 / 1 for 5 min then → 5 / 95 in 15 min

Analytical method D: C/D = 95 / 5 → 5 / 95 in 15 min

[Supplementary note]

XMo-HMRG derivatives were obtained as by-products of the corresponding XM-HMRGs.

		Lung adenocarcinoma lysate									
		NO.1		NO.2		NO.3		NO.4		NO.5	
		T	N	T	N	T	N	T	N	T	N
-	G	0.2	0.0	0.0	0.0	0.0	0.0	0.0	0.3	0.9	0.0
G		0.9	0.3	1.4	0.5	1.1	0.8	2.2	3.0	7.1	0.6
E		0.0	0.0	0.0	0.0	0.2	0.1	0.3	0.4	0.6	0.0
K		0.6	0.0	0.2	0.1	0.3	0.1	0.1	0.1	1.3	0.0
Y		0.7	0.3	0.2	0.3	0.7	0.3	0.6	0.6	2.5	0.0
L		0.0	0.0	0.0	0.0	0.0	0.0	0.2	0.0	1.6	0.0
P		0.0	0.0	0.0	0.0	0.0	0.0	0.7	0.9	4.7	0.0
Ac		2.1	1.3	2.0	1.2	2.4	1.1	1.9	1.1	3.0	1.3
AcG		0.0	0.0	0.0	0.0	0.0	0.0	0.0	0.0	0.0	0.0
AcE		0.0	0.0	0.0	0.0	0.0	0.0	0.0	0.0	0.0	0.0
AcK		0.0	0.0	0.0	0.0	0.0	0.0	0.0	0.0	0.0	0.0
AcY		0.0	0.0	0.0	0.0	0.0	0.0	0.0	0.0	0.0	0.0
AcL		0.0	0.0	0.0	0.0	0.0	0.0	0.0	0.0	0.0	0.0
AcP		0.0	0.0	0.0	0.0	0.0	0.0	0.0	0.0	0.0	0.0
a		0.0	0.0	0.0	0.0	0.0	0.0	0.0	0.1	0.2	0.0
d		0.0	0.0	0.0	0.0	0.0	0.0	0.0	0.0	0.0	0.0
s		0.0	0.0	0.0	0.0	0.0	0.0	0.1	0.2	0.8	0.0
βAla		0.0	0.0	0.0	0.0	0.0	0.0	0.0	0.0	0.0	0.0
MeGly		0.0	0.0	0.0	0.0	0.0	0.0	0.0	0.0	0.0	0.0
-	A	31.8	25.8	26.3	23.0	32.2	19.8	17.3	25.1	38.4	12.6
G		3.9	1.8	4.3	1.6	4.3	2.3	5.7	5.2	15.0	1.5
E		1.8	0.5	0.0	0.7	0.8	0.1	0.0	0.6	2.0	0.0
K		13.6	8.1	10.0	6.2	11.9	4.1	4.6	4.7	20.8	1.3
Y		8.2	5.0	5.1	4.8	7.2	4.0	2.5	3.6	14.0	0.1
L		8.2	4.1	5.9	3.2	7.8	2.3	3.3	3.1	15.0	0.8
P		2.2	1.0	2.3	0.7	2.0	1.5	4.3	4.0	16.4	0.3
Ac		0.0	0.0	0.0	0.0	0.0	0.0	0.0	0.0	0.0	0.0
AcG		0.0	0.0	0.0	0.0	0.0	0.0	0.0	0.0	0.0	0.0
AcE		0.0	0.0	0.0	0.0	0.0	0.0	0.0	0.0	0.0	0.0
AcK		0.0	0.0	0.0	0.0	0.0	0.0	0.0	0.0	0.0	0.0
AcY		0.0	0.0	0.0	0.0	0.0	0.0	0.0	0.0	0.0	0.0
AcL		0.0	0.0	0.0	0.0	0.0	0.0	0.0	0.0	0.0	0.0
AcP		0.0	0.0	0.0	0.0	0.0	0.0	0.0	0.0	0.0	0.0
-	V	0.0	0.0	0.0	0.0	0.0	0.0	0.0	0.0	0.0	0.0
G		0.0	0.0	0.0	0.0	0.0	0.0	0.0	0.0	0.0	0.0
E		0.0	0.0	0.0	0.0	0.0	0.0	0.0	0.0	0.0	0.0
K		0.0	0.0	0.0	0.0	0.0	0.0	0.0	0.0	0.0	0.0
Y		0.0	0.0	0.0	0.4	0.0	0.0	0.0	0.0	0.0	0.0
L		0.0	0.0	0.0	0.0	0.2	0.0	0.0	0.0	0.0	0.0
P		0.0	0.0	0.0	0.0	0.0	0.0	0.0	0.0	0.0	0.0
Ac		0.0	0.0	0.0	0.0	0.0	0.0	0.0	0.0	0.0	0.0
AcG		0.0	0.0	0.0	0.0	0.0	0.0	0.0	0.0	0.0	0.0
AcE		0.0	0.0	0.0	0.0	0.0	0.0	0.0	0.0	0.0	0.0
AcK		0.0	0.0	0.0	0.0	0.0	0.0	0.0	0.0	0.0	0.0
AcY		0.0	0.0	0.0	0.0	0.0	0.0	0.0	0.0	0.0	0.0
AcL		0.0	0.0	0.0	0.0	0.0	0.0	0.0	0.0	0.0	0.0
AcP		0.0	0.0	0.0	0.0	0.0	0.0	0.0	0.0	0.0	0.0
	L	9.6	6.8	4.5	7.0	9.9	4.3	3.1	4.5	7.9	2.2
G		2.1	0.0	4.4	1.0	4.6	1.5	7.7	6.0	19.9	0.7
E		1.5	1.2	0.8	0.8	2.1	0.6	1.0	1.1	1.8	0.3
K		10.7	7.3	7.6	6.1	11.2	3.5	4.1	4.5	12.3	2.8
Y		5.7	2.5	3.9	4.0	9.5	2.4	1.6	2.1	9.1	0.1
L		8.3	5.0	7.2	4.9	11.7	3.8	3.7	4.3	12.5	3.2
P		1.8	0.0	2.9	1.0	3.9	0.9	5.6	4.4	17.0	0.0
Ac		0.1	0.0	0.0	0.0	0.0	0.0	0.0	0.0	0.2	0.0
AcG		0.0	0.0	0.0	0.0	0.0	0.0	0.0	0.0	0.0	0.0
AcE		0.0	0.0	0.0	0.0	0.0	0.0	0.0	0.0	0.0	0.0
AcK		0.5	0.4	0.1	0.4	0.4	0.3	0.3	0.1	0.5	0.0
AcY		0.0	0.0	0.0	0.0	0.0	0.0	0.0	0.0	0.0	0.0
AcL		0.0	0.0	0.0	0.0	0.0	0.0	0.0	0.0	0.0	0.0
AcP		0.1	0.1	0.0	0.0	0.0	0.1	0.0	0.0	0.0	0.0
a		0.0	0.0	0.0	0.0	0.0	0.0	0.0	0.0	0.0	0.0
d		0.0	0.0	0.0	0.0	0.0	0.0	0.0	0.0	0.0	0.0
s		0.0	0.0	0.0	0.0	0.0	0.0	0.1	0.0	1.2	0.0
βAla		0.0	0.0	0.0	0.0	0.0	0.0	0.0	0.0	1.5	0.0
MeGly		0.0	0.0	0.0	0.0	0.0	0.0	0.0	0.0	0.0	0.0

		Lung adenocarcinoma lysate									
		NO.1		NO.2		NO.3		NO.4		NO.5	
		T	N	T	N	T	N	T	N	T	N
-	I	0.8	0.0	0.0	1.9	3.5	0.0	0.0	1.7	1.0	0.0
G		0.0	0.0	0.0	0.0	0.0	0.0	0.0	0.0	0.0	0.0
E		0.0	0.0	0.0	0.0	0.0	0.0	0.0	0.0	0.0	0.0
K		2.5	1.1	0.2	1.6	4.7	0.1	1.2	1.8	3.2	0.0
Y		0.0	0.0	0.0	0.6	1.8	0.0	0.0	0.4	1.4	0.0
L		0.9	0.3	0.9	1.0	3.5	0.3	0.8	0.8	2.6	0.1
P		0.0	0.0	0.0	0.0	0.0	0.0	0.0	0.0	0.0	0.0
Ac		0.0	0.0	0.0	0.0	0.0	0.0	0.0	0.0	0.0	0.0
AcG		0.0	0.0	0.0	0.0	0.0	0.0	0.0	0.0	0.0	0.0
AcE		0.0	0.0	0.0	0.0	4.2	0.0	0.0	0.0	0.0	0.0
AcK		0.0	0.0	0.0	0.0	0.0	0.0	0.0	0.0	0.0	0.0
AcY		0.0	0.0	0.0	0.0	0.0	0.0	0.0	0.0	0.0	0.0
AcL		0.0	0.0	0.0	0.0	0.0	0.0	0.0	0.0	0.0	0.0
AcP		0.0	0.0	0.0	0.0	0.0	0.0	0.0	0.0	0.0	0.0
-	S	1.3	0.9	0.8	0.9	1.3	0.8	0.6	1.0	2.8	0.6
G		0.0	0.0	0.0	0.0	0.0	0.0	1.4	2.8	13.6	0.0
E		0.0	0.0	0.0	0.0	0.0	0.0	0.0	0.0	0.0	0.0
K		0.0	0.0	0.0	0.0	0.0	0.0	0.0	0.0	0.0	0.0
Y		0.0	0.0	0.0	0.0	0.0	0.0	0.0	0.0	0.0	0.0
L		0.0	0.0	0.0	0.0	0.0	0.0	0.0	0.0	0.0	0.0
P		1.4	0.5	2.8	0.8	2.0	1.6	5.5	5.3	20.1	0.9
Ac		2.9	2.3	2.2	1.9	3.3	1.9	2.5	1.4	5.0	1.9
AcG		0.0	0.0	0.0	0.0	0.0	0.0	0.0	0.0	0.0	0.0
AcE		0.0	0.0	0.0	0.0	0.0	0.0	0.0	0.0	0.0	0.0
AcK		0.0	0.0	0.0	0.0	0.0	0.0	0.0	0.0	0.0	0.0
AcY		0.0	0.0	0.0	0.0	0.0	0.0	0.0	0.0	0.0	0.0
AcL		0.0	0.0	0.0	0.0	0.0	0.0	0.0	0.0	0.0	0.0
AcP		0.0	0.0	0.0	0.0	0.0	0.0	0.0	0.0	0.0	0.0
-	T	1.6	1.1	1.2	1.2	1.5	1.0	0.8	0.8	4.4	0.6
G		2.9	1.1	6.5	1.4	4.2	3.3	13.0	12.8	38.8	2.4
E		0.0	0.0	0.0	0.0	0.0	0.0	0.0	0.0	0.0	0.0
K		0.0	0.0	0.0	0.0	0.0	0.0	0.0	0.0	0.3	0.0
Y		0.0	0.0	0.0	0.0	0.0	0.0	0.0	0.0	0.0	0.0
L		0.0	0.0	0.0	0.0	0.0	0.0	0.0	0.0	1.4	0.0
P		1.6	0.0	2.3	0.0	2.1	1.2	7.2	8.3	23.9	0.5
Ac		0.1	0.0	0.0	0.0	0.0	0.0	0.0	0.0	0.0	0.0
AcG		0.0	0.0	0.0	0.0	0.0	0.0	0.0	0.0	0.0	0.0
AcE		0.0	0.0	0.0	0.0	0.0	0.0	0.0	0.0	0.0	0.0
AcK		0.0	0.0	0.0	0.0	0.0	0.0	0.0	0.0	0.0	0.0
AcY		0.0	0.0	0.0	0.0	0.0	0.0	0.0	0.0	0.0	0.0
AcL		0.0	0.0	0.0	0.0	0.0	0.0	0.0	0.0	1.0	0.0
AcP		0.0	0.0	0.0	0.0	0.0	0.0	0.0	0.0	0.0	0.0
-	C	0.0	0.0	0.0	0.3	0.6	0.2	0.0	0.3	0.1	0.0
G		0.2	0.0	0.0	0.2	0.9	0.2	1.3	1.2	2.8	0.1
E		0.1	0.0	0.0	0.0	0.0	0.0	0.0	0.0	0.6	0.0
K		0.4	0.1	0.1	0.2	0.1	0.1	0.0	0.0	0.8	0.0
Y		0.4	0.1	0.1	0.3	0.3	0.1	0.0	0.2	1.2	0.0
L		0.0	0.0	0.0	0.1	0.0	0.0	0.0	0.0	0.8	0.0
P		0.2	0.0	0.0	0.2	0.1	0.1	0.8	0.7	3.0	0.0
Ac		0.0	0.0	0.0	0.0	0.0	0.0	0.0	0.0	0.0	0.0
AcG		0.0	0.0	0.0	0.0	0.0	0.0	0.0	0.0	0.0	0.0
AcE		0.0	0.0	0.0	0.0	0.0	0.0	0.0	0.0	0.0	0.0
AcK		0.0	0.0	0.0	0.0	0.0	0.0	0.0	0.0	0.0	0.0
AcY		0.0	0.0	0.0	0.0	0.0	0.0	0.0	0.0	0.0	0.0
AcL		0.0	0.0	0.0	0.0	0.0	0.0	0.0	0.0	0.0	0.0
AcP		0.0	0.0	0.0	0.0	0.0	0.0	0.0	0.0	0.0	0.0
-	M	17.1	12.7	11.8	11.0	17.7	11.9	8.4	10.6	18.3	5.8
G		8.1	5.3	10.3	5.2	12.8	8.7	16.6	19.8	34.6	5.2
E		2.6	1.8	1.1	1.5	3.6	1.3	1.8	1.4	3.9	0.6
K		15.9	9.3	13.0	7.4	13.5	4.9	7.0	8.3	21.2	3.3
Y		7.2	5.0	5.0	5.9	11.2	4.1	2.9	4.1	15.5	1.4
L		8.7	4.7	7.6	4.3	9.5	3.3	3.5	4.4	15.6	2.3
P		7.3	2.4	8.9	4.7	8.5	6.6	13.5	14.5	41.3	3.7
Ac		0.6	0.3	0.4	0.3	0.7	0.3	0.4	0.4	0.8	0.5
AcG		0.0	0.0	0.0	0.0	0.0	0.0	0.0	0.0	0.0	0.0
AcE		0.0	0.0	0.0	0.0	0.0	0.0	0.0	0.0	0.0	0.0
AcK		0.2	0.1	0.0	0.1	0.1	0.1	0.0	0.1	0.5	0.0
AcY		0.1	0.0	0.0	0.1	0.1	0.0	0.0	0.1	0.5	0.0
AcL		0.3	0.0	0.2	0.1	0.1	0.0	0.1	0.1	2.2	0.0
AcP		0.0	0.0	0.0	0.0	0.0	0.0	0.0	0.0	0.1	0.0

		Lung adenocarcinoma lysate									
		NO.1		NO.2		NO.3		NO.4		NO.5	
		T	N	T	N	T	N	T	N	T	N
-	Mo	4.1	2.6	2.7	2.0	3.6	2.0	1.9	2.5	6.9	1.4
G		4.2	1.3	8.4	2.1	4.8	4.4	12.5	13.2	31.1	2.5
E		0.0	0.0	0.0	0.1	0.1	0.0	0.0	0.0	0.1	0.0
K		1.3	0.7	0.9	0.7	1.1	0.3	0.4	0.5	2.0	0.1
Y		0.8	0.2	0.0	0.0	0.1	0.0	0.0	0.0	1.3	0.0
L		0.3	0.0	0.0	0.0	0.0	0.0	0.0	0.0	3.1	0.0
P		2.5	1.2	3.4	1.4	3.8	2.9	9.8	9.1	24.0	2.1
Ac		0.0	0.0	0.0	0.0	0.0	0.0	0.0	0.0	0.0	0.0
AcG		0.0	0.0	0.0	0.0	0.0	0.0	0.0	0.0	0.0	0.0
AcE		0.0	0.0	0.0	0.0	0.0	0.0	0.0	0.0	0.0	0.0
AcK		0.0	0.0	0.0	0.0	0.0	0.0	0.0	0.0	0.0	0.0
AcY		0.0	0.0	0.0	0.0	0.0	0.0	0.0	0.0	0.2	0.0
AcL		0.0	0.0	0.0	0.0	0.0	0.0	0.0	0.0	0.0	0.0
AcP		0.0	0.0	0.0	0.0	0.0	0.0	0.0	0.0	0.0	0.0
-	F	2.1	1.1	0.4	1.1	2.1	0.6	0.0	0.7	2.2	0.1
G		2.1	0.2	2.1	1.0	3.0	1.2	3.9	2.5	12.4	0.6
E		0.3	0.0	0.0	0.1	0.0	0.0	0.1	0.0	0.3	0.0
K		3.0	0.8	0.7	0.6	2.5	0.0	0.4	0.1	2.5	0.0
Y		1.0	0.8	0.4	0.7	2.7	0.4	0.3	0.3	2.1	0.6
L		1.3	0.5	0.8	0.8	2.8	0.2	0.6	0.4	2.7	0.0
P		0.8	0.3	0.7	0.7	2.4	0.6	3.1	3.5	9.3	0.5
Ac		0.3	0.4	0.7	0.3	1.0	0.2	0.8	0.3	1.3	0.7
AcG		0.0	0.0	0.0	0.0	0.0	0.0	0.0	0.0	0.0	0.0
AcE		0.0	0.0	0.0	0.0	0.0	0.0	0.0	0.0	0.0	0.0
AcK		0.0	0.0	0.0	0.0	0.0	0.0	0.0	0.0	0.0	0.0
AcY		0.0	0.0	0.0	0.0	0.0	0.0	0.0	0.0	0.0	0.0
AcL		0.0	0.0	0.0	0.0	0.0	0.0	0.0	0.0	0.0	0.0
AcP		0.0	0.0	0.0	0.0	0.0	0.0	0.0	0.0	0.0	0.0
	Y	5.2	3.1	3.8	2.7	4.7	2.6	2.0	2.4	6.4	1.5
G		10.1	4.1	11.9	4.0	11.5	6.6	17.5	13.6	39.8	4.5
E		0.7	0.4	0.5	0.2	0.8	0.2	0.3	0.6	1.6	0.0
K		3.6	1.6	2.8	1.0	3.1	0.8	1.4	1.0	6.1	0.3
Y		3.5	1.5	2.9	1.3	3.8	1.4	1.5	1.8	6.9	1.2
L		2.9	0.7	2.2	0.9	3.4	0.7	1.1	0.9	5.6	0.6
P		2.2	0.8	3.1	1.0	3.1	1.9	5.5	5.4	15.1	1.3
Ac		0.0	0.0	0.0	0.0	0.0	0.0	0.0	0.0	0.0	0.0
AcG		0.0	0.0	0.0	0.0	0.0	0.0	0.0	0.0	0.0	0.0
AcE		0.0	0.0	0.0	0.0	0.0	0.0	0.0	0.0	0.0	0.0
AcK		0.0	0.0	0.0	0.0	0.0	0.0	0.0	0.0	0.0	0.0
AcY		0.0	0.0	0.0	0.0	0.0	0.0	0.0	0.0	0.0	0.0
AcL		0.0	0.0	0.0	0.0	0.0	0.0	0.0	0.0	0.0	0.0
AcP		0.0	0.0	0.0	0.0	0.0	0.0	0.0	0.0	0.0	0.0
a		0.0	0.0	0.0	0.0	0.0	0.0	0.2	0.0	1.5	0.0
d		0.0	0.0	0.0	0.0	0.0	0.0	0.0	0.0	0.0	0.0
s		0.0	0.0	0.0	0.0	0.0	0.0	0.1	0.0	1.4	0.0
βAla		0.0	0.0	0.0	0.0	0.0	0.0	0.0	0.0	0.0	0.0
MeGly		0.0	0.0	0.0	0.0	0.0	0.0	0.1	0.0	2.8	0.0
-	W	1.3	0.6	1.0	0.6	1.6	0.7	0.4	0.5	1.7	0.4
G		1.4	0.5	2.4	0.7	3.2	1.1	4.9	3.8	12.2	1.7
E		0.0	0.0	0.0	0.0	0.1	0.0	0.0	0.0	0.4	0.0
K		1.2	0.5	0.7	0.5	1.8	0.3	0.5	0.6	2.1	0.2
Y		0.1	0.0	0.0	0.1	0.9	0.1	0.0	0.3	1.0	0.0
L		0.5	0.3	0.4	0.3	1.2	0.3	0.3	0.4	1.7	0.0
P		0.3	0.0	1.5	0.1	1.4	0.6	2.9	2.3	11.1	0.6
Ac		0.0	0.0	0.0	0.0	0.0	0.0	0.0	0.0	0.0	0.0
AcG		0.0	0.0	0.0	0.0	0.0	0.0	0.0	0.0	0.0	0.0
AcE		0.1	0.0	0.0	0.0	0.0	0.0	0.0	0.0	0.0	0.0
AcK		0.0	0.0	0.0	0.0	0.0	0.0	0.0	0.0	0.0	0.0
AcY		0.0	0.0	0.0	0.0	0.1	0.0	0.0	0.0	0.0	0.0
AcL		0.0	0.0	0.0	0.0	0.0	0.0	0.0	0.0	0.2	0.0
AcP		0.0	0.0	0.0	0.0	0.0	0.0	0.0	0.0	0.0	0.0

		Lung adenocarcinoma lysate									
		NO.1		NO.2		NO.3		NO.4		NO.5	
		T	N	T	N	T	N	T	N	T	N
	P	0.2	0.2	0.2	0.2	0.5	0.2	0.1	0.2	0.4	0.0
G		17.5	7.4	20.8	8.3	11.4	10.8	11.6	8.5	43.8	5.4
E		14.6	6.5	17.4	6.1	9.8	9.2	10.7	6.4	45.9	5.5
K		36.2	20.6	34.0	15.3	26.6	17.3	23.7	17.1	63.6	13.3
Y		32.3	11.9	34.6	12.2	22.5	16.8	18.7	12.2	71.2	11.0
L		28.4	12.9	28.0	9.8	20.9	14.2	18.2	12.4	61.9	8.8
P		30.0	11.7	34.1	10.4	18.3	13.2	18.1	11.1	65.9	7.4
Ac		0.0	0.0	0.0	0.0	0.0	0.0	0.0	0.0	0.0	0.0
AcG		0.9	0.2	0.4	0.2	0.7	0.0	0.3	0.2	0.4	0.0
AcE		0.6	0.1	0.4	0.1	1.0	0.0	0.3	0.0	0.4	0.0
AcK		6.6	3.0	5.5	2.5	9.1	2.4	3.9	2.7	4.9	1.4
AcY		2.6	1.1	2.7	1.0	5.2	0.9	1.8	0.8	2.9	0.5
AcL		2.4	0.8	2.0	0.9	4.8	1.0	1.8	1.0	3.2	0.8
AcP		7.6	3.0	5.7	2.7	11.4	2.6	5.0	2.6	6.8	1.9
a		1.8	1.4	1.4	2.0	2.4	1.9	2.2	1.6	2.8	1.6
d		0.0	0.0	0.0	0.0	0.0	0.0	0.0	0.0	0.0	0.0
s		3.0	1.5	1.4	2.4	2.9	1.8	3.1	0.9	3.6	0.3
β Ala		0.1	0.0	0.0	0.0	0.0	0.0	0.0	0.0	1.2	0.0
MeGly		12.7	4.1	14.7	4.2	7.7	5.9	7.7	4.3	44.6	3.2
-	N	0.5	0.4	0.4	0.6	0.7	0.4	0.3	0.4	1.0	0.2
G		0.0	0.0	0.0	0.0	0.0	0.0	1.9	1.3	9.9	0.0
E		0.0	0.0	0.0	0.0	0.0	0.0	0.0	0.0	0.0	0.0
K		0.0	0.0	0.0	0.0	0.0	0.0	0.0	0.0	0.0	0.0
Y		0.0	0.0	0.0	0.0	0.0	0.0	0.0	0.0	0.0	0.0
L		0.0	0.0	0.0	0.0	0.0	0.0	0.0	0.0	0.0	0.0
P		0.0	0.0	0.0	0.0	0.0	0.0	0.8	0.5	7.2	0.0
Ac		0.2	0.1	0.1	0.1	0.2	0.0	0.0	0.0	0.2	0.1
AcG		0.0	0.0	0.0	0.0	0.0	0.0	0.0	0.0	0.0	0.0
AcE		0.0	0.0	0.0	0.0	0.0	0.0	0.0	0.0	0.0	0.0
AcK		0.0	0.0	0.0	0.0	0.0	0.0	0.0	0.0	0.1	0.0
AcY		0.0	0.0	0.0	0.0	0.0	0.0	0.0	0.0	0.0	0.0
AcL		0.0	0.0	0.0	0.0	0.0	0.0	0.0	0.0	0.3	0.0
AcP		0.0	0.0	0.0	0.0	0.0	0.0	0.0	0.0	0.0	0.0
-	Q	4.5	3.0	3.2	2.2	3.9	2.2	2.3	3.8	8.2	1.7
G		3.4	1.7	6.2	1.7	5.2	3.2	12.8	14.1	30.2	2.6
E		0.0	0.0	0.0	0.0	0.0	0.0	0.0	0.0	0.0	0.0
K		1.7	0.8	0.8	0.5	1.1	0.3	0.4	0.6	2.2	0.0
Y		0.0	0.0	0.0	0.0	0.0	0.0	0.0	0.0	0.0	0.0
L		0.0	0.0	0.0	0.0	0.0	0.0	0.0	0.0	1.9	0.0
P		4.3	1.6	8.1	2.3	5.9	4.4	16.3	13.5	39.8	3.5
Ac		0.0	0.0	0.0	0.0	0.0	0.0	0.0	0.0	0.0	0.0
AcG		0.0	0.0	0.0	0.0	0.0	0.0	0.0	0.0	0.0	0.0
AcE		0.0	0.0	0.0	0.0	0.0	0.0	0.0	0.0	0.0	0.0
AcK		0.0	0.0	0.0	0.0	0.0	0.0	0.0	0.0	0.0	0.0
AcY		0.0	0.0	0.0	0.0	0.0	0.0	0.0	0.0	0.0	0.0
AcL		0.0	0.0	0.0	0.0	0.0	0.0	0.0	0.0	0.0	0.0
AcP		0.0	0.0	0.0	0.0	0.0	0.0	0.0	0.0	0.0	0.0
-	D	0.4	0.5	0.2	0.3	0.5	0.2	0.5	0.4	0.4	0.3
G		0.0	0.0	0.0	0.0	0.0	0.0	0.0	0.0	0.0	0.0
E		0.0	0.0	0.0	0.0	0.0	0.0	0.0	0.0	0.0	0.0
K		0.0	0.0	0.0	0.0	0.0	0.0	0.0	0.0	0.0	0.0
Y		0.0	0.0	0.0	0.0	0.0	0.0	0.0	0.0	0.0	0.0
L		0.0	0.0	0.0	0.0	0.0	0.0	0.0	0.0	0.0	0.0
P		0.0	0.0	0.0	0.0	0.0	0.0	0.0	0.0	0.0	0.0
Ac		0.0	0.0	0.0	0.0	0.0	0.0	0.0	0.0	0.0	0.0
AcG		0.0	0.0	0.0	0.0	0.0	0.0	0.0	0.0	0.0	0.0
AcE		0.0	0.0	0.0	0.0	0.0	0.0	0.0	0.0	0.0	0.0
AcK		0.0	0.0	0.0	0.0	0.0	0.0	0.0	0.0	0.0	0.0
AcY		0.0	0.0	0.0	0.0	0.0	0.0	0.0	0.0	0.0	0.0
AcL		0.0	0.0	0.0	0.0	0.0	0.0	0.0	0.0	0.0	0.0
AcP		0.0	0.0	0.0	0.0	0.0	0.0	0.0	0.0	0.0	0.0

		Lung adenocarcinoma lysate									
		NO.1		NO.2		NO.3		NO.4		NO.5	
		T	N	T	N	T	N	T	N	T	N
-	E	1.2	2.2	0.5	1.1	0.7	0.7	1.6	1.4	1.3	0.9
G		0.0	0.0	0.0	0.0	0.0	0.0	0.8	0.6	3.4	0.0
E		0.0	0.0	0.0	0.0	0.0	0.0	0.0	0.0	0.0	0.0
K		0.0	0.0	0.0	0.0	0.0	0.0	0.0	0.0	0.0	0.0
Y		0.0	0.0	0.0	0.0	0.0	0.0	0.0	0.0	0.0	0.0
L		1.2	0.0	0.0	0.0	0.9	0.0	0.0	0.0	1.1	0.0
P		0.0	0.0	0.0	0.0	0.0	0.0	0.0	0.0	0.5	0.0
Ac		0.0	0.0	0.0	0.0	0.0	0.0	0.0	0.0	0.0	0.0
AcG		0.0	0.0	0.0	0.0	0.0	0.0	0.0	0.0	0.0	0.0
AcE		0.0	0.0	0.0	0.0	0.0	0.0	0.0	0.0	0.0	0.0
AcK		0.0	0.0	0.0	0.0	0.0	0.0	0.0	0.0	0.0	0.0
AcY		0.0	0.0	0.0	0.0	0.0	0.0	0.0	0.0	0.0	0.0
AcL		0.0	0.0	0.0	0.0	0.0	0.0	0.0	0.0	0.0	0.0
AcP		0.0	0.0	0.0	0.0	0.0	0.0	0.0	0.0	0.0	0.0
a		0.0	0.0	0.0	0.0	0.0	0.0	0.0	0.0	0.0	0.0
d		0.0	0.0	0.0	0.0	0.0	0.0	0.0	0.0	0.0	0.0
s		0.0	0.0	0.0	0.0	0.0	0.0	0.0	0.0	0.0	0.0
βAla		0.0	0.0	0.0	0.0	0.0	0.0	0.0	0.0	0.0	0.0
MeGly		0.0	0.0	0.0	0.0	0.0	0.0	0.0	0.0	0.0	0.0
-	K	22.3	17.6	20.6	15.9	22.6	12.4	11.0	14.2	28.5	9.2
G		30.8	17.0	42.5	19.6	38.8	28.7	63.8	61.9	51.6	19.9
E		2.9	2.8	1.7	1.9	2.8	1.4	1.6	1.8	2.7	0.6
K		19.6	14.6	17.8	11.2	17.6	7.3	8.6	8.9	25.1	6.3
Y		14.7	12.2	13.7	10.5	15.6	8.0	7.7	8.9	19.4	5.0
L		9.6	4.1	6.9	4.4	9.2	1.8	2.1	1.7	14.6	0.0
P		17.4	12.7	30.9	12.7	24.2	20.9	44.9	45.1	42.6	16.1
Ac		0.9	1.1	0.3	0.7	0.1	1.1	0.0	0.8	0.9	0.6
AcG		3.4	6.6	0.0	3.5	0.0	6.0	0.2	4.9	5.0	3.6
AcE		2.8	4.3	0.0	2.9	0.0	3.8	0.0	2.5	2.9	0.8
AcK		25.4	32.9	20.4	30.9	17.4	35.4	19.1	36.5	34.9	31.6
AcY		1.8	2.0	0.0	2.3	0.0	2.0	0.0	1.2	2.0	0.6
AcL		2.5	4.2	0.0	2.8	0.0	6.4	0.0	3.2	5.5	3.8
AcP		13.9	19.9	6.7	12.9	4.0	19.7	2.8	16.3	19.3	14.0
a		0.0	0.0	0.0	0.1	0.0	0.0	4.4	3.5	12.2	0.0
d		0.0	0.0	0.0	0.0	0.0	0.0	0.0	0.0	0.0	0.0
s		2.3	2.1	3.7	1.8	2.9	3.2	6.0	6.3	17.4	2.4
βAla		0.0	0.0	0.0	0.0	0.0	0.0	0.0	0.0	0.0	0.0
MeGly		0.9	0.0	3.0	0.2	2.1	1.5	9.1	9.8	26.1	0.1
-	R	29.5	24.1	30.6	21.5	30.3	18.1	17.4	19.8	35.0	12.4
G		27.6	12.2	44.3	13.4	33.0	22.7	53.1	42.7	37.9	19.0
E		5.6	5.4	3.0	3.3	5.6	2.8	3.3	3.9	5.0	2.1
K		18.8	9.8	15.0	6.8	12.3	4.9	5.9	5.7	20.6	3.8
Y		20.2	15.8	18.9	13.7	21.0	10.0	10.1	11.8	20.9	2.5
L		23.7	14.7	19.3	11.5	22.9	9.0	11.9	12.3	31.8	7.7
P		22.3	12.5	32.8	13.8	30.6	25.7	43.9	49.9	39.6	20.5
Ac		1.5	2.5	0.9	1.8	1.0	2.5	0.5	1.9	2.0	1.3
AcG		14.4	22.2	9.7	17.4	8.9	28.0	7.7	24.4	19.7	18.2
AcE		20.2	35.5	16.7	22.8	10.6	35.8	12.5	29.9	34.1	32.5
AcK		44.8	51.2	49.1	55.6	40.0	57.5	40.2	54.0	59.8	54.1
AcY		6.2	8.9	6.0	6.4	3.7	10.7	3.2	8.1	11.2	10.9
AcL		10.2	13.0	7.0	9.4	5.4	13.5	4.4	11.0	21.3	17.0
AcP		28.5	32.5	26.6	29.3	19.8	33.9	18.5	26.7	41.3	37.2
-	H	0.7	0.3	0.4	0.3	0.4	0.2	0.0	0.3	2.2	0.0
G		3.8	1.2	6.3	2.0	5.4	4.1	11.8	10.1	28.6	3.0
E		0.0	0.0	0.0	0.0	0.0	0.0	0.0	0.0	0.0	0.0
K		0.8	0.0	0.0	0.2	0.1	0.0	0.1	0.0	1.7	0.0
Y		0.8	0.1	0.5	0.3	0.7	0.4	0.4	0.5	2.9	0.2
L		0.5	0.0	0.3	0.0	0.3	0.0	0.2	0.2	2.8	0.0
P		3.0	0.9	4.7	1.4	3.7	2.9	8.5	6.9	26.0	2.8
Ac		0.0	0.0	0.0	0.0	0.0	0.0	0.0	0.0	0.0	0.0
AcG		0.0	0.0	0.0	0.0	0.0	0.0	0.0	0.0	0.0	0.0
AcE		0.0	0.0	0.0	0.0	0.0	0.0	0.0	0.0	0.0	0.0
AcK		0.0	0.0	0.0	0.0	0.0	0.0	0.0	0.0	0.0	0.0
AcY		0.0	0.0	0.0	0.0	0.0	0.0	0.0	0.0	0.0	0.0
AcL		0.1	0.0	0.0	0.0	0.0	0.0	0.0	0.0	0.8	0.0
AcP		0.0	0.0	0.0	0.0	0.0	0.0	0.0	0.0	0.0	0.0

		Lung adenocarcinoma lysate									
		NO.1		NO.2		NO.3		NO.4		NO.5	
		T	N	T	N	T	N	T	N	T	N
G	a	0.0	0.0	0.0	0.0	0.0	0.0	0.0	0.0	0.0	0.0
E		0.0	0.0	0.0	0.0	0.0	0.0	0.0	0.0	0.0	0.0
K		0.0	0.0	0.0	0.0	0.0	0.0	0.0	0.0	0.0	0.0
Y		0.0	0.0	0.0	0.0	0.0	0.0	0.0	0.0	0.0	0.0
L		0.0	0.0	0.0	0.0	0.0	0.0	0.0	0.0	0.0	0.0
P		0.0	0.0	0.0	0.0	0.0	0.0	0.0	0.0	0.0	0.0
a		0.0	0.0	0.0	0.0	0.0	0.0	0.0	0.0	0.0	0.0
d		0.0	0.0	0.0	0.0	0.0	0.0	0.0	0.0	0.0	0.0
s		0.0	0.0	0.0	0.0	0.0	0.0	0.0	0.0	0.0	0.0
βAla		0.0	0.0	0.0	0.0	0.0	0.0	0.0	0.0	0.0	0.0
MeGly		0.0	0.0	0.0	0.0	0.0	0.0	0.0	0.0	0.0	0.0
G	d	0.0	0.0	0.0	0.0	0.0	0.0	0.0	0.0	0.0	0.0
E		0.0	0.0	0.0	0.0	0.0	0.0	0.0	0.0	0.0	0.0
K		0.0	0.0	0.0	0.0	0.0	0.0	0.0	0.0	0.0	0.0
Y		0.0	0.0	0.0	0.0	0.0	0.0	0.0	0.0	0.0	0.0
L		0.0	0.0	0.0	0.0	0.0	0.0	0.0	0.0	0.0	0.0
P		0.0	0.0	0.0	0.0	0.0	0.0	0.0	0.0	0.0	0.0
a		0.0	0.0	0.0	0.0	0.0	0.0	0.0	0.0	0.0	0.0
d		0.0	0.0	0.0	0.0	0.0	0.0	0.0	0.0	0.0	0.0
s		0.0	0.0	0.0	0.0	0.0	0.0	0.0	0.0	0.0	0.0
βAla		0.0	0.0	0.0	0.0	0.0	0.0	0.0	0.0	0.0	0.0
MeGly		0.0	0.0	0.0	0.0	0.0	0.0	0.0	0.0	0.0	0.0
G	s	0.0	0.0	0.0	0.0	0.0	0.0	0.0	0.0	0.0	0.0
E		0.0	0.0	0.0	0.0	0.0	0.0	0.0	0.0	0.0	0.0
K		0.0	0.0	0.0	0.0	0.0	0.0	0.0	0.0	0.0	0.0
Y		0.0	0.0	0.0	0.0	0.0	0.0	0.0	0.0	0.0	0.0
L		0.0	0.0	0.0	0.0	0.0	0.0	0.0	0.0	0.0	0.0
P		0.0	0.0	0.0	0.0	0.0	0.0	0.0	0.0	0.0	0.0
a		0.0	0.0	0.0	0.0	0.0	0.0	0.0	0.0	0.0	0.0
d		0.0	0.0	0.0	0.0	0.0	0.0	0.0	0.0	0.0	0.0
s		0.0	0.0	0.0	0.0	0.0	0.0	0.0	0.0	0.0	0.0
βAla		0.0	0.0	0.0	0.0	0.0	0.0	0.0	0.0	0.0	0.0
MeGly		0.0	0.0	0.0	0.0	0.0	0.0	0.0	0.0	0.0	0.0
G	βAla	0.0	0.0	0.0	0.0	0.0	0.0	0.0	0.0	0.0	0.0
E		0.0	0.0	0.0	0.0	0.0	0.0	0.0	0.0	0.0	0.0
K		0.0	0.0	0.0	0.0	0.0	0.0	0.0	0.0	0.0	0.0
Y		0.0	0.0	0.0	0.0	0.0	0.0	0.0	0.0	0.0	0.0
L		0.0	0.0	0.0	0.0	0.0	0.0	0.0	0.0	0.0	0.0
P		0.0	0.0	0.0	0.0	0.0	0.0	0.0	0.0	0.0	0.0
a		0.0	0.0	0.0	0.0	0.0	0.0	0.0	0.0	0.0	0.0
d		0.0	0.0	0.0	0.0	0.0	0.0	0.0	0.0	0.0	0.0
s		0.0	0.0	0.0	0.0	0.0	0.0	0.0	0.0	0.0	0.0
βAla		0.0	0.0	0.0	0.0	0.0	0.0	0.0	0.0	0.0	0.0
MeGly		0.0	0.0	0.0	0.0	0.0	0.0	0.0	0.0	0.0	0.0
-	MeGly	0.2	0.0	0.1	0.0	0.3	0.0	0.1	0.0	0.1	0.0
G		1.8	1.5	0.8	2.3	1.9	1.6	1.9	1.7	2.5	1.1
E		2.7	0.0	1.4	0.9	2.1	0.0	1.9	0.0	11.3	0.0
K		26.2	19.1	27.7	20.3	24.4	21.9	24.6	20.7	65.3	17.3
Y		4.4	0.0	3.2	2.1	2.7	2.7	3.4	0.2	18.7	0.0
L		7.3	3.4	6.8	3.4	5.4	5.2	4.8	4.6	23.8	4.8
P		4.2	0.7	5.8	1.8	2.0	1.4	3.3	0.3	23.4	0.0
a		16.1	16.2	13.9	16.6	17.6	19.4	18.9	18.8	22.7	21.3
d		0.0	0.0	0.0	0.0	0.0	0.0	0.0	0.0	0.0	0.0
s		9.3	8.8	6.7	8.9	8.9	9.4	7.7	12.2	12.6	11.5
βAla		0.0	0.0	0.0	0.0	0.0	0.0	0.0	0.0	0.0	0.0
MeGly		1.5	0.6	0.7	1.0	1.3	1.0	1.3	0.9	2.4	1.0

Figure S2. The results of screening with tissue lysate from 5 adenocarcinoma patients.

The conversion rate of 1 μM probes in the library after incubation with 500 ng tissue lysate for 1 h is listed. All assays were carried out at 37 °C in 20 μL total volume of phosphate-buffered saline (pH 7.4) equipped with 100 mg/L $CaCl_2$ and $MgCl_2·6H_2O$ containing 0.5 % DMSO as a cosolvent (n=1). Excitation/emission wavelengths were 485/535 nm. Abbreviations: Mo; methionine sulfoxide, MeGly; *N*-methylglycine.

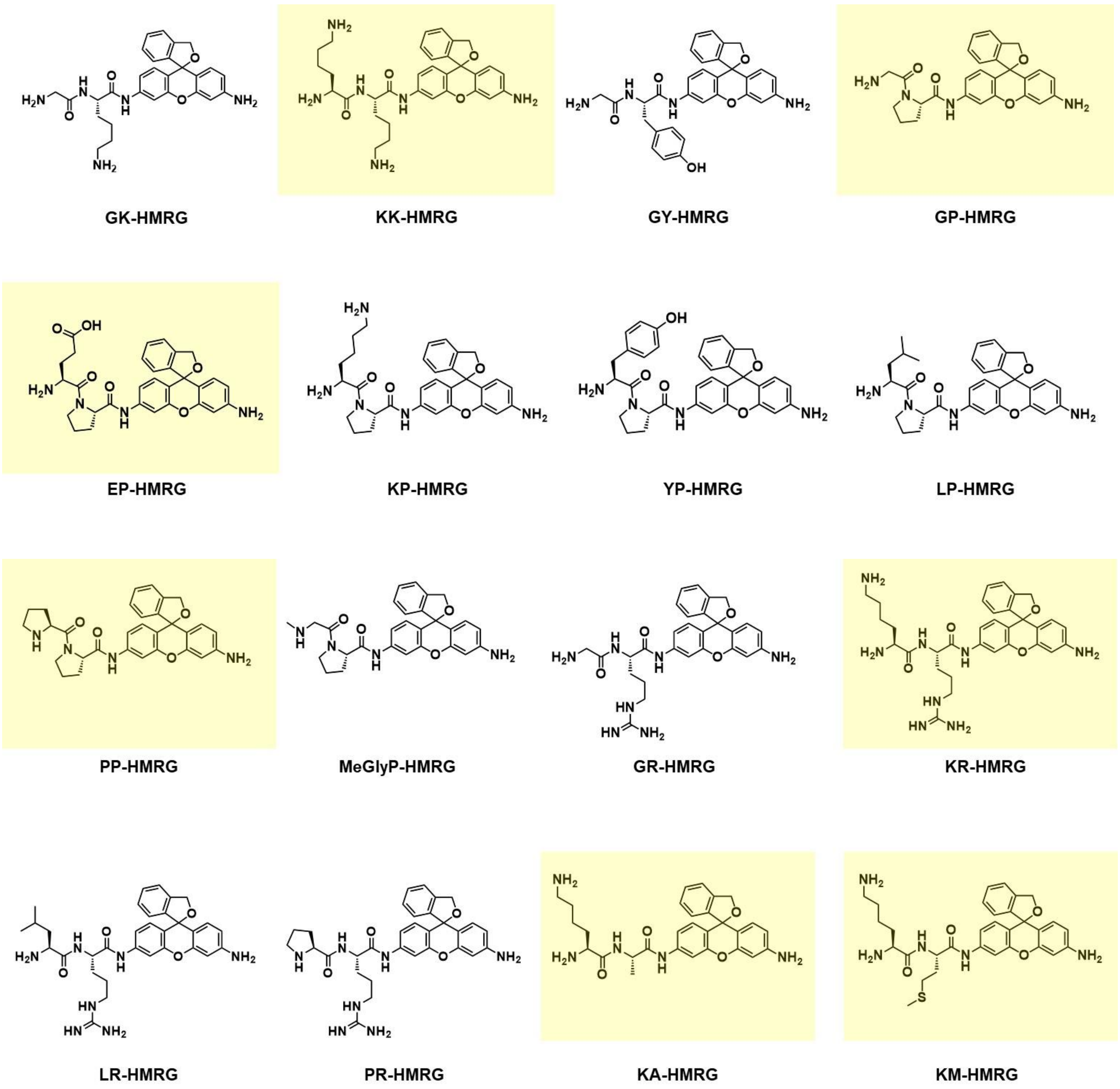

Figure S3. Chemical structures of the hit probes in the lysate screenings.

The 7 probes highlighted in yellow were selected for imaging-based screening.

Adenocarcinoma

Probe	15	32	33	37	187	201	258
n(non-tumor)	19	7	17	7	7	16	7
n(tumor)	19	7	17	7	7	16	7
Cut off value	0.420	0.918	0.683	0.792	0.297	0.433	0.214
Specificity	0.789	1.000	0.647	0.857	0.857	0.688	0.571
Sensitivity	0.789	0.714	0.765	0.857	0.857	0.875	1.000
AUC	0.842	0.918	0.747	0.837	0.857	0.840	0.796

Squamous cell carcinoma

Probe	15	32	33	37	187	201	258
n(non-tumor)	14	10	10	10	10	14	10
n(tumor)	14	10	10	10	10	14	10
Cut off value	0.662	0.352	1.190	0.862	1.064	0.403	0.246
Specificity	0.857	0.300	0.800	0.700	0.900	0.429	0.500
Sensitivity	0.786	1.000	0.600	0.600	0.500	0.929	0.900
AUC	0.857	0.560	0.630	0.640	0.680	0.694	0.690

All

Probe	15	32	33	37	187	201	258
n(non-tumor)	33	17	27	17	17	30	17
n(tumor)	33	17	27	17	17	30	17
Cut off value	0.532	1.001	1.190	0.792	0.377	0.433	0.246
Specificity	0.848	0.706	0.852	0.706	0.706	0.600	0.529
Sensitivity	0.727	0.647	0.556	0.706	0.765	0.833	0.882
AUC	0.842	0.678	0.705	0.737	0.744	0.773	0.716

Table S2. Results of imaging-based screening of the 7 probes in lung adenocarcinoma or squamous cell carcinoma.

(a)

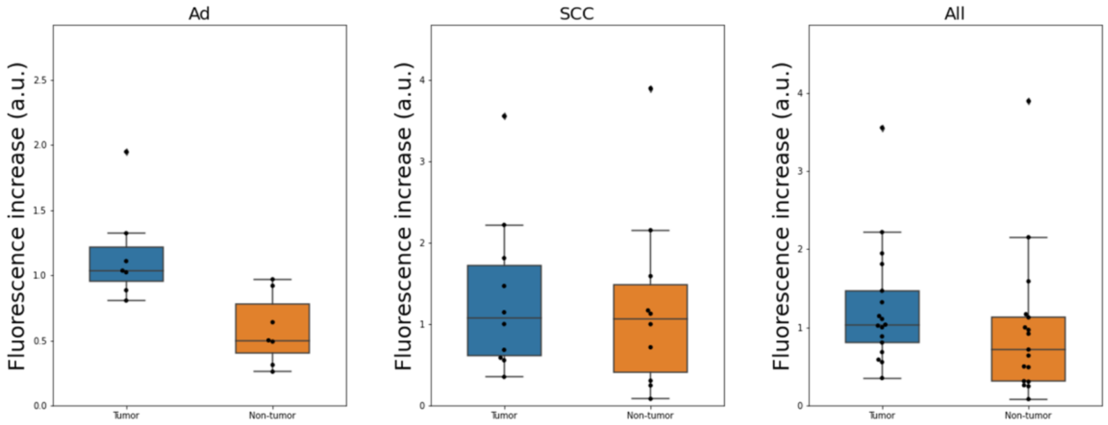

Ad
SCC
All
Fluorescence increase (a.u.)
Tumor
Non-tumor

(b)

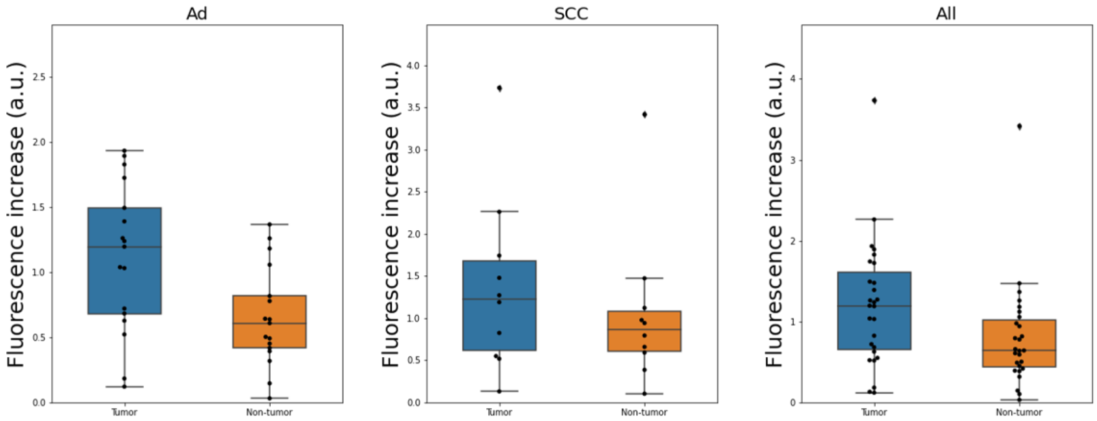

Ad
SCC
All
Fluorescence increase (a.u.)
Tumor
Non-tumor

(c)

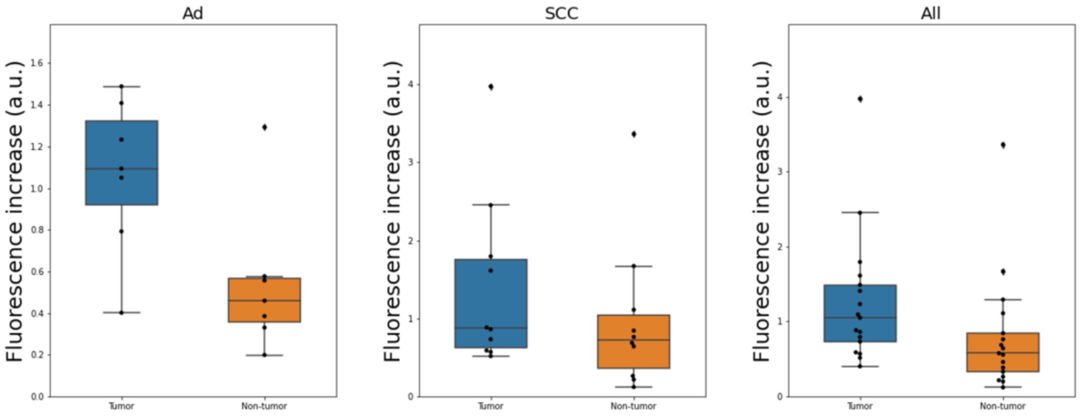

Ad
SCC
All
Fluorescence increase (a.u.)
Tumor
Non-tumor

(d)

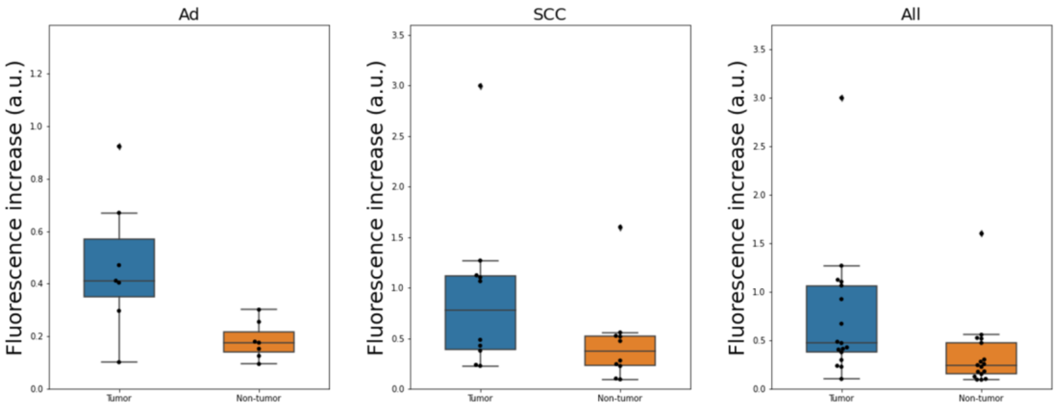

(e)

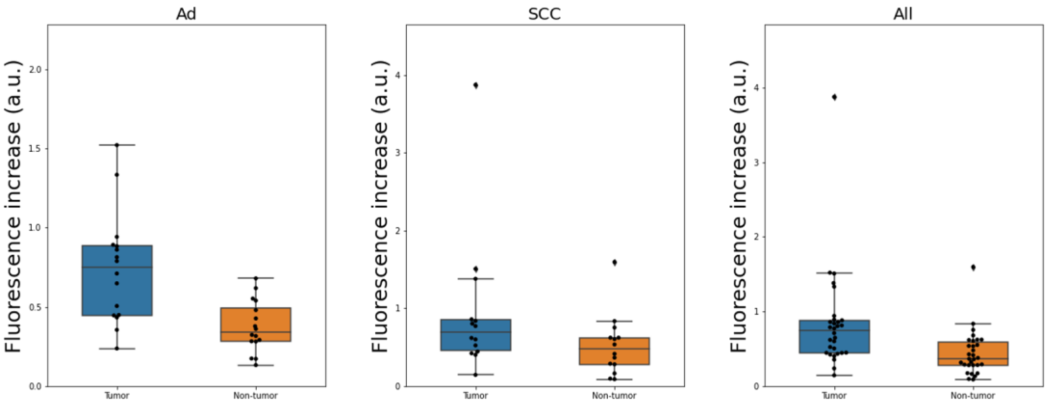

(f)

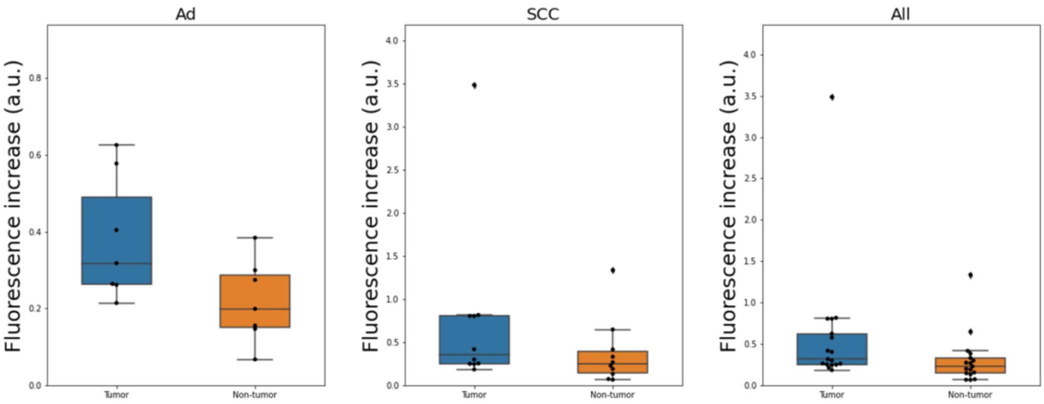

Figure S4. Fluorescence increase of (a) GP-, (b) EP-, (c) PP-, (d) KR-, (e) KA- and (f) KM-HMRG applied to lung adenocarcinoma or squamous cell carcinoma after 30 min. 50 µM probe solution in phosphate-buffered saline was applied to tumour or non-tumour tissues, and fluorescence images were acquired with a Maestro® imaging system. Excitation/emission wavelengths = 455/540 nm.

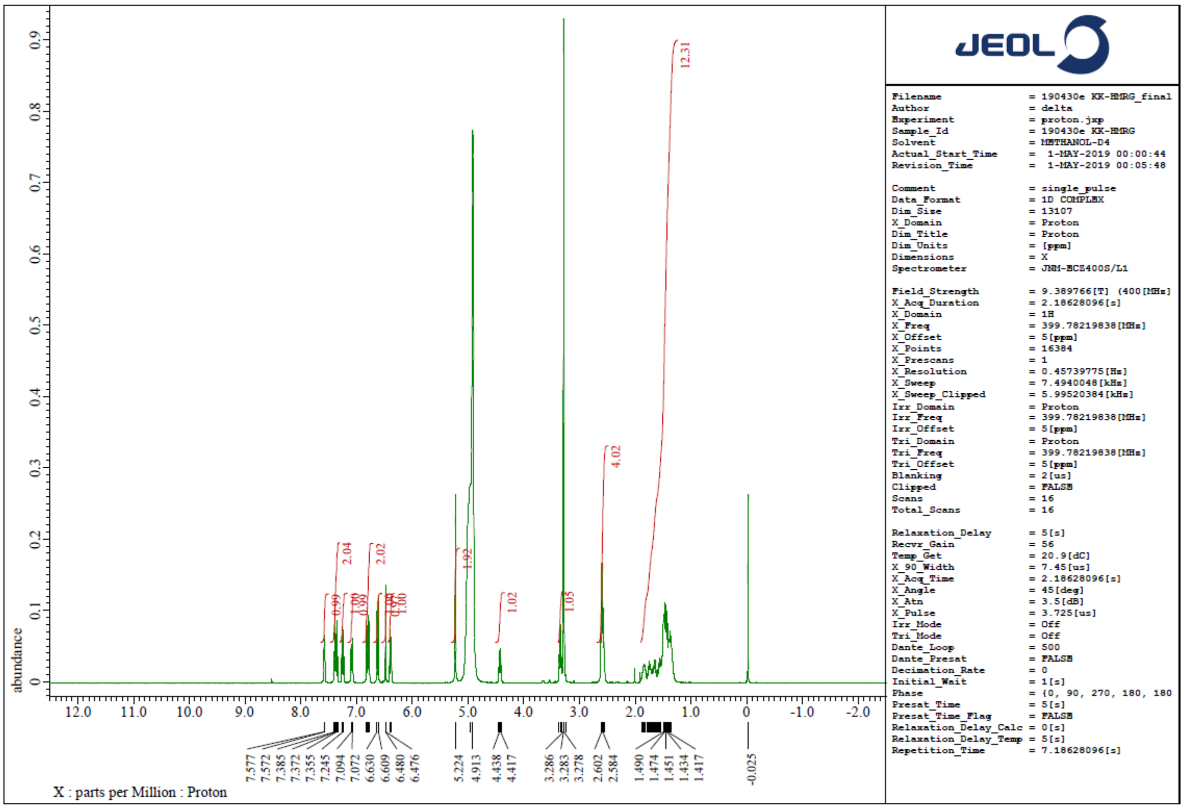
JEOL
Filename = 190430e KK-HMRG_final
Author = delta
Experiment = proton.jxp
Sample_Id = 190430e KK-HMRG
Solvent = METHANOL-D4
Actual_Start_Time = 1-MAY-2019 00:00:44
Revision_Time = 1-MAY-2019 00:05:48
Comment = single_pulse
Data_Format = 1D COMPLEX
Dim_Size = 13107
X_Domain = Proton
Dim_Title = Proton
Dim_Units = [ppm]
Dimensions = X
Spectrometer = JNM-ECZ400S/L1
Field_Strength = 9.389766[T] (400[MHz]
X_Acq_Duration = 2.18628096[s]
X_Domain = 1H
X_Freq = 399.78219838[MHz]
X_Offset = 5[ppm]
X_Points = 16384
X_Prescans = 1
X_Resolution = 0.45739775[Hz]
X_Sweep = 7.4940048[kHz]
X_Sweep_Clipped = 5.99520384[kHz]
Irr_Domain = Proton
Irr_Freq = 399.78219838[MHz]
Irr_Offset = 5[ppm]
Tri_Domain = Proton
Tri_Freq = 399.78219838[MHz]
Tri_Offset = 5[ppm]
Blanking = 2[us]
Clipped = FALSE
Scans = 16
Total_Scans = 16
Relaxation_Delay = 5[s]
Recvr_Gain = 56
Temp_Get = 20.9[dC]
X_90_Width = 7.45[us]
X_Acq_Time = 2.18628096[s]
X_Angle = 45[deg]
X_Atn = 3.5[dB]
X_Pulse = 3.725[us]
Irr_Mode = Off
Tri_Mode = Off
Dante_Loop = 500
Dante_Presat = FALSE
Decimation_Rate = 0
Initial_Wait = 1[s]
Phase = {0, 90, 270, 180, 180
Presat_Time = 5[s]
Presat_Time_Flag = FALSE
Relaxation_Delay_Calc = 0[s]
Relaxation_Delay_Temp = 5[s]
Repetition_Time = 7.18628096[s]
abundance
X : parts per Million : Proton
12.31
4.02
2.04
2.02
1.92
1.02
1.05
7.577
7.572
7.385
7.372
7.355
7.245
7.094
7.072
6.630
6.609
6.480
6.476
5.224
4.913
4.438
4.417
3.286
3.283
3.278
2.602
2.584
1.490
1.474
1.451
1.434
1.417
-0.025

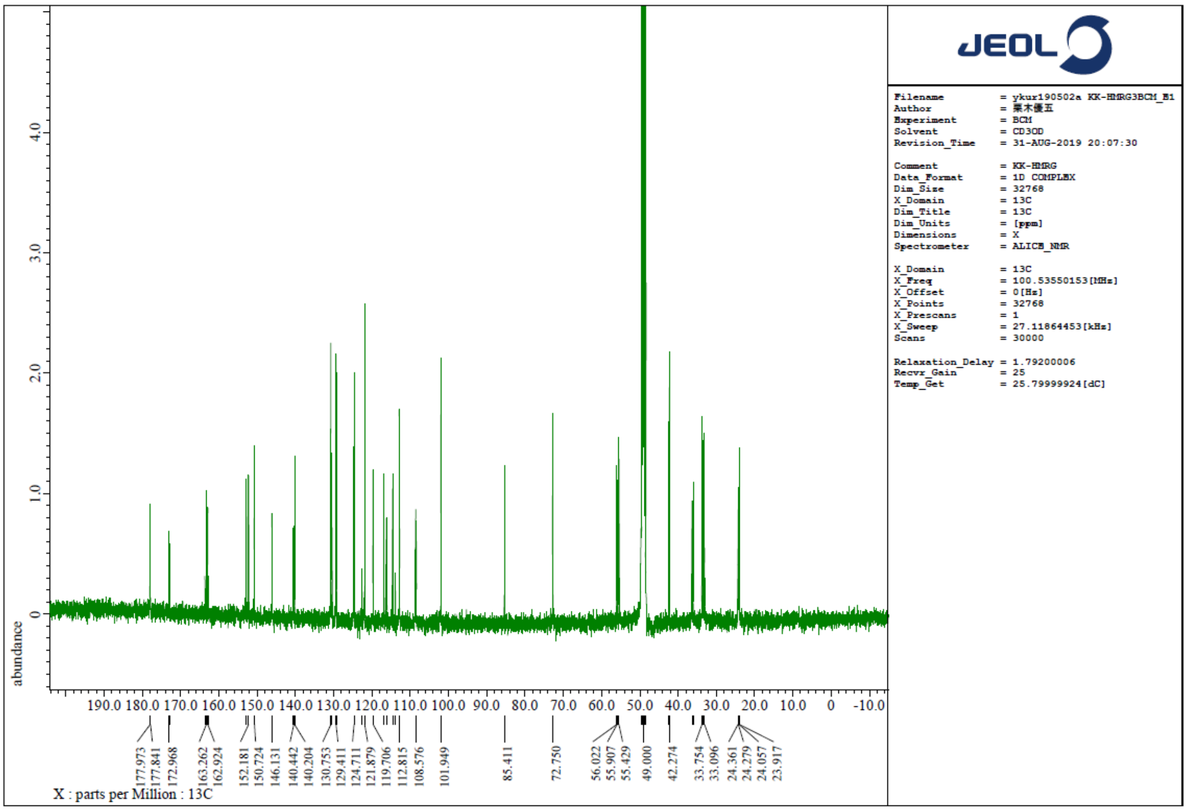
JEOL
Filename = ykur190502a KK-HMRG3BCM_B1
Author = 栗木優五
Experiment = BCM
Solvent = CD3OD
Revision_Time = 31-AUG-2019 20:07:30
Comment = KK-HMRG
Data_Format = 1D COMPLEX
Dim_Size = 32768
X_Domain = 13C
Dim_Title = 13C
Dim_Units = [ppm]
Dimensions = X
Spectrometer = ALICE_NMR
X_Domain = 13C
X_Freq = 100.53550153[MHz]
X_Offset = 0[Hz]
X_Points = 32768
X_Prescans = 1
X_Sweep = 27.11864453[kHz]
Scans = 30000
Relaxation_Delay = 1.79200006
Recvr_Gain = 25
Temp_Get = 25.79999924[dC]
abundance
X : parts per Million : 13C
177.973
177.841
172.968
163.262
162.924
152.181
150.724
146.131
140.442
140.204
130.753
129.411
124.711
121.879
119.706
112.815
108.576
101.949
85.411
72.750
56.022
55.907
55.429
49.000
42.274
33.754
33.096
24.361
24.279
24.057
23.917

Data:ykur190831a
Comment:KK-HMRG
Description:
Ionization Mode:Corona ESI+
History:Centroid[Peak Detect[Centroid,30,Area];Smooth[5]];Average(MS[1] 0.32..0.45)

Acquired:2019/08/31 22:38:31
Operator:AccuTOF
m/z Calibration File:ESI+_1000_180622
Created:2019/08/31 22:54:43
Created by:AccuTOF

Charge number:1 Tolerance:5.00[mDa] Unsaturation Number:−1.5 .. 500.0 (Fraction:Both)
Element:^{12}C:32 .. 32, ^{1}H:41 .. 41, ^{14}N:6 .. 6, ^{16}O:4 .. 4, ^{28}Si:0 .. 0

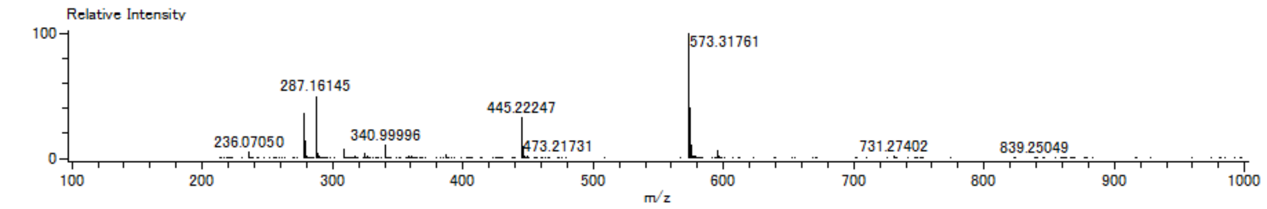

Mass	Intensity	Calc. Mass	Mass Difference [mDa]	Mass Difference [ppm]	Possible Formula	Unsaturation Number
573.31761	213292.33	573.31893	−1.32	−2.30	$^{12}C_{32}{}^{1}H_{41}{}^{14}N_{6}{}^{16}O_{4}$	15.5

Figure S5. ^{1}H, ^{13}C NMR and HRMS spectra of KK-HMRG obtained by liquid-phase synthesis.

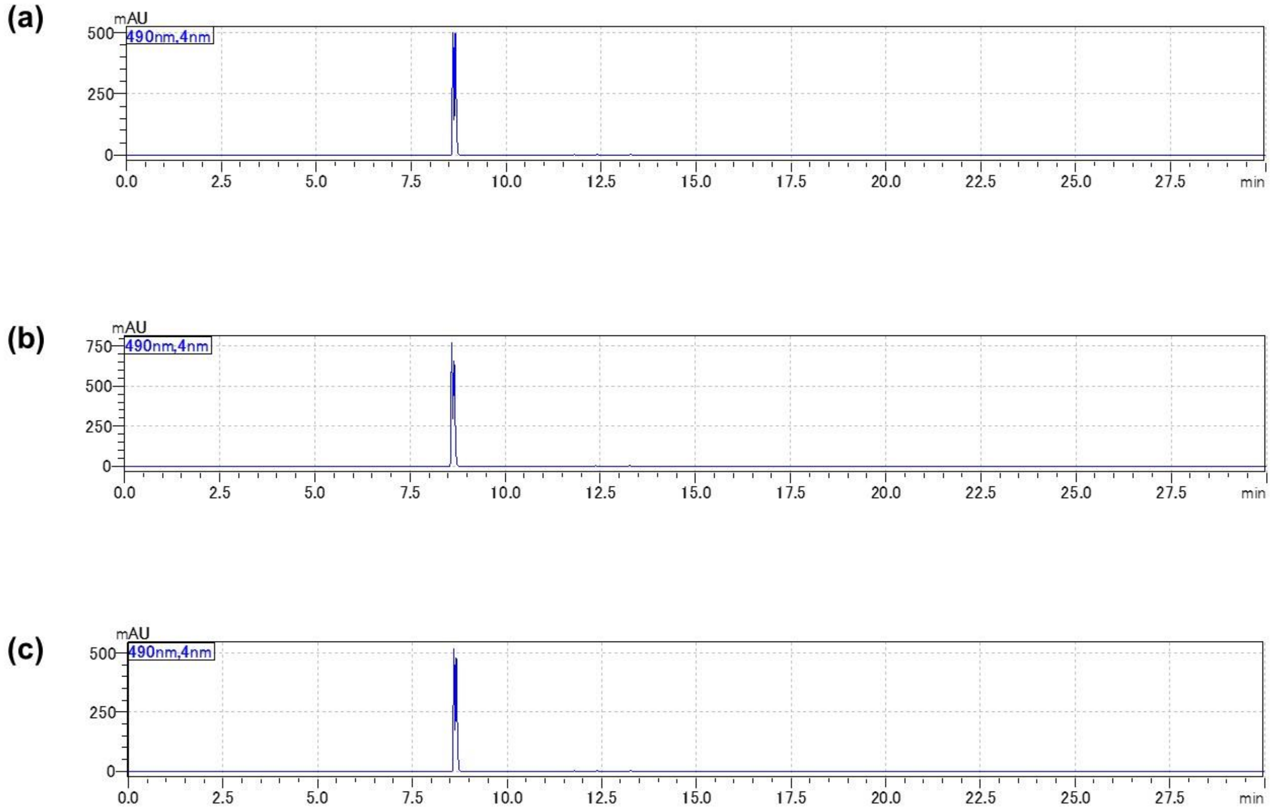

Figure S6. LC-MS analyses of KK-HMRG obtained by liquid-phase or solid-phase synthesis.

(a) KK-HMRG (solid phase), (b) KK-HMRG (liquid phase) and (c) mixture of KK-HMRGs (solid phase and liquid phase) were analyzed by LC-MS. The LC-MS (eluent; 0.1 % formic acid in H_2O/MeCN = 99/1 for 5 min, then to 20/80 in 15 min in linear gradient) chromatograms are shown. Absorbance at 490 nm was detected. KK-HMRG synthesized in the solid phase was purified by HPLC before these analyses.

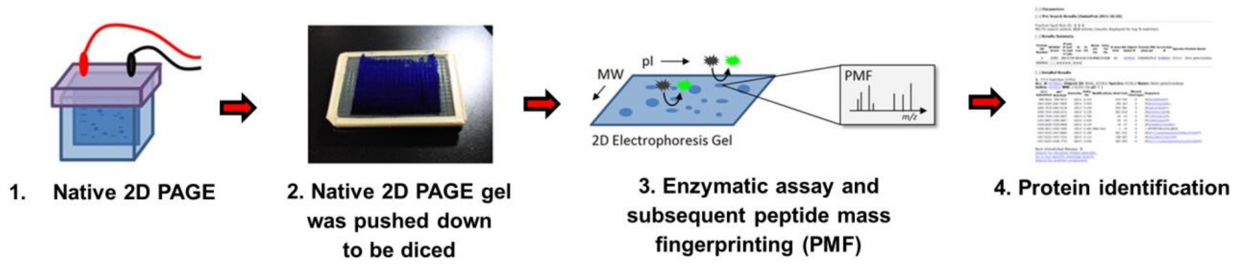

Figure S7. Flowchart of diced electrophoresis gel (DEG) assay[8].

In DEG assay, the proteome in the lysate is separated by means of two-dimensional polyacrylamide gel electrophoresis (PAGE) under nondenaturing conditions; next, the gels are diced and separately loaded into wells of multiwell plates with a specially developed cutter-plate system, and the activity assay is performed in them.

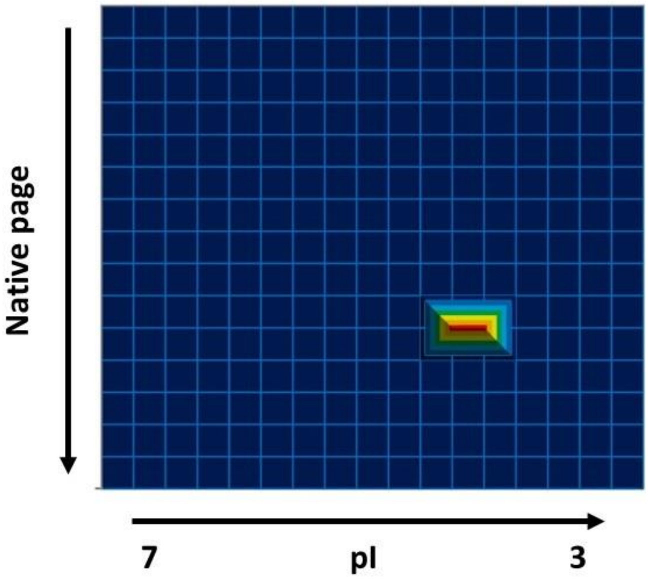

Figure S8. Result of two-dimensional diced electrophoresis gel (DEG) assay of 26 μg lung squamous cell carcinoma lysate with 1 μM KK-HMRG in phosphate-buffered saline after isoelectric focusing (pH 3-7) and native PAGE. The fluorescence increase rate after 16.5 h incubation is plotted.

List of proteins included in the fluorescent spot of the DEG assay (Figure 4a).

1	Keratin, type II cytoskeletal 1 OS=Homo sapiens OX=9606 GN=KRT1 PE=1 SV=6
2	Cluster of Keratin, type II cytoskeletal 2 epidermal OS=Homo sapiens OX=9606 GN=KRT2 PE=1 SV=2 (K22E_HUMAN)
2.1	Keratin, type II cytoskeletal 2 epidermal OS=Homo sapiens OX=9606 GN=KRT2 PE=1 SV=2
2.2	Keratin, type II cytoskeletal 5 OS=Homo sapiens OX=9606 GN=KRT5 PE=1 SV=3
2.3	Keratin, type II cytoskeletal 6C OS=Homo sapiens OX=9606 GN=KRT6C PE=1 SV=3
3	Keratin, type I cytoskeletal 9 OS=Homo sapiens OX=9606 GN=KRT9 PE=1 SV=3
4	Keratin, type I cytoskeletal 10 OS=Homo sapiens OX=9606 GN=KRT10 PE=1 SV=6
5	Serum albumin OS=Homo sapiens OX=9606 GN=ALB PE=1 SV=2
6	Plastin-2 OS=Homo sapiens OX=9606 GN=LCP1 PE=1 SV=6
7	Cluster of Keratin, type I cytoskeletal 14 OS=Homo sapiens OX=9606 GN=KRT14 PE=1 SV=4 (K1C14_HUMAN)
7.1	Keratin, type I cytoskeletal 14 OS=Homo sapiens OX=9606 GN=KRT14 PE=1 SV=4
7.2	Keratin, type I cytoskeletal 17 OS=Homo sapiens OX=9606 GN=KRT17 PE=1 SV=2
7.3	Keratin, type I cytoskeletal 16 OS=Homo sapiens OX=9606 GN=KRT16 PE=1 SV=4
8	Desmoplakin OS=Homo sapiens OX=9606 GN=DSP PE=1 SV=3
9	Cluster of Keratin, type II cuticular Hb6 OS=Homo sapiens OX=9606 GN=KRT86 PE=1 SV=1 (KRT86_HUMAN)
9.1	Keratin, type II cuticular Hb6 OS=Homo sapiens OX=9606 GN=KRT86 PE=1 SV=1
9.2	Keratin, type II cuticular Hb5 OS=Homo sapiens OX=9606 GN=KRT85 PE=1 SV=1
10	Neutral alpha-glucosidase AB OS=Homo sapiens OX=9606 GN=GANAB PE=1 SV=3
11	Ubiquitin-like modifier-activating enzyme 1 OS=Homo sapiens OX=9606 GN=UBA1 PE=1 SV=3
12	Cluster of Heat shock protein HSP 90-beta OS=Homo sapiens OX=9606 GN=HSP90AB1 PE=1 SV=4 (HS90B_HUMAN)
12.1	Heat shock protein HSP 90-beta OS=Homo sapiens OX=9606 GN=HSP90AB1 PE=1 SV=4
12.2	Heat shock protein HSP 90-alpha OS=Homo sapiens OX=9606 GN=HSP90AA1 PE=1 SV=5
13	Lysosomal alpha-glucosidase OS=Homo sapiens OX=9606 GN=GAA PE=1 SV=4
14	Endoplasmic reticulum chaperone BiP OS=Homo sapiens OX=9606 GN=HSPA5 PE=1 SV=2
15	Ras GTPase-activating-like protein IQGAP1 OS=Homo sapiens OX=9606 GN=IQGAP1 PE=1 SV=1
16	Protein disulfide-isomerase A4 OS=Homo sapiens OX=9606 GN=PDIA4 PE=1 SV=2
17	Desmoglein-1 OS=Homo sapiens OX=9606 GN=DSG1 PE=1 SV=2
18	Keratin, type II cytoskeletal 1b OS=Homo sapiens OX=9606 GN=KRT77 PE=1 SV=3
19	Nicotinate phosphoribosyltransferase OS=Homo sapiens OX=9606 GN=NAPRT PE=1 SV=2
20	Keratin, type II cytoskeletal 78 OS=Homo sapiens OX=9606 GN=KRT78 PE=1 SV=2
21	Junction plakoglobin OS=Homo sapiens OX=9606 GN=JUP PE=1 SV=3
22	Glutathione S-transferase P OS=Homo sapiens OX=9606 GN=GSTP1 PE=1 SV=2
23	Nucleolin OS=Homo sapiens OX=9606 GN=NCL PE=1 SV=3

24	Puromycin-sensitive aminopeptidase OS=Homo sapiens OX=9606 GN=NPEPPS PE=1 SV=2
25	Cluster of Actin, cytoplasmic 1 OS=Homo sapiens OX=9606 GN=ACTB PE=1 SV=1 (ACTB_HUMAN)
25.1	Actin, cytoplasmic 1 OS=Homo sapiens OX=9606 GN=ACTB PE=1 SV=1
25.2	Actin, aortic smooth muscle OS=Homo sapiens OX=9606 GN=ACTA2 PE=1 SV=1
26	Hornerin OS=Homo sapiens OX=9606 GN=HRNR PE=1 SV=2
27	Heat shock cognate 71 kDa protein OS=Homo sapiens OX=9606 GN=HSPA8 PE=1 SV=1
28	Ribonuclease inhibitor OS=Homo sapiens OX=9606 GN=RNH1 PE=1 SV=2
29	Beta-galactosidase OS=Homo sapiens OX=9606 GN=GLB1 PE=1 SV=2
30	Heterogeneous nuclear ribonucleoprotein U-like protein 1 OS=Homo sapiens OX=9606 GN=HNRNPUL1 PE=1 SV=2
31	Isocitrate dehydrogenase [NADP] cytoplasmic OS=Homo sapiens OX=9606 GN=IDH1 PE=1 SV=2
32	Filaggrin-2 OS=Homo sapiens OX=9606 GN=FLG2 PE=1 SV=1
33	Hemopexin OS=Homo sapiens OX=9606 GN=HPX PE=1 SV=2
34	Cluster of Keratin, type I cuticular Ha3-II OS=Homo sapiens OX=9606 GN=KRT33B PE=1 SV=3 (KT33B_HUMAN)
34.1	Keratin, type I cuticular Ha3-II OS=Homo sapiens OX=9606 GN=KRT33B PE=1 SV=3
34.2	Keratin, type I cuticular Ha1 OS=Homo sapiens OX=9606 GN=KRT31 PE=1 SV=3
35	Alpha-1-antitrypsin OS=Homo sapiens OX=9606 GN=SERPINA1 PE=1 SV=3
36	Annexin A2 OS=Homo sapiens OX=9606 GN=ANXA2 PE=1 SV=2
37	Carbonyl reductase [NADPH] 1 OS=Homo sapiens OX=9606 GN=CBR1 PE=1 SV=3
38	40S ribosomal protein SA OS=Homo sapiens OX=9606 GN=RPSA PE=1 SV=4
39	Thymidine phosphorylase OS=Homo sapiens OX=9606 GN=TYMP PE=1 SV=2
40	Arginase-1 OS=Homo sapiens OX=9606 GN=ARG1 PE=1 SV=2
41	Lupus La protein OS=Homo sapiens OX=9606 GN=SSB PE=1 SV=2
42	Serpin B12 OS=Homo sapiens OX=9606 GN=SERPINB12 PE=1 SV=1
43	Cluster of Heterogeneous nuclear ribonucleoprotein H OS=Homo sapiens OX=9606 GN=HNRNPH1 PE=1 SV=4 (HNRH1_HUMAN)
43.1	Heterogeneous nuclear ribonucleoprotein H OS=Homo sapiens OX=9606 GN=HNRNPH1 PE=1 SV=4
43.2	Heterogeneous nuclear ribonucleoprotein H2 OS=Homo sapiens OX=9606 GN=HNRNPH2 PE=1 SV=1
44	Glyceraldehyde-3-phosphate dehydrogenase OS=Homo sapiens OX=9606 GN=GAPDH PE=1 SV=3
45	Keratinocyte proline-rich protein OS=Homo sapiens OX=9606 GN=KPRP PE=1 SV=1
46	Protein disulfide-isomerase OS=Homo sapiens OX=9606 GN=P4HB PE=1 SV=3
47	Protein-glutamine gamma-glutamyltransferase E OS=Homo sapiens OX=9606 GN=TGM3 PE=1 SV=4
48	Antithrombin-III OS=Homo sapiens OX=9606 GN=SERPINC1 PE=1 SV=1
49	Calreticulin OS=Homo sapiens OX=9606 GN=CALR PE=1 SV=1
50	Chloride intracellular channel protein 1 OS=Homo sapiens OX=9606 GN=CLIC1 PE=1 SV=4
51	Desmocollin-1 OS=Homo sapiens OX=9606 GN=DSC1 PE=1 SV=2
52	Glucosidase 2 subunit beta OS=Homo sapiens OX=9606 GN=PRKCSH PE=1 SV=2

53	Importin subunit beta-1 OS=Homo sapiens OX=9606 GN=KPNB1 PE=1 SV=2
54	Keratin, type II cytoskeletal 80 OS=Homo sapiens OX=9606 GN=KRT80 PE=1 SV=2
55	Keratin, type I cuticular Ha4 OS=Homo sapiens OX=9606 GN=KRT34 PE=1 SV=2
56	Leukotriene A-4 hydrolase OS=Homo sapiens OX=9606 GN=LTA4H PE=1 SV=2
57	Heterogeneous nuclear ribonucleoprotein U-like protein 2 OS=Homo sapiens OX=9606 GN=HNRNPUL2 PE=1 SV=1
58	14-3-3 protein zeta/delta OS=Homo sapiens OX=9606 GN=YWHAZ PE=1 SV=1
59	Heterogeneous nuclear ribonucleoprotein U OS=Homo sapiens OX=9606 GN=HNRNPU PE=1 SV=6
60	Cathepsin D OS=Homo sapiens OX=9606 GN=CTSD PE=1 SV=1
61	Peroxiredoxin-6 OS=Homo sapiens OX=9606 GN=PRDX6 PE=1 SV=3
62	Transaldolase OS=Homo sapiens OX=9606 GN=TALDO1 PE=1 SV=2
63	Vitamin D-binding protein OS=Homo sapiens OX=9606 GN=GC PE=1 SV=1
64	Gasdermin-A OS=Homo sapiens OX=9606 GN=GSDMA PE=1 SV=4
65	Calpain-2 catalytic subunit OS=Homo sapiens OX=9606 GN=CAPN2 PE=1 SV=6
66	Transcriptional activator protein Pur-alpha OS=Homo sapiens OX=9606 GN=PURA PE=1 SV=2
67	60S acidic ribosomal protein P0 OS=Homo sapiens OX=9606 GN=RPLP0 PE=1 SV=1
68	Cytoplasmic aconitate hydratase OS=Homo sapiens OX=9606 GN=ACO1 PE=1 SV=3
69	Dermcidin OS=Homo sapiens OX=9606 GN=DCD PE=1 SV=2
70	Endoplasmin OS=Homo sapiens OX=9606 GN=HSP90B1 PE=1 SV=1
71	Hsc70-interacting protein OS=Homo sapiens OX=9606 GN=ST13 PE=1 SV=2
72	Keratin, type II cuticular Hb4 OS=Homo sapiens OX=9606 GN=KRT84 PE=2 SV=2
73	Heterogeneous nuclear ribonucleoprotein K OS=Homo sapiens OX=9606 GN=HNRNPK PE=1 SV=1
74	Hypoxia up-regulated protein 1 OS=Homo sapiens OX=9606 GN=HYOU1 PE=1 SV=1
75	Caspase-14 OS=Homo sapiens OX=9606 GN=CASP14 PE=1 SV=2
76	Transforming protein RhoA OS=Homo sapiens OX=9606 GN=RHOA PE=1 SV=1
77	Catalase OS=Homo sapiens OX=9606 GN=CAT PE=1 SV=3
78	L-lactate dehydrogenase B chain OS=Homo sapiens OX=9606 GN=LDHB PE=1 SV=2
79	Short/branched chain specific acyl-CoA dehydrogenase, mitochondrial OS=Homo sapiens OX=9606 GN=ACADSB PE=1 SV=1
80	Serine/threonine-protein phosphatase PP1-beta catalytic subunit OS=Homo sapiens OX=9606 GN=PPP1CB PE=1 SV=3
81	DNA damage-binding protein 1 OS=Homo sapiens OX=9606 GN=DDB1 PE=1 SV=1
82	Protein-glutamine gamma-glutamyltransferase K OS=Homo sapiens OX=9606 GN=TGM1 PE=1 SV=4
83	Plakophilin-1 OS=Homo sapiens OX=9606 GN=PKP1 PE=1 SV=2
84	Lumican OS=Homo sapiens OX=9606 GN=LUM PE=1 SV=2
85	Peroxiredoxin-2 OS=Homo sapiens OX=9606 GN=PRDX2 PE=1 SV=5
86	Heat shock 70 kDa protein 4 OS=Homo sapiens OX=9606 GN=HSPA4 PE=1 SV=4
87	Skin-specific protein 32 OS=Homo sapiens OX=9606 GN=XP32 PE=1 SV=1

88	Peroxiredoxin-1 OS=Homo sapiens OX=9606 GN=PRDX1 PE=1 SV=1
89	Filaggrin OS=Homo sapiens OX=9606 GN=FLG PE=1 SV=3
90	Inter-alpha-trypsin inhibitor heavy chain H4 OS=Homo sapiens OX=9606 GN=ITIH4 PE=1 SV=4
91	Hemoglobin subunit alpha OS=Homo sapiens OX=9606 GN=HBA1 PE=1 SV=2
92	Plasma protease C1 inhibitor OS=Homo sapiens OX=9606 GN=SERPING1 PE=1 SV=2
93	14-3-3 protein theta OS=Homo sapiens OX=9606 GN=YWHAQ PE=1 SV=1
94	Heterogeneous nuclear ribonucleoprotein Q OS=Homo sapiens OX=9606 GN=SYNCRIP PE=1 SV=2
95	Dipeptidyl peptidase 3 OS=Homo sapiens OX=9606 GN=DPP3 PE=1 SV=2
96	DIS3-like exonuclease 2 OS=Homo sapiens OX=9606 GN=DIS3L2 PE=1 SV=4
97	Prolactin-inducible protein OS=Homo sapiens OX=9606 GN=PIP PE=1 SV=1
98	Superoxide dismutase [Cu-Zn] OS=Homo sapiens OX=9606 GN=SOD1 PE=1 SV=2
99	Transcriptional activator protein Pur-beta OS=Homo sapiens OX=9606 GN=PURB PE=1 SV=3
100	14-3-3 protein epsilon OS=Homo sapiens OX=9606 GN=YWHAE PE=1 SV=1
101	Gamma-glutamylcyclotransferase OS=Homo sapiens OX=9606 GN=GGCT PE=1 SV=1
102	Interleukin enhancer-binding factor 2 OS=Homo sapiens OX=9606 GN=ILF2 PE=1 SV=2
103	Replication protein A 70 kDa DNA-binding subunit OS=Homo sapiens OX=9606 GN=RPA1 PE=1 SV=2
104	60S ribosomal protein L12 OS=Homo sapiens OX=9606 GN=RPL12 PE=1 SV=1
105	60 kDa SS-A/Ro ribonucleoprotein OS=Homo sapiens OX=9606 GN=TROVE2 PE=1 SV=2
106	Suprabasin OS=Homo sapiens OX=9606 GN=SBSN PE=1 SV=2
107	Protein SETSIP OS=Homo sapiens OX=9606 GN=SETSIP PE=1 SV=1
108	60S ribosomal protein L5 OS=Homo sapiens OX=9606 GN=RPL5 PE=1 SV=3
109	Keratin-associated protein 2-1 OS=Homo sapiens OX=9606 GN=KRTAP2-1 PE=2 SV=2
110	Zinc-alpha-2-glycoprotein OS=Homo sapiens OX=9606 GN=AZGP1 PE=1 SV=2
111	Protein phosphatase 1 regulatory subunit 7 OS=Homo sapiens OX=9606 GN=PPP1R7 PE=1 SV=1
112	Corneodesmosin OS=Homo sapiens OX=9606 GN=CDSN PE=1 SV=3
113	Hemoglobin subunit beta OS=Homo sapiens OX=9606 GN=HBB PE=1 SV=2
114	Transmembrane glycoprotein NMB OS=Homo sapiens OX=9606 GN=GPNMB PE=1 SV=2
115	Serpin A12 OS=Homo sapiens OX=9606 GN=SERPINA12 PE=1 SV=1
116	Fibulin-1 OS=Homo sapiens OX=9606 GN=FBLN1 PE=1 SV=4
117	Extracellular matrix protein 1 OS=Homo sapiens OX=9606 GN=ECM1 PE=1 SV=2
118	Heterogeneous nuclear ribonucleoprotein A/B OS=Homo sapiens OX=9606 GN=HNRNPAB PE=1 SV=2
119	Rho guanine nucleotide exchange factor 17 OS=Homo sapiens OX=9606 GN=ARHGEF17 PE=1 SV=1
120	Voltage-dependent calcium channel gamma-6 subunit OS=Homo sapiens OX=9606 GN=CACNG6 PE=2 SV=1
121	Phosphoglycerate kinase 1 OS=Homo sapiens OX=9606 GN=PGK1 PE=1 SV=3
122	Glucocorticoid modulatory element-binding protein 1 OS=Homo sapiens OX=9606 GN=GMEB1 PE=1 SV=2
123	Bleomycin hydrolase OS=Homo sapiens OX=9606 GN=BLMH PE=1 SV=1
124	60 kDa heat shock protein, mitochondrial OS=Homo sapiens OX=9606 GN=HSPD1 PE=1 SV=2
125	Alpha-1B-glycoprotein OS=Homo sapiens OX=9606 GN=A1BG PE=1 SV=4

126	Ubiquitin-60S ribosomal protein L40 OS=Homo sapiens OX=9606 GN=UBA52 PE=1 SV=2
127	Protrudin OS=Homo sapiens OX=9606 GN=ZFYVE27 PE=1 SV=1

Table S3. List of proteins detected by LC-MS/MS analysis of the fluorescent spot in the DEG assay. Enzymes that have aminopeptidase/protease activities are highlighted in green.

[Supplementary note]

Among the highlighted 7 proteins, we excluded cathepsin D, caspase-14 and bleomycin hydrolase because they are often detected in DEG assay as contaminants. Next, judging from the substrate specificity, gamma-glutamylcyclotransferase was also excluded, since it recognizes γ-linked glutamate. Puromycin-sensitive aminopeptidase (PSA), calpain-2, dipeptidyl peptidase 3 (DPP-3) and leucotriene A4 hydrolase were further examined by the use of corresponding inhibitors.

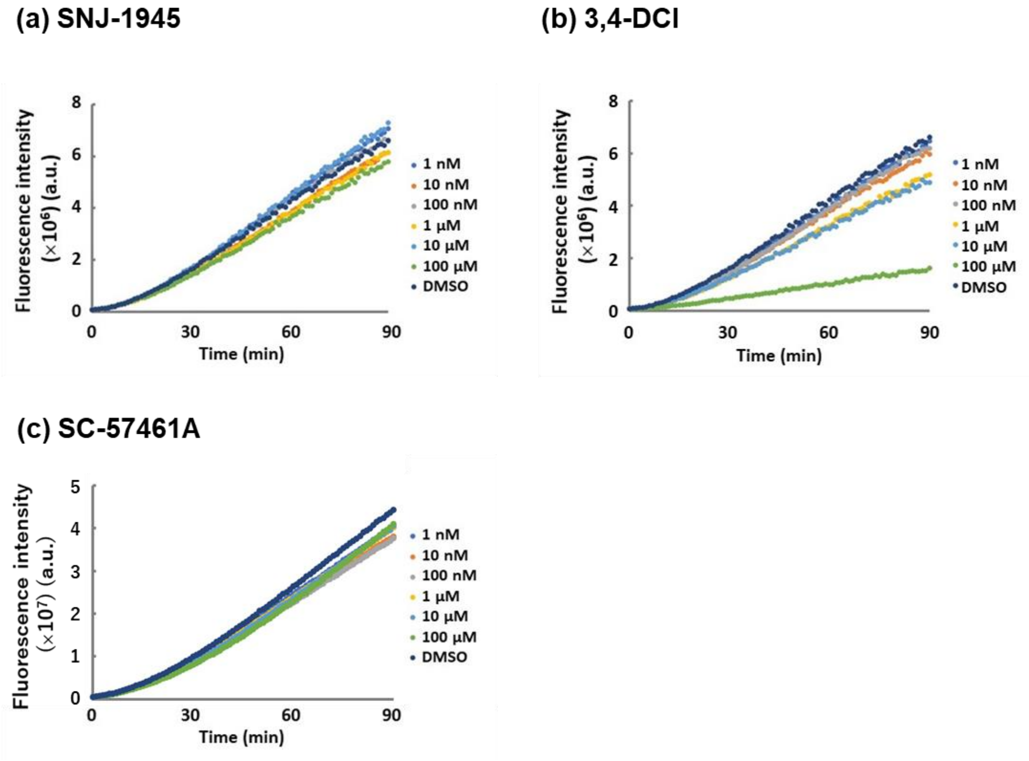

Figure S9. Enzyme assays of KK-HMRG with lung adenocarcinoma lysate in the presence of the inhibitors.

1 μM KK-HMRG was reacted with lung adenocarcinoma lysate in the presence or absence of SNJ-1945 (calpain inhibitor), 3,4-DCI (DPP-3 inhibitor) or SC-57461A (leucotriene A4 hydrolase inhibitor) at various concentrations (n = 4). All assays were carried out at 37 °C in 20 μL total volume of phosphate-buffered saline (pH 7.4) containing 100 mg/L $CaCl_2$ and $MgCl_2{\cdot}6H_2O$ with 0.2 % DMSO as a co-solvent. Excitation/emission wavelengths = 485/535 nm.

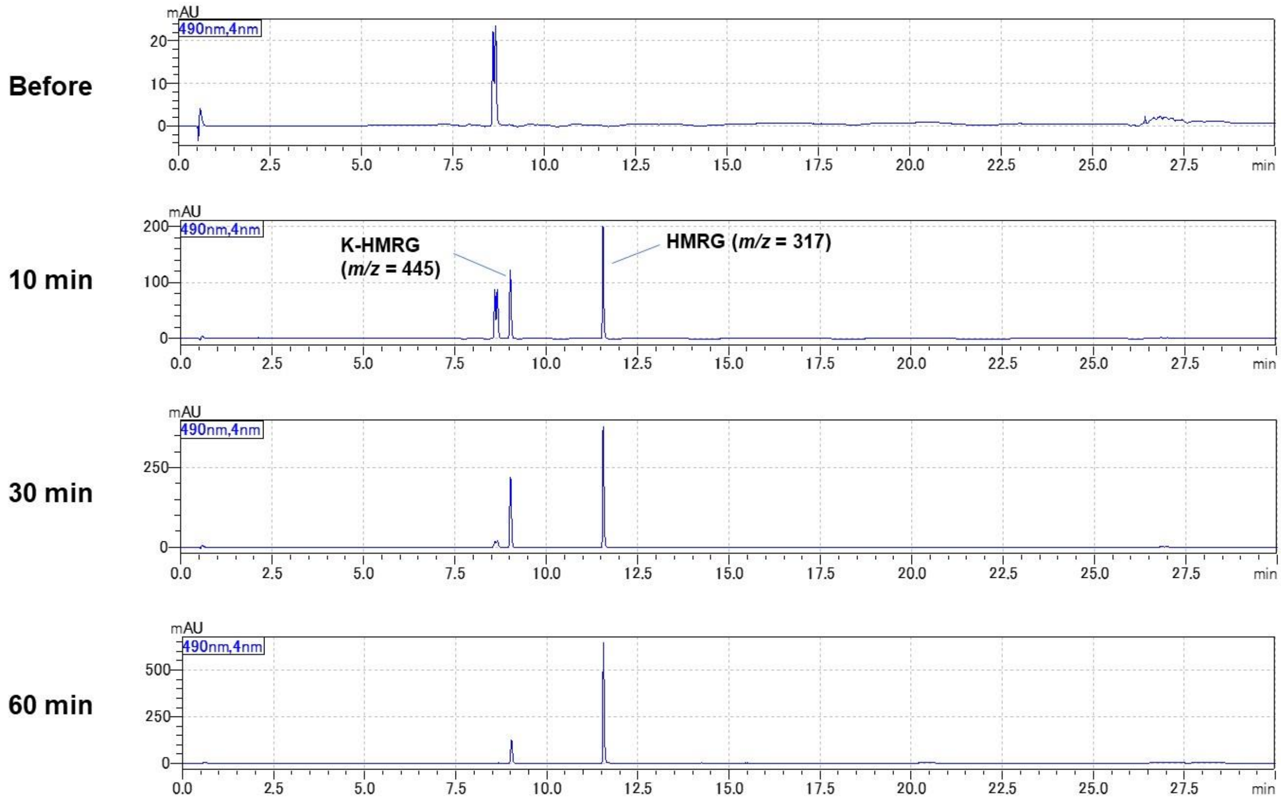

Figure S10. LC-MS analysis of reaction mixtures of KK-HMRG and PSA.

To 1.5 mL of 10 µM probe solution in 10 mM HEPES buffer (pH 7.4) containing 0.1 % DMSO as a co-solvent, 0.22 µg of PSA was added. The reaction mixture was incubated at 37 ºC and 100 µL aliquots were taken at the indicated intervals. An equal volume of 10 % formic acid in MeOH was added to quench the enzymatic reaction, and the mixture was analyzed by LC-MS (eluent; 0.1 % formic acid in H_2O/MeCN = 99/1 for 5 min, then to 20/80 in 15 min in linear gradient). Chromatograms detected at 490 nm are shown.

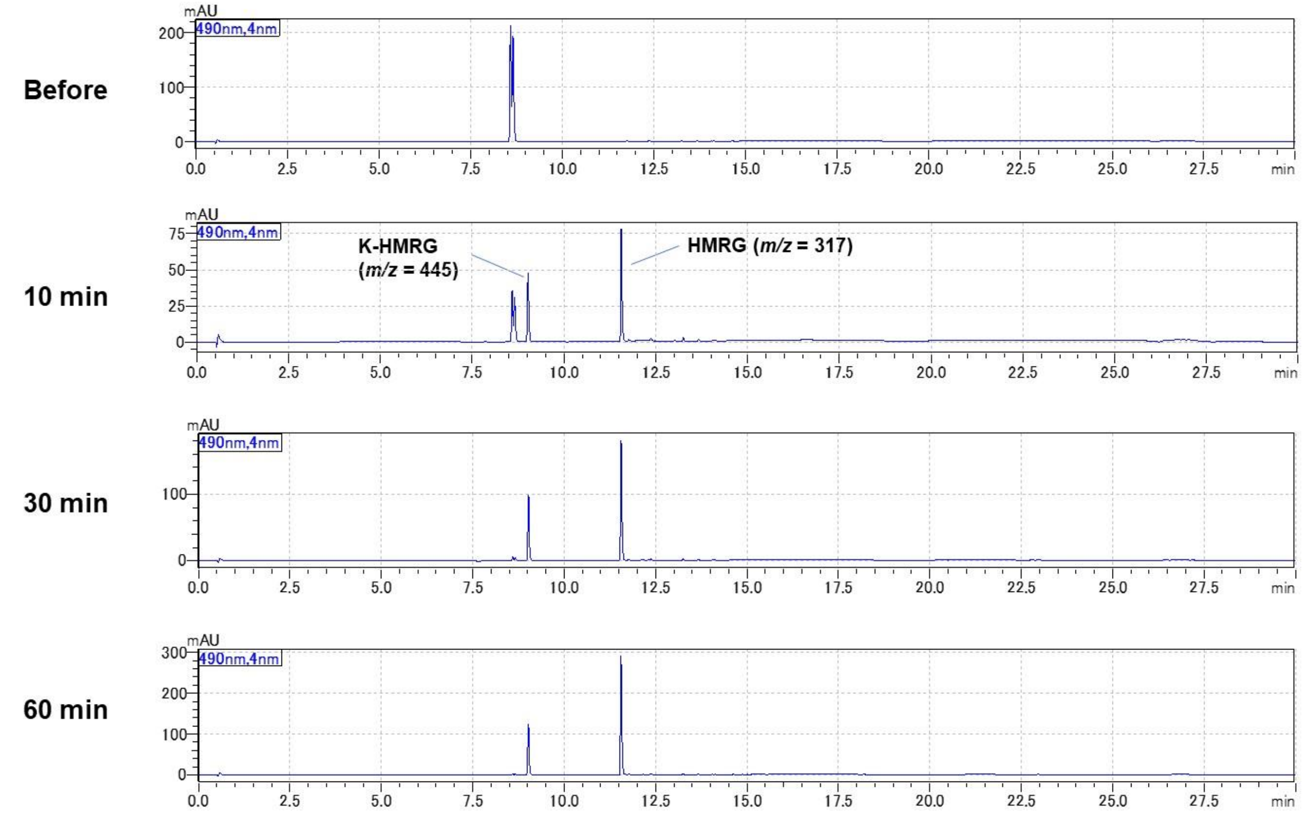

Figure S11. LC-MS analysis of reaction mixtures of KK-HMRG and a lung adenocarcinoma lysate.

To a 500 µL of 10 µM probe solution in 10 mM HEPES buffer (pH 7.4) containing 0.1 % DMSO as a co-solvent, 87.7 µg of lung adenocarcinoma lysate was added. The reaction mixture was incubated at 37 ºC and 100 µL aliquots were taken at the indicated intervals. An equal volume of 10 % formic acid in MeOH was added to quench the enzymatic reaction. The mixture was analyzed by LC-MS (eluent; 0.1 % formic acid in H_2O/MeCN = 99/1 for 5 min, then to 20/80 in 15 min in linear gradient). Chromatograms detected at 490 nm are shown.

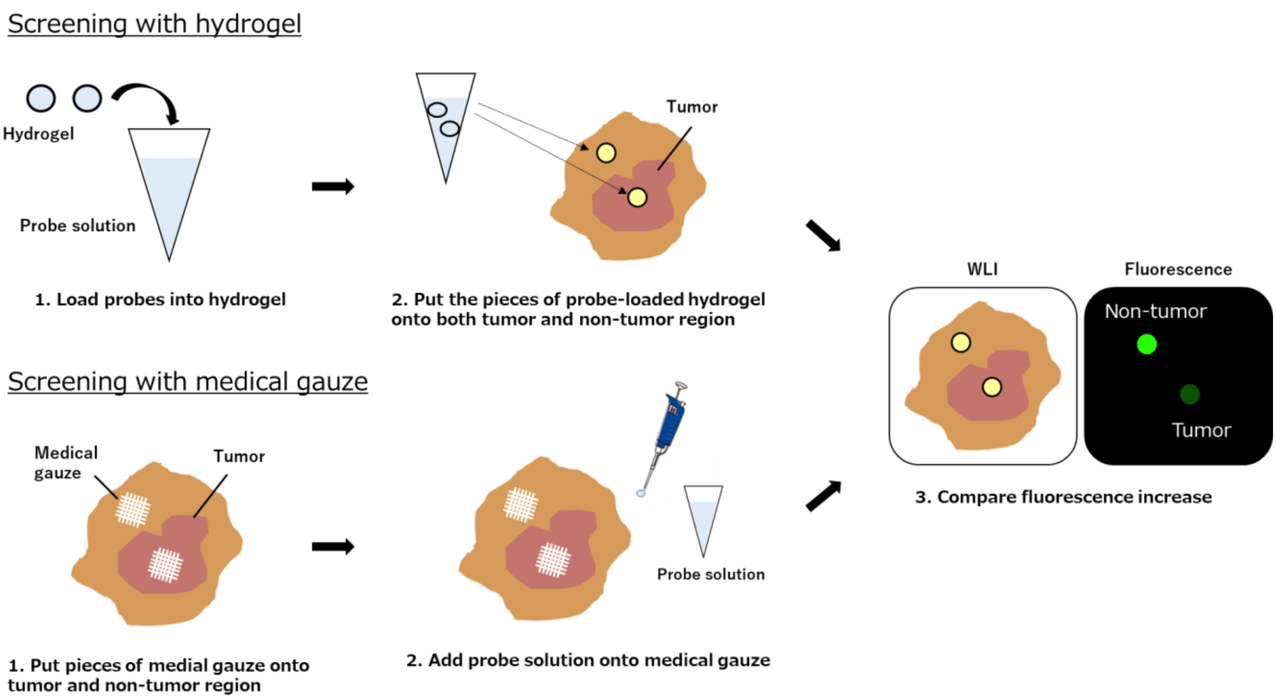

Figure S12. Flowchart of the probe screening on ESD samples with hydrogel or medical gauze.
By utilizing hydrogel or medical gauze as a scaffold to hold the probe solution locally, several probes can be evaluated simultaneously in one ESD sample.

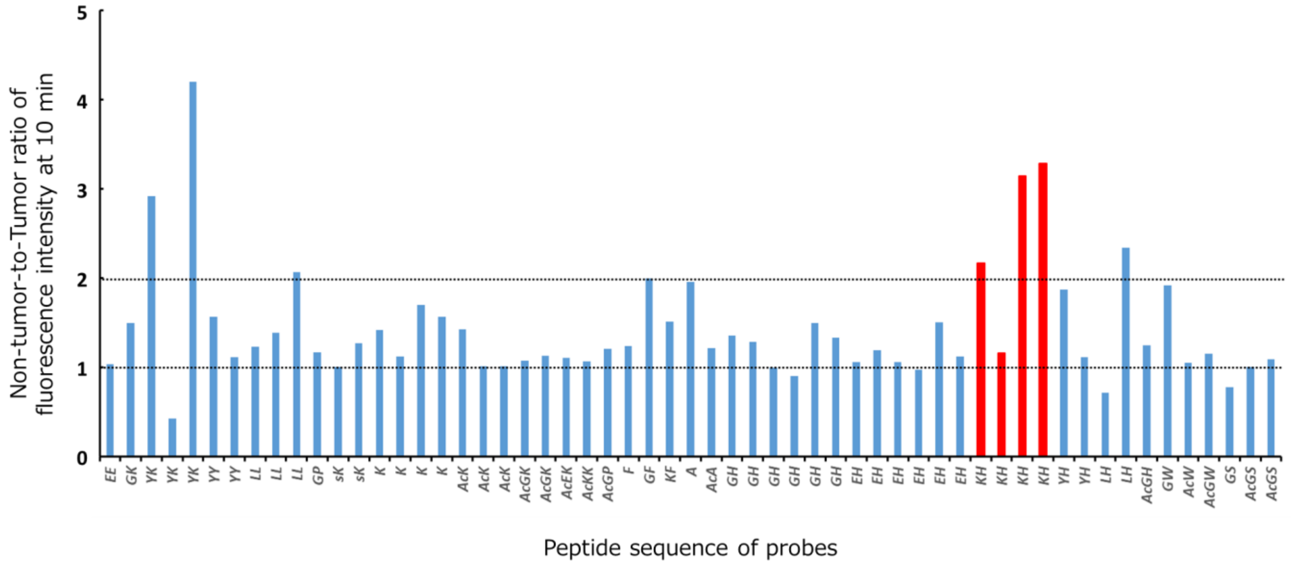

Figure S13. Probe screening on ESD samples of gastric cancer.

The non-tumour-to-tumour ratio of fluorescence intensity after incubation for 10 min was evaluated. Among the tested 29 probes, KH-HMRG showed N/T>2 in 3 samples out of 4 ones (highlighted in red).

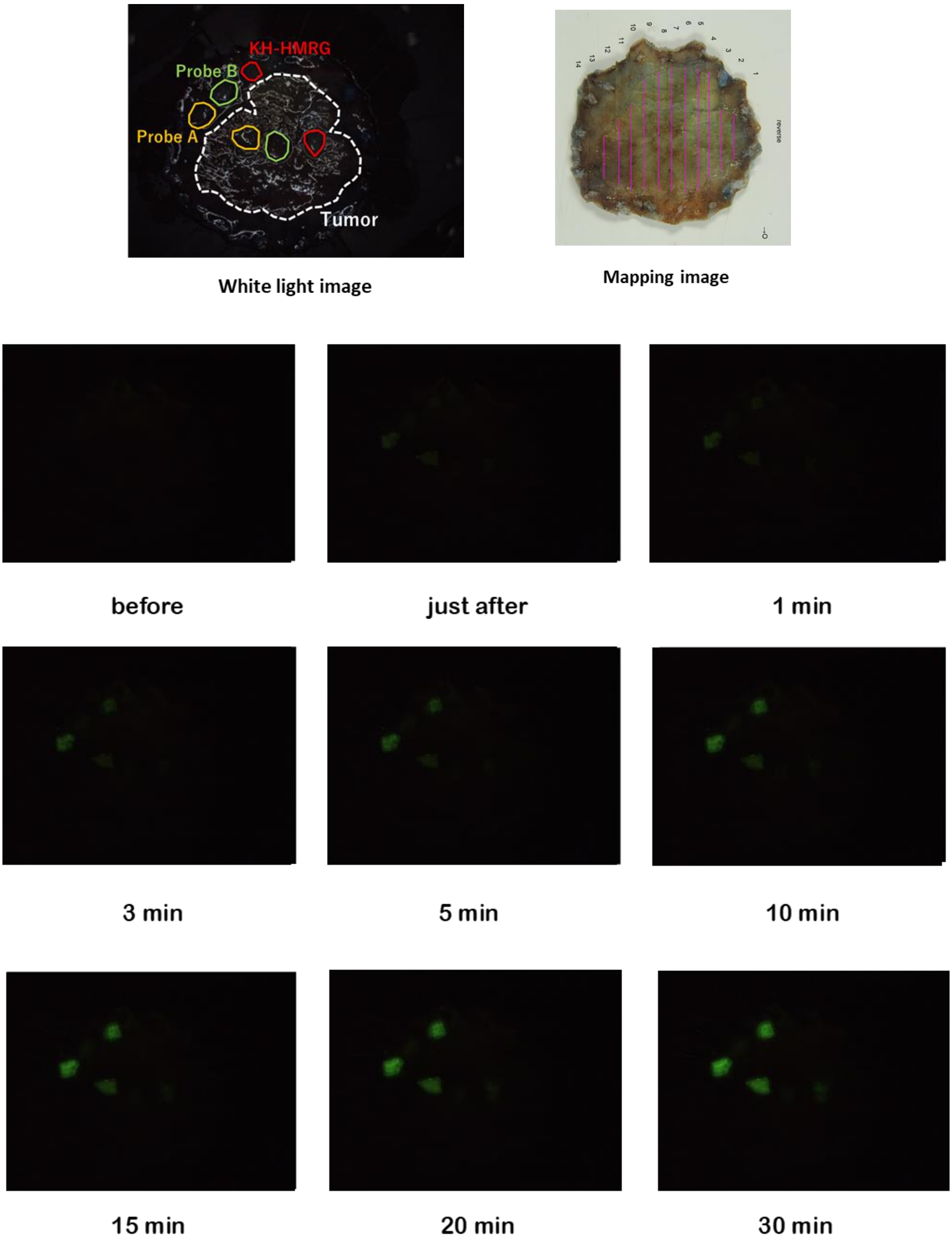

Figure S14. Time-dependent fluorescence increase in a representative case of probe screening with hydrogel. Three probe-loaded gels (LL-, sK- or KH-HMRG) were examined on an ESD sample of gastric cancer (the same sample as in Figure 5a).

Representative successful case #1 (the same sample as in Figure 5b)

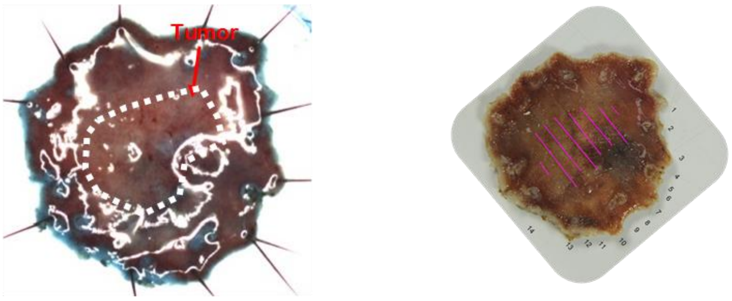

White light   mapping image

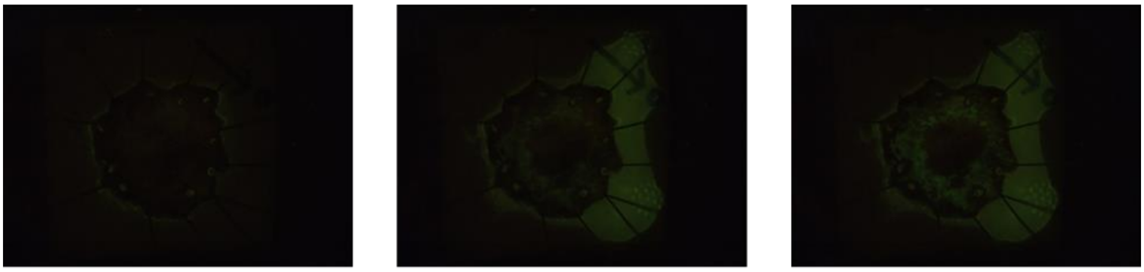

before   just after   1 min

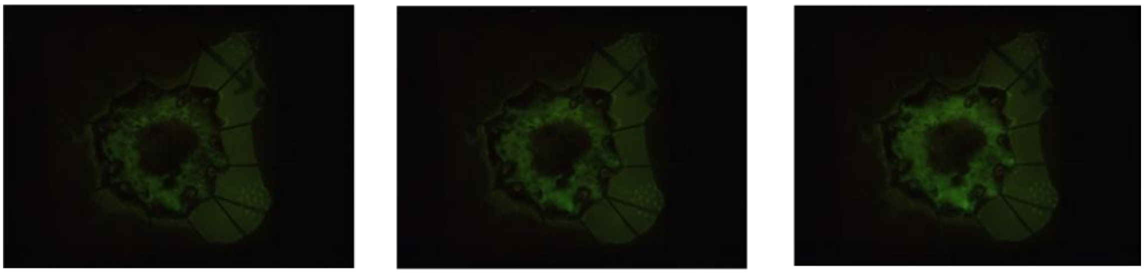

3 min   5 min   10 min

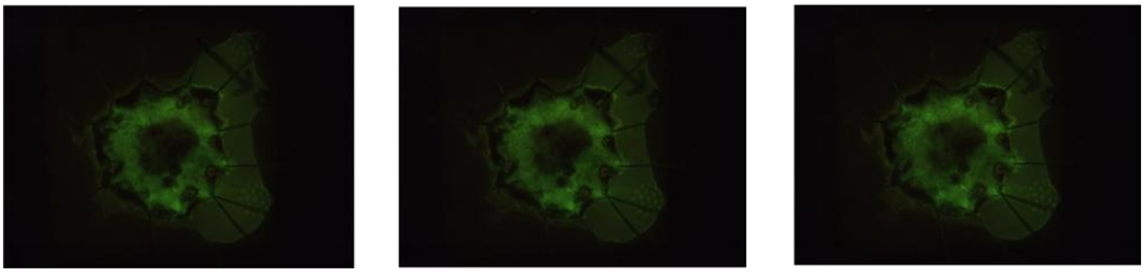

15 min   20 min   30 min

Representative successful case #2

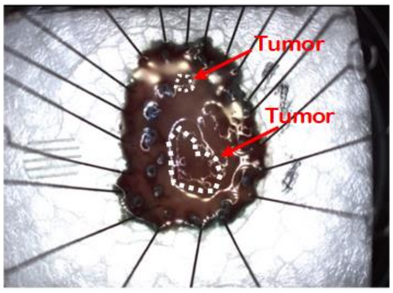

White light

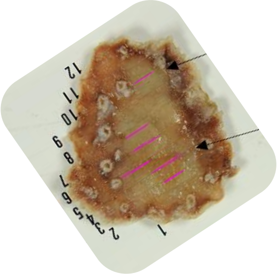

mapping image

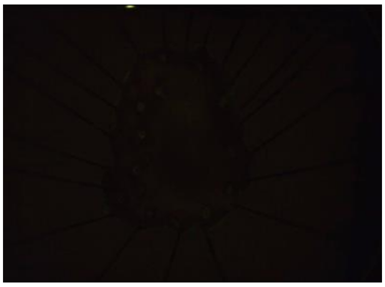

before

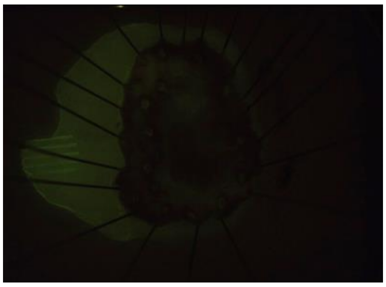

just after

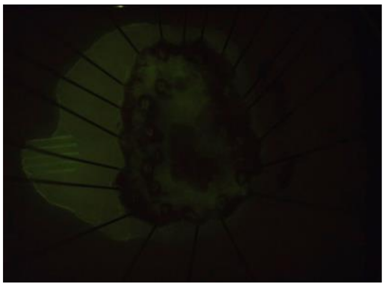

1 min

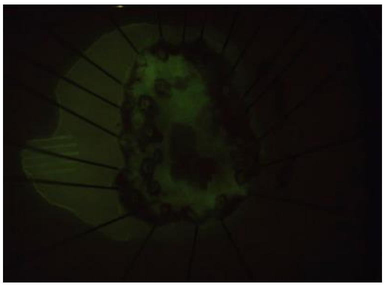

3 min

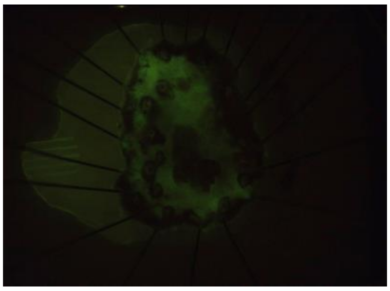

5 min

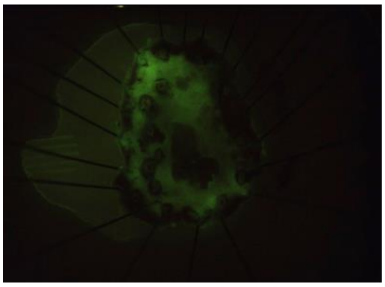

10 min

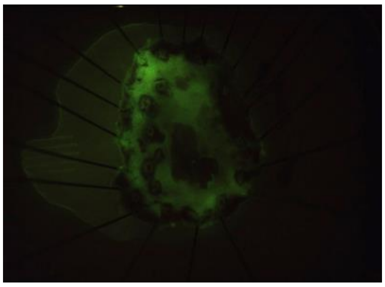

15 min

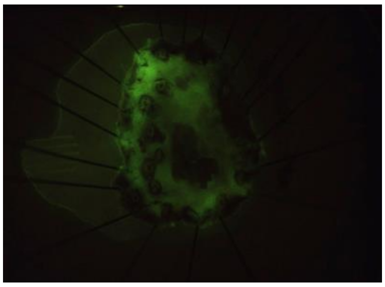

20 min

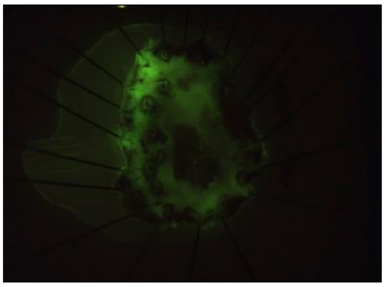

30 min

Representative successful case #3

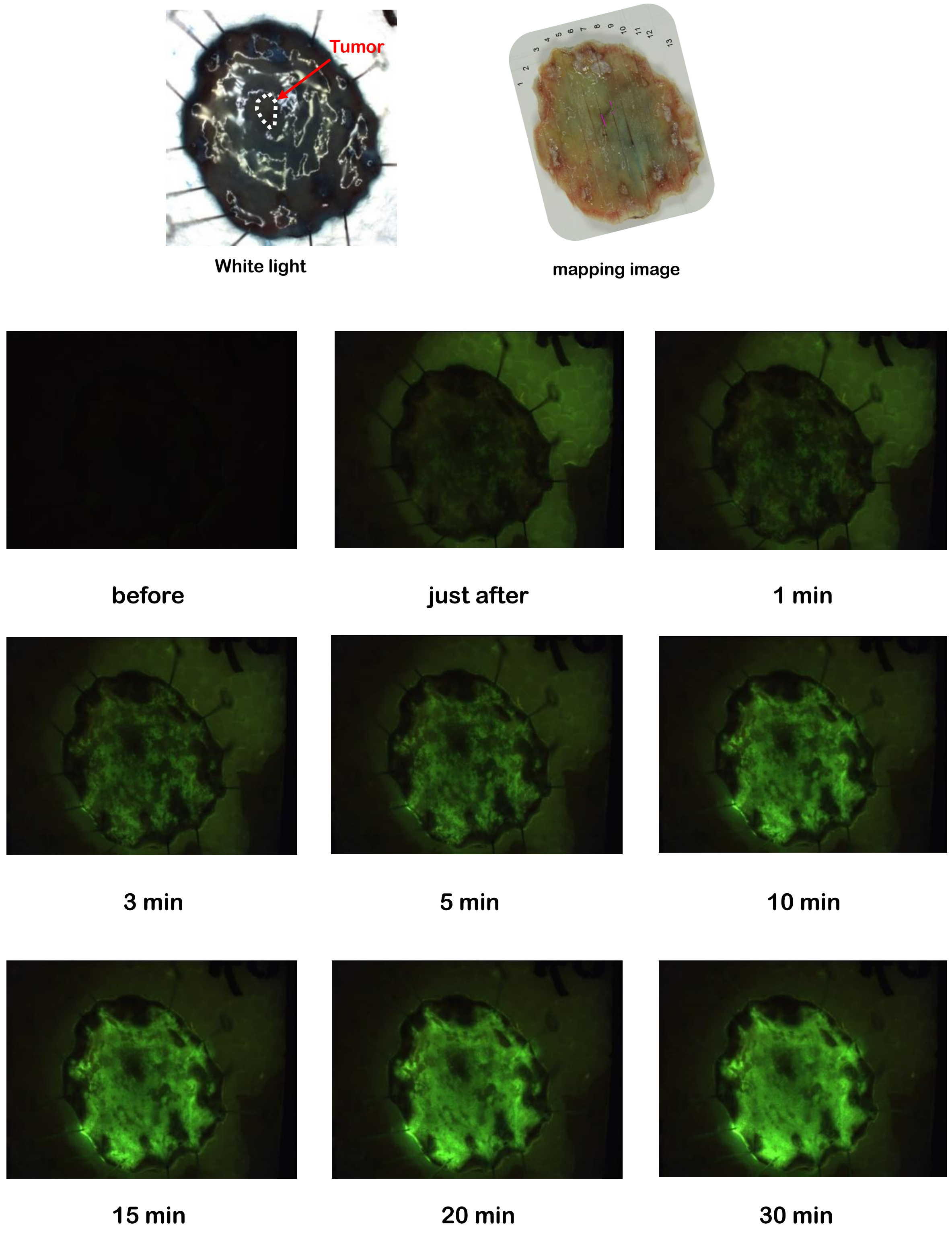

Figure S15. Three representative successful cases of detection of gastric cancer specimens with KH-HMRG. 50 µM KH-HMRG in phosphate-buffered saline was sprayed onto samples and the fluorescence increase was monitored with a Discovery imaging system. In mapping images, tumour regions are indicated with pink lines. Tumour regions were visualized by negative staining.

Representative failed case #1

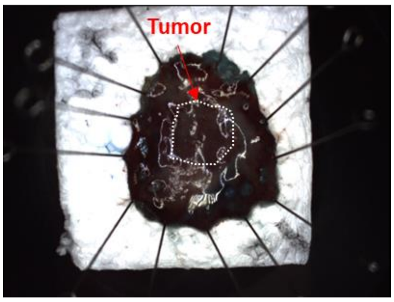

White light image

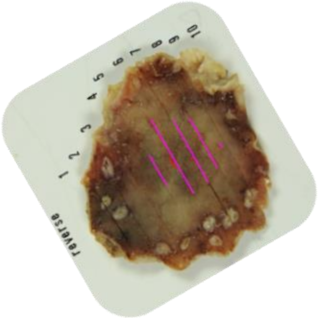

Mapping image

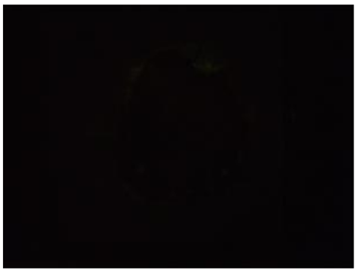

before

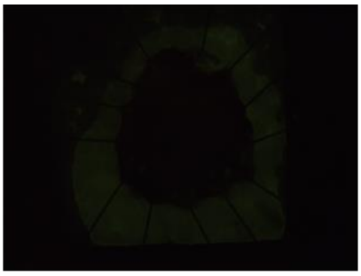

just after

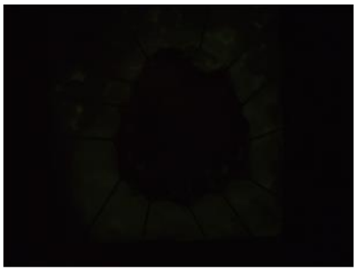

1 min

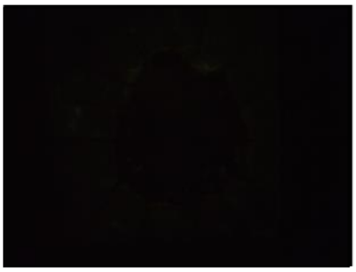

3 min

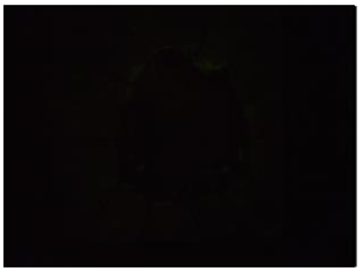

5 min

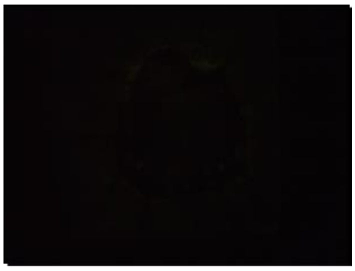

10 min

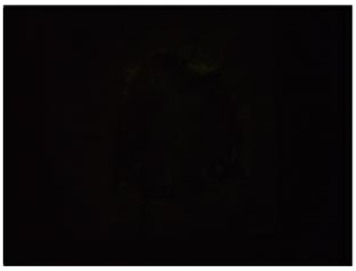

15 min

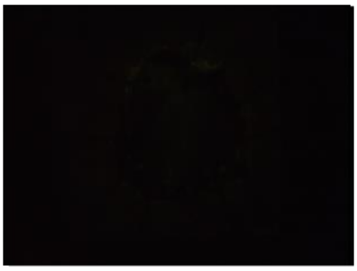

20 min

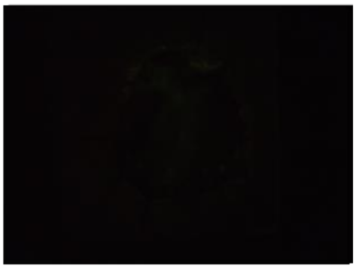

30 min

Representative failed case #2

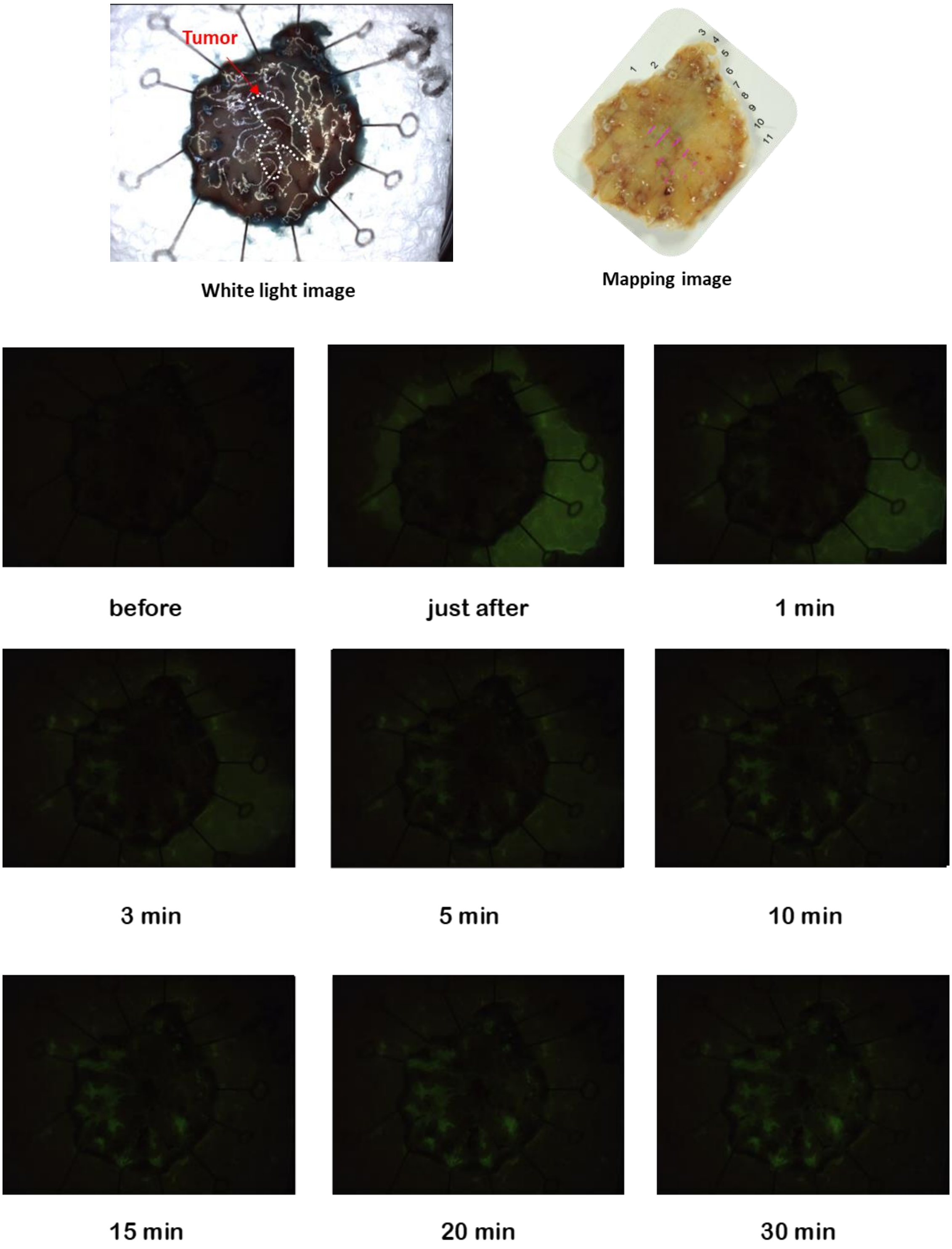

Figure S16. Two representative cases of failed detection of gastric cancer specimens with KH-HMRG. 50 µM KH-HMRG in phosphate-buffered saline was sprayed onto samples and the fluorescence increase was monitored with a Discovery imaging system. In mapping images, tumour regions are indicated with pink lines. No fluorescence increase was observed in tumour or non-tumour regions (case 1), or a small increase in in non-tumour regions compared to no fluorescence in tumour region was seen (case 2).

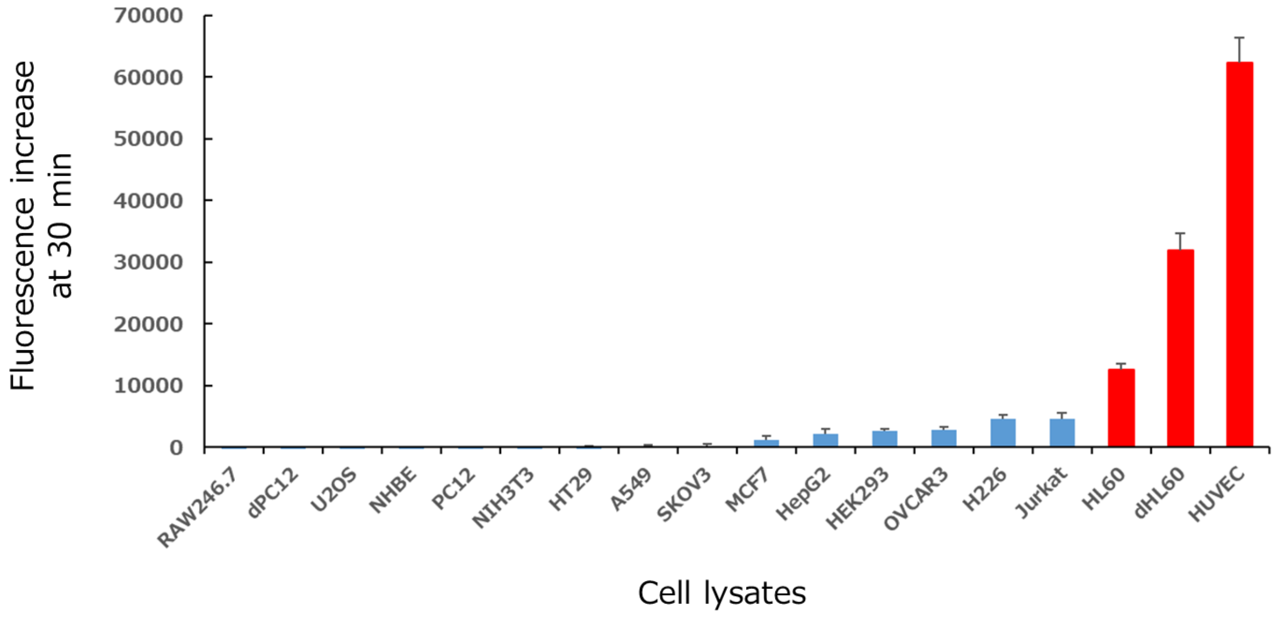

Figure S17. Reactivity with KH-HMRG of various culture cell lysates.

Fluorescence increase of 1 μM KH-HMRG in phosphate-buffered saline with 1 μg each culture cell lysate was calculated at 30 min. Incubated at 37 ºC. Error bars represent S.E. (n = 4). All assays were carried out at 37 °C in 20 μL total volume of phosphate-buffered saline (pH 7.4) containing 0.1 % DMSO as a co-solvent. Excitation/emission wavelengths = 485/535 nm. Lysates of HL60, dHL60 and HUVEC showed high reactivity with KH-HMRG (highlighted in red).

List of proteins included in the fluorescent spot of the DEG assay (Figure 5c)

1	Ubiquitin-like modifier-activating enzyme 1 OS=Homo sapiens GN=UBA1 PE=1 SV=3
2	Keratin, type I cytoskeletal 10 OS=Homo sapiens GN=KRT10 PE=1 SV=6
3	Cluster of Keratin, type II cytoskeletal 2 epidermal OS=Homo sapiens GN=KRT2 PE=1 SV=2 (K22E_HUMAN)
3.1	Keratin, type II cytoskeletal 2 epidermal OS=Homo sapiens GN=KRT2 PE=1 SV=2
3.2	Keratin, type II cytoskeletal 5 OS=Homo sapiens GN=KRT5 PE=1 SV=3
4	Keratin, type II cytoskeletal 1 OS=Homo sapiens GN=KRT1 PE=1 SV=6
5	Protein disulfide-isomerase OS=Homo sapiens GN=P4HB PE=1 SV=3
6	Keratin, type I cytoskeletal 9 OS=Homo sapiens GN=KRT9 PE=1 SV=3
7	Aminopeptidase N OS=Homo sapiens GN=ANPEP PE=1 SV=4
8	Glyceraldehyde-3-phosphate dehydrogenase OS=Homo sapiens GN=GAPDH PE=1 SV=3
9	Plastin-2 OS=Homo sapiens GN=LCP1 PE=1 SV=6
10	Cluster of 14-3-3 protein zeta/delta OS=Homo sapiens GN=YWHAZ PE=1 SV=1 (1433Z_HUMAN)
10.1	14-3-3 protein zeta/delta OS=Homo sapiens GN=YWHAZ PE=1 SV=1
10.2	14-3-3 protein theta OS=Homo sapiens GN=YWHAQ PE=1 SV=1
10.3	14-3-3 protein gamma OS=Homo sapiens GN=YWHAG PE=1 SV=2
10.4	14-3-3 protein beta/alpha OS=Homo sapiens GN=YWHAB PE=1 SV=3
10.5	14-3-3 protein epsilon OS=Homo sapiens GN=YWHAE PE=1 SV=1
11	Actin, cytoplasmic 1 OS=Homo sapiens GN=ACTB PE=1 SV=1
12	Serum albumin OS=Homo sapiens GN=ALB PE=1 SV=2
13	Calreticulin OS=Homo sapiens GN=CALR PE=1 SV=1
14	L-lactate dehydrogenase B chain OS=Homo sapiens GN=LDHB PE=1 SV=2
15	Xaa-Pro aminopeptidase 1 OS=Homo sapiens GN=XPNPEP1 PE=1 SV=3
16	Myeloperoxidase OS=Homo sapiens GN=MPO PE=1 SV=1
17	Malate dehydrogenase, cytoplasmic OS=Homo sapiens GN=MDH1 PE=1 SV=4
18	Lupus La protein OS=Homo sapiens GN=SSB PE=1 SV=2
19	Heat shock 70 kDa protein 4 OS=Homo sapiens GN=HSPA4 PE=1 SV=4
20	60 kDa SS-A/Ro ribonucleoprotein OS=Homo sapiens GN=TROVE2 PE=1 SV=2

Table S4. List of proteins detected by LC-MS/MS analysis of the fluorescent spot in the DEG assay. Enzymes that have aminopeptidase/protease activities are highlighted in green.

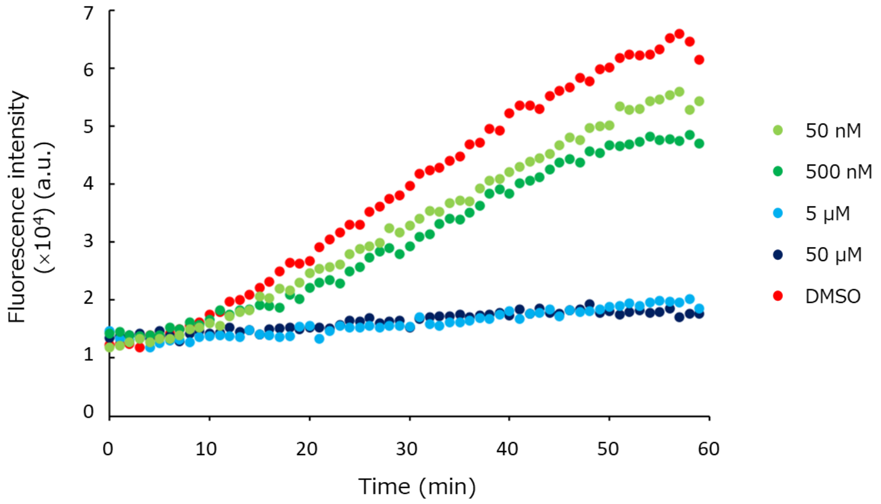

Figure S18. Enzyme assay of KH-HMRG with dHL60 lysate in the presence of inhibitor.

1 μM KH-HMRG was reacted with 2.5 μg dHL60 lysate in the presence or absence of bestatin (APN inhibitor) at various concentrations (n = 4). All assays were carried out at 37 °C in 20 μL total volume of phosphate-buffered saline (pH 7.4) containing 0.2 % DMSO as a co-solvent. Excitation/emission wavelengths = 485/535 nm.

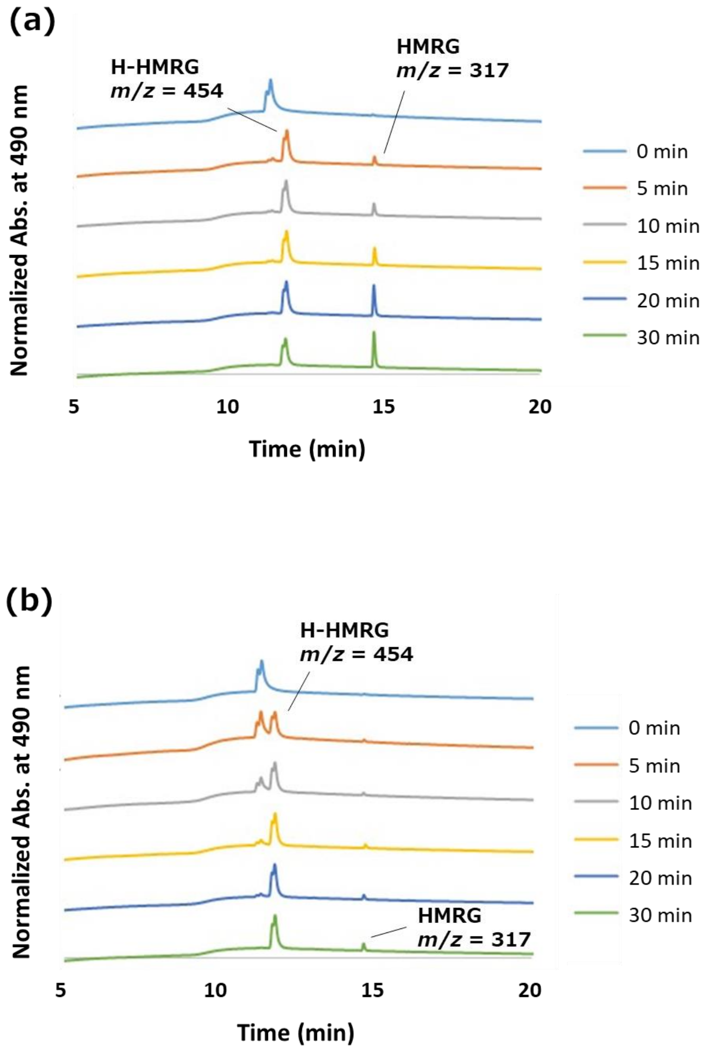

Figure S19. LC-MS analysis of reaction mixtures of KH-HMRG and (a) APN or (b) dHL60 lysate.

To a 20 µL of 10 µM probe solution in phosphate-buffered saline (pH 7.4) containing 0.1 % DMSO as a co-solvent, 25 ng of APN or 500 ng dHL60 lysate was added. The reaction mixture was incubated at 37 ºC and, at each indicated time point an equal volume of 10 % formic acid in MeOH was added to quench the enzymatic reaction. The mixture was analyzed by LC-MS (eluent; 0.1 % formic acid in H_2O/MeCN = 99/1 for 5 min, then to 5/95 in 15 min in linear gradient). Chromatograms detected at 490 nm are shown.

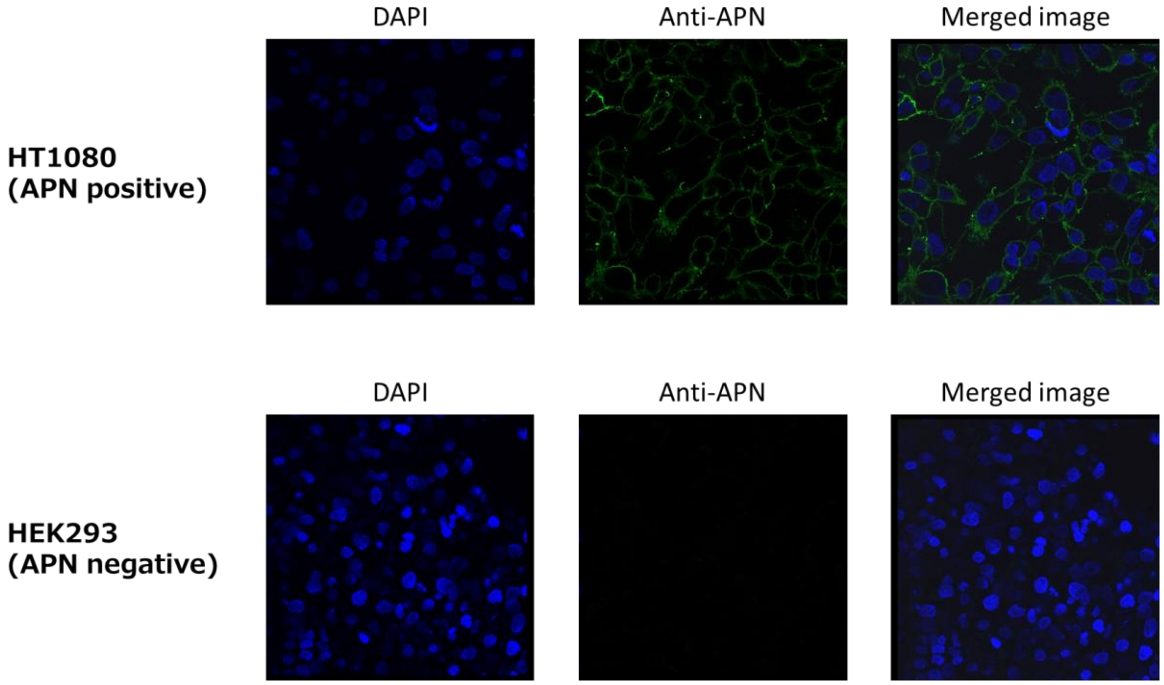

Figure S20. Confirmation of APN expression in HT1080 or HEK293 by immunocytochemistry.
Anti-CD13 antibody (abcam, 7417) and anti-mouse IgG H&L (Alexa Fluor® 488) (abcam, 150105) were used as 1st and 2nd antibody, respectively. Selective expression of APN in HT1080 was confirmed.

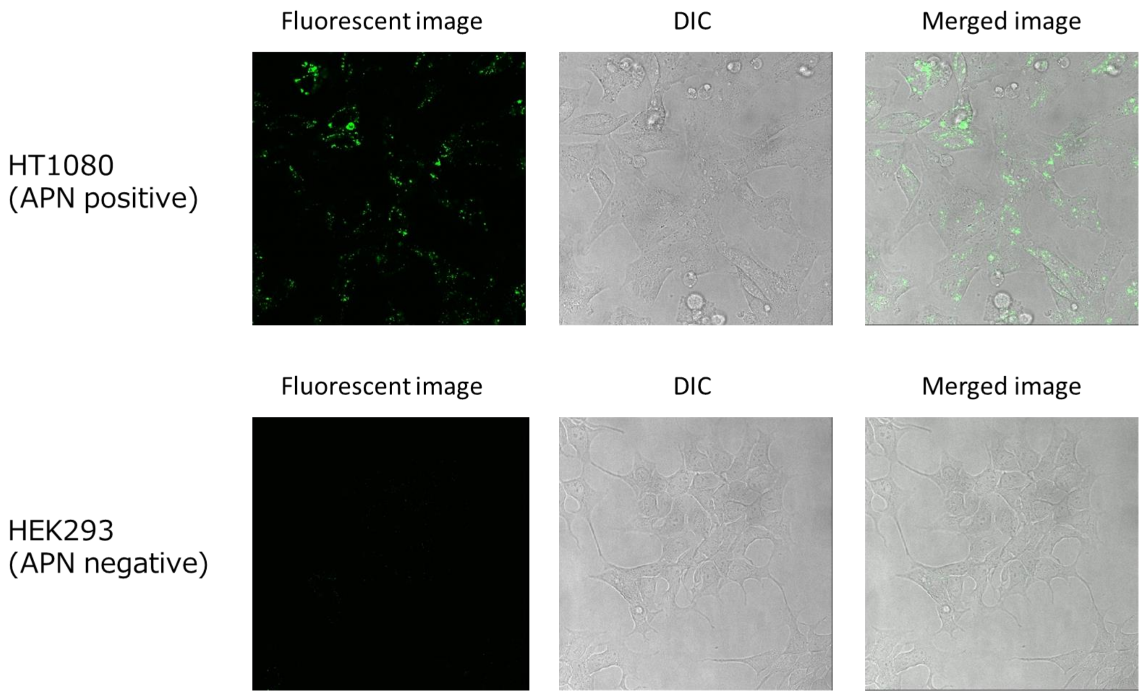

Figure S21. Live-cell imaging of APN activity with KH-HMRG.

Fluorescence images of HT1080 or HEK293 cells with 100 nM KH-HMRG in HBSS after incubation for 30 min at 37 ºC.

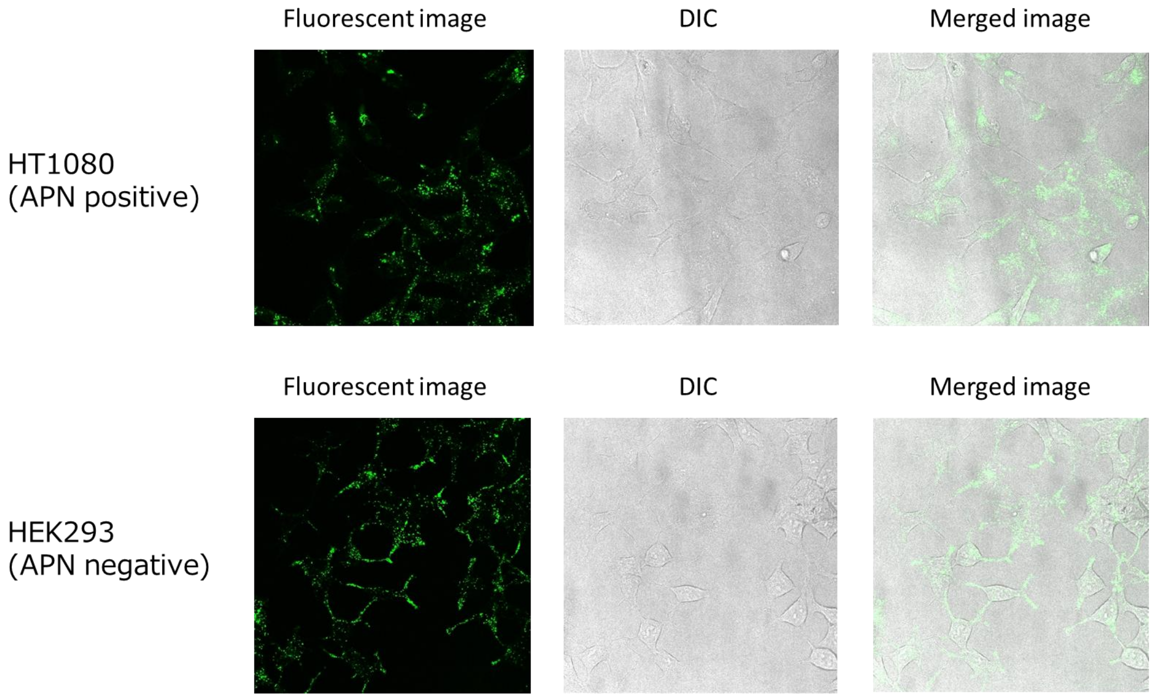

Figure S22. Live-cell imaging of APN activity with A-HMRG.

Fluorescence images of HT1080 or HEK293 cells with 100 nM A-HMRG in HBSS after incubation at 37 ºC for 30 min.

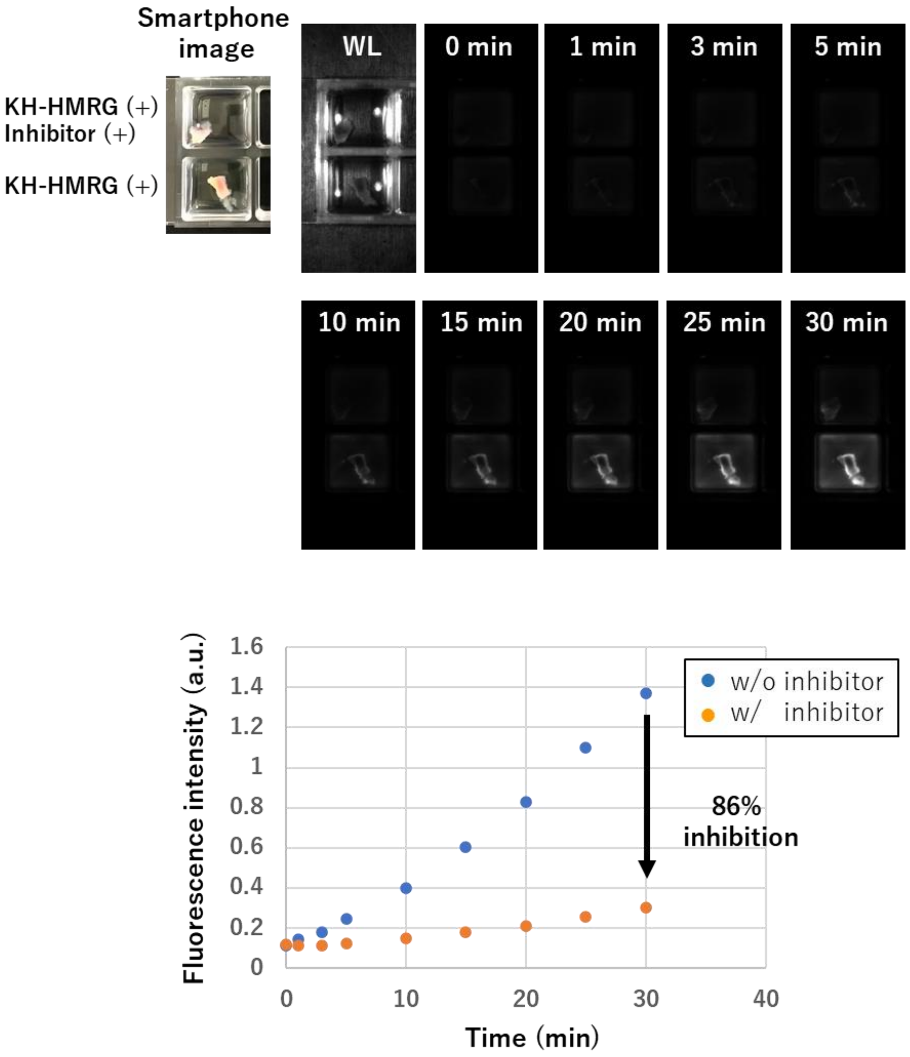

Figure S23. Ex vivo fluorescence imaging of gastric normal ESD samples with KH-HMRG in the absence or presence of APN inhibitor. Gastric normal ESD samples were incubated with 50 µM KH-HMRG in DPBS(-) in the absence or presence of 50 µM bestatin, and the fluorescence increase was monitored with a Maestro imaging system. Fluorescence images at 550 nm were extracted and fluorescence intensities were calculated for each sample. The fluorescence increase was clearly inhibited in the presence of bestatin.

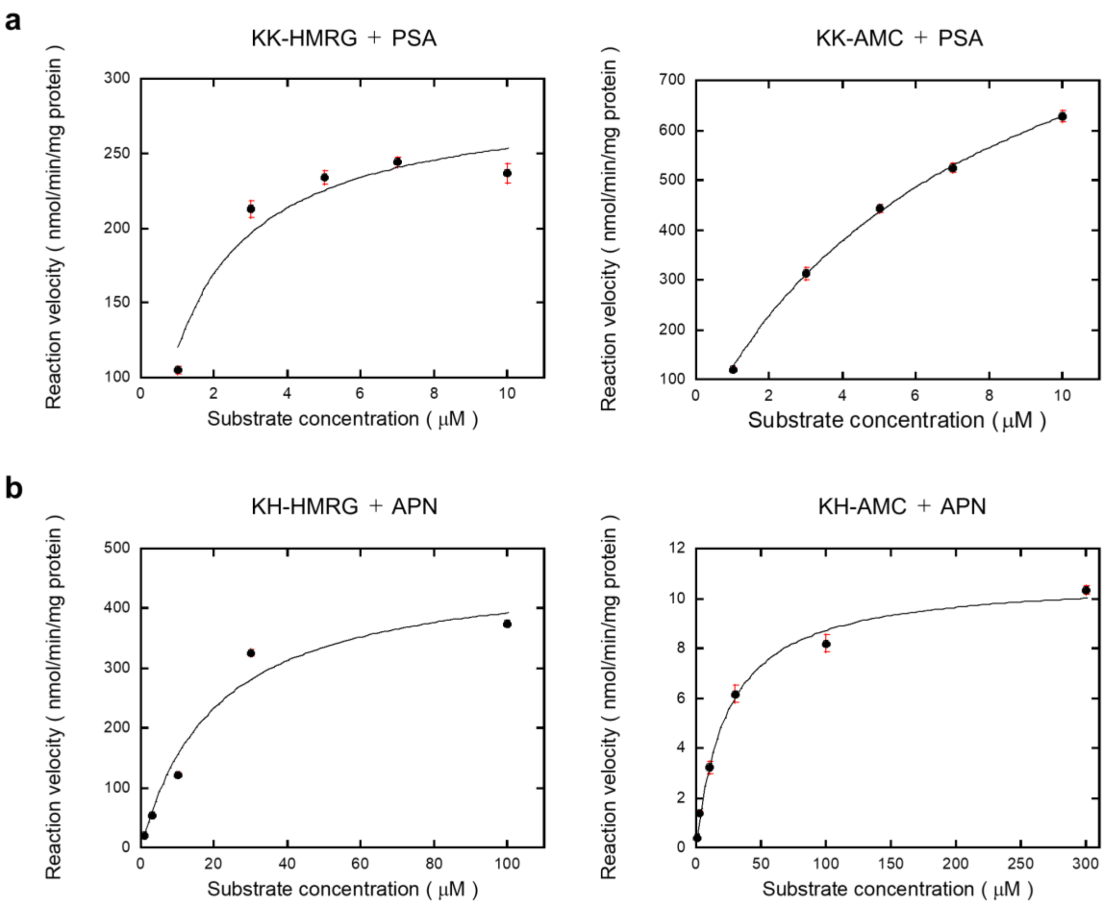

Figure S24. Michaelis-Menten plots for evaluation of the reactivities of HMRG-based and AMC-based substrates. (a) Kinetic assay of KK-HMRG and KK-AMC with PSA. (b) Kinetic assay of KH-HMRG and KH-AMC with APN. Assays were performed in PBS (-) containing 500 μM Triton X-100 at 37ºC. In the case of KK-HMRG, product inhibition was observed in the concentration range over 10 μM. Error bars represent S.E. (n = 4).

Substrate	K_m (μM)	k_{cat} (s^{-1})	k_{cat}/K_m ($s^{-1}μM^{-1}$)
KK-HMRG	1.40	0.481	0.344
KK-AMC	7.72	1.86	0.241

Table S5. Comparison of kinetic parameters of KK-HMRG and KK-AMC with PSA. k_{cat} values were calculated assuming the molecular weight of PSA to be 100 kDa.

Substrate	K_m (μM)	k_{cat} (s^{-1})	k_{cat}/K_m ($s^{-1}μM^{-1}$)
KH-HMRG	20.3	0.819	0.0403
KH-AMC	23.8	0.0187	0.000786

Table S6. Comparison of kinetic parameters of KH-HMRG and KH-AMC with APN. k_{cat} values were calculated assuming the molecular weight of APN to be 104 kDa.

Supplementary References

1. K. Yoshioka, T. Komatsu, A. Nakada, J. Onagi, Y. Kuriki, M. Kawaguchi, T. Terai, T. Ueno, K. Hanaoka and T. Nagano, *Journal of the American Chemical Society*, 2015, **137**, 12187-12190.
2. A. Japanese Gastric Cancer, *Gastric Cancer*, 2017, **20**, 1-19.
3. A. Japanese Gastric Cancer, *Gastric Cancer*, 2021, **24**, 1-21.
4. T. Sakai, T. Matsunaga, Y. Yamamoto, C. Ito, R. Yoshida, S. Suzuki, N. Sasaki, M. Shibayama and U.-i. Chung, *Macromolecules*, 2008, **41**, 5379-5384.
5. A. B. Hauert, S. Martinelli, C. Marone and V. Niggli, *The international journal of biochemistry & cell biology*, 2002, **34**, 838-854.
6. Y. Kuriki, M. Kamiya, H. Kubo, T. Komatsu, T. Ueno, R. Tachibana, K. Hayashi, K. Hanaoka, S. Yamashita, T. Ishizawa, N. Kokudo and Y. Urano, *Journal of the American Chemical Society*, 2018, **140**, 1767-1773.
7. M. Sakabe, D. Asanuma, M. Kamiya, R. J. Iwatate, K. Hanaoka, T. Terai, T. Nagano and Y. Urano, *Journal of the American Chemical Society*, 2012, **135**, 409-414.
8. T. Komatsu, K. Hanaoka, A. Adibekian, K. Yoshioka, T. Terai, T. Ueno, M. Kawaguchi, B. F. Cravatt and T. Nagano, *Journal of the American Chemical Society*, 2013, **135**, 6002-6005.
